# Performance of active and passive ambulatory assessment measures and mood monitoring in bipolar disorder: a systematic review

**DOI:** 10.1186/s40345-025-00407-5

**Published:** 2026-01-23

**Authors:** Laurence Astill Wright, Eduard Bakstein, Kate Saunders, Boliang Guo, Richard Morriss

**Affiliations:** 1 Institute of Mental Health, University of Nottingham, Nottingham, UK; 2 Centre for Academic Mental Health, Population Health Sciences, University of Bristol, Bristol, UK; 3 National Institute of Mental Health in Czechia, Klecany, Czechia; 4 Department of Cybernetics, Czech Technical University in Prague, Prague, Czechia; 5 Department of Psychiatry, Warneford Hospital, University of Oxford, Warneford Lane, Oxford, UK; 6 Oxford Health NHS Foundation Trust, Warneford Hospital, Oxford, OX3 7JX UK; 7 NIHR ARC East Midlands, University of Nottingham, Nottingham, UK; 8 Nottingham NIHR Biomedical Research Centre, University of Nottingham, Nottingham, UK; 9 NIHR MindTech Medical Technology Collaborative, University of Nottingham, Nottingham, UK

**Keywords:** Bipolar, Depression, Ambulatory assessment, EMA, Ecological momentary assessment, Mood-tracking, Mood-monitoring, Self-monitoring

## Abstract

**Background:**

Ambulatory assessment uses digital technology to capture real-time data on mood, mental state and behaviour. It has the potential to enhance traditional clinical outcome measures, but the practical application of these tools fundamentally depends on their performance.

**Aims:**

This systematic review aimed to assess the performance of active and passive ambulatory assessment and mood monitoring outcome measures in non-randomised and randomised studies in bipolar disorder over 3 months or longer. We aimed to evaluate their performance against established clinical measures and through inter-ambulatory assessment comparisons.

**Methods:**

Systematic review (PROSPERO: CRD42023396473) of performance of mood monitoring and ambulatory assessment protocols in RCTs and non-randomised studies in bipolar disorder. Identified studies were assessed for risk of bias. Due to the very high heterogeneity in included studies and performance metrics we were not able to aggregate the data via meta-analysis.

**Results:**

The review included 42 studies with a combined sample of 7,813 participants. We included 28 distinct ambulatory assessment protocols which reported 487 different smartphone-based performance metrics. The considerable variability and inconsistency across these metrics limited our ability to make definitive comparisons of performance. Overall, some active ambulatory assessment approaches showed good performance when compared with established clinical measures. There was a paucity of data examining the performance of passive ambulatory assessment measures. Most studies were rated as having low to moderate risk of bias.

**Conclusions:**

While ambulatory assessment holds significant promise, current evidence fails to establish the validity and reliability of passive ambulatory assessment to measure mood. The substantial methodological variation—particularly in how performance metrics are defined and reported—limits meaningful comparison and replication. Greater consistency in ambulatory assessment design and reporting standards is essential to support reliable evaluation and broader adoption of these behavioural assessment tools.

**Supplementary Information:**

The online version contains supplementary material available at 10.1186/s40345-025-00407-5.

## Introduction

Ambulatory assessment comprises a spectrum of methods that use mobile technology to study individuals regularly in their natural environment and in real time (Trull and Ebner-Priemer 2013). This term is broad and many of the ambulatory assessment protocols could also be classified as using EMA (ecological momentary assessment), mood monitoring, mood tracking or remote measurement technology. These terms often overlap. Remote measurement technology collects passive data in the background using tools such as wearable devices e.g. smartwatches, mobile phone sensors. EMA often repeatedly collects self-report data in the moment and multiple times per day (aan het Rot et al. 2012). Active ambulatory assessment relies on the participant self-reporting their mood or behaviour while passive ambulatory assessment automatically collects this data without the user manually inputting any data instead passively running in the background – often synonymous with remote measurement technology. There is significant variation in the frequency of data collection with some ambulatory assessment protocols collecting data daily, multiple times per day or continuously. The purpose of the study and the nature of the behaviour/psychopathology assessed dictates the frequency of the mood assessment and the assessment methodology used (e.g. GPS location vs visual analogue scale of mood). Mood monitoring can serve both as an intervention (in randomised and non-randomised studies) and as a means of measuring outcomes (again both in RCTs and non-randomised studies).

This technology is particularly promising in the assessment and treatment of bipolar disorder (BD). Using new technology to improve clinical assessment in both research and practice promises large improvements in efficiency, flexibility and usability. The possibility that we could measure mood, psychopathology and behaviour frequently, or even continuously, may allow us to gather the data required to offer personalised digital solutions and care to people with BD. We know that shifts in psychopathology can happen quickly and so conventional research measures, often assessing symptoms over the preceding week or month, may miss some of this dynamic change that is common in people with BD (Grunze and Born 2020; McIntyre et al. 2020). Established validated tools assessing mood are extremely useful but lack the flexibility that may be required to measure dramatically changing behaviour associated with mental distress, relapse and risk-taking behaviour in BD. In addition to improving assessment, intensive individual assessment may allow us to utilise this individual level of observation to offer novel and targeted management. We already know that improving awareness of early warning sign symptoms in people with BD can prevent relapse, hospitalisation and improve functioning (Morriss et al. 2007) and higher quality data may facilitate the development of improved and digitised mood monitoring based interventions. Furthermore, repeated and frequent assessment, particularly in the emergence of psychopathology, may improve prediction and prognosis as well as treatment outcome through digital phenotyping (Nelson et al. 2017; Brietzke et al. 2019) and this has the potential to improve the long diagnostic delay in people with BD (Lublóy et al. 2020). Furthermore, many people with BD are enthusiastic about the use of ambulatory assessment for treatment and monitoring (Wright 2024). Any potential usability, however, depends on the measures having good performance and there are currently concerns around the performance of these ambulatory assessment measures. Performance is thus key to subsequent implementation.

The review uses a broader scope than previous reviews and here we examine a wide range of ambulatory assessment protocols in a specific disorder (bipolar disorder) over a longer period of time (3 months minimum), rather than for example just assessing protocols that self-identify as mood-monitoring protocols, or just those delivered via IT platforms. BD is characterised by temporal mood changes happening over weeks and months a broader scope is necessary to consider the possible uses of this wide-ranging technology in this particular application. This is the first systematic review that we are aware of that examines the performance and use of ambulatory assessment as a treatment outcome in BD and quantitatively evaluates the performance of these protocols. The aim of this systematic review is to assess ambulatory assessment and mood monitoring as an outcome in randomised and non-randomised studies in people with BD. Specifically we will examine performance, adherence, compliance, dropout, usability and properties ambulatory assessment/mood tracking protocols. We evaluate the performance of these measures against established clinical measures and through inter-ambulatory assessment comparisons.

## Methods

We used a methodology based on the Cochrane Handbook for Systematic Reviews of Interventions and completed a Preferred Reporting Items for Systematic Reviews and Meta-Analysis (PRISMA) checklist. The study was pre-registered with the International Prospective Register of Systematic Reviews (PROSPERO: CRD42023396473 (PROSPERO 2023)).

### Inclusion criteria

We included studies if they met the following criteria: included self-monitoring/ambulatory assessment/EMA/repeated symptom assessment in people with BD over a minimum period of 3 months with rating of symptoms weekly at a minimum. This could use ambulatory assessment as either an intervention or as a research outcome using a randomized or non-randomised design. We only included studies with 20 or more participants with BD (Excellence NIfHaC 2021). The studies should either use a validated measure of mood or validate the chosen measure with a validated mood measure. The studies could be published in any language and could be digital or non-digital, although we acknowledged that the majority of studies would utilize digital technologies. We searched the grey literature (e.g. conference abstracts, dissertations, policy literature, reports via ProQuest and Google Scholar – full details below) for unpublished studies which were eligible for inclusion.

### Search strategy and selection criteria

The complete search strategy in listed in the appendix. We searched Ovid MEDLINE, EMBASE, PsychINFO, SCOPUS, IEE Xplore, Proquest SciTech Collection, Proquest dissertations and theses global, Google Scholar using the search terms. The initial search was conducted on 03/03/23 and then updated on 28/10/24. All abstracts were appraised by two independent screeners (LAW, GM, GS, KA, DP, GON) and any disagreements were discussed and a consensus arrived upon, with adjudication by a third independent screener if required. We acquired the full text of any potentially relevant papers and if we were unable to source the full text of the study we then contacted the corresponding author to request the paper. To determine if potentially relevant studies met the inclusion criteria the full text was reviewed separately by two authors, again with discussion and consensus with a third reviewer if necessary. All papers for inclusion were reference checked along with relevant systematic reviews (Antosik-Wojcinska et al. 2020; Dubad et al. 2018; Palmier-Claus et al. 2021; Faurholt-Jepsen et al. 2016a, 2019a; Watt et al. 2020; Ortiz et al. 2021; Malhi et al. 2017; Wenze and Miller 2010; Koenders et al. 2015). If we were unable to access the full text of a paper the authors were emailed to request the paper. Key authors were also emailed to see if the inclusion of any ongoing unpublished studies could be included. Performance of the ambulatory assessment/mood tracking procedures used were often published in separate papers and cited within the main publication. We also emailed authors to identify separate validation studies.

### Data extraction

Two independent reviewers extracted data from studies meeting the inclusion criteria using identical data extraction forms. Irregularities in the data extraction were discussed and any discrepancy resolved with discussion.

### Assessment of study bias

The Cochrane Collaboration’s tool for assessing the risk of bias in non-randomised studies of interventions (Sterne et al. 2016) was used for each study. Risk of bias was assessed by two independent reviewers (L.A.W & GS) and any disagreement resolved via discussion.

### Synthesis of results

We grouped studies together, where possible, according to the variable assessed e.g. mood state (bipolar depression, mania/hypomania), functioning and pooled data in a meta-analysis. We conducted analysis via mood state as we hypothesized the performance for different mood states may vary. Results of each primary study were pooled by means of inverse variance weighted approach with random effects model, informed by examining the between studies heterogeneities. Correlation coefficients were transformed into Fisher Z score, and proportion was transformed into logit value for pooling. Pooled results were then back transformed to relevant original scale. Stata metan code was used to perform the analysis for correlation and proportion data.

## Results

The search identified 23,515 papers. No studies that were not in English were found to meet the inclusion criteria. Following title and abstract screening 21,638 were excluded resulting in a total of 758 papers being reviewed in full. A total of 42 papers met the eligibility criteria and were included in the review. The 42 included studies included 7,813 participants. Table 1 and Supplementary Table 1 display detailed characteristics of the studies and the ambulatory assessment protocols used.

**Table 1:** Ambulatory assessment protocols of included non-randomised and randomised studies

Ambulatory assessment protocols for non-randomised studies							
Study	Ambulatory assessment/Mood tracking procedure	Ambulatory assessment duration	Active or passive Ambulatory assessment	Analogue or digital ambulatory assessment	Adherence to ambulatory assessment	Study attrition	Validity of ambulatory assessment measure/psychometric properties
Anyz et al. 2021	ASERT mood self-reports weekly—10 items that map dperessive, manic and nonspecific symptoms on a likert scale	18 months	Active	Digital	78.1%	Not reported	Performance of ASERT to predict depressive/manic relapse: (Anýž et al. 2021)
Hidalgo-Mazzei et al. 2016	Self-report 5 item test assessing mood, energy, sleep duration, medication adherence and irritability—daily. DSM-5 criteria for manic/depressive episodes—weekly	3 months	Active	Digital	Whole sample interactions with app: 77/90 days mean (26.2). Interaction rate 1.3 times per day	1 month attrition: 6%, 2 month attrition: 18%, 3 month attrition: 26%	Convergent validity of SIMPLe daily test score with YMRS/HDRS: Hidalgo-Mazzei et al. 2016
Hidalgo-Mazzei et al. 2018	Self-report 5 item test assessing mood, energy, sleep duration, medication adherence and irritability—daily. DSM-5 criteria for manic/depressive episodes—weekly	12 months	Active	Digital	Average daily interaction of users while using the app: 1.8 times per day. Average weekly engagement with app: 25% completed all tasks, 16% completed three quarters, 15% completed half, 44% completed < 25% of tasks	1 month: 34.8%, 6 months: 76%, 12 months: 81%	Nil validation
Garcia-Estela et al. 2022	Self-report 5 item test assessing mood, energy, sleep duration, medication adherence and irritability—daily. DSM-5 criteria for manic/depressive episodes—weekly	6 months	Active	Digital	22.5% never used the app, 70.9% regular users, 6.6% occasional users (engagement < 12%)	13.8% used app for > 100 days, 86.2% did not. Survival probability after 1 month 67.4% (95% CI 62.7%—72.4%), 3 months 43% (95% CI 38.1%—48.5%), 6 months 28% (95%CI %23.6—%33.2)	Nil validation
Bauer et al. 2023	ChronoRecord—daily mood via 100 point VAS, sleep, life events, menstrual data, psychiatric medication, weekly—weight	Average follow up: 227 days	Active	Digital	12% didn’t return any data. Average follow up: 227 days	12% didn’t return any data	Concurrent validity of analogue ChronoRecord & validated measures of mood and functioning: (Bauer et al. 2004) Concurrent validity of electronic ChronoRecord & validated measures of mood and functioning: (Whybrow et al. 2003) Concurrent validity of electronic ChronoRecord & validated measures of mood and functioning: (Bauer et al. 2008)
Bos et al. 2022	5 × EMA smartphone assessments daily—29 items assessing monetary mood, symptoms, sleep and activities. Weekly ASRM, QIDS-SR-16 delivered via RoQua platform	4 months	Active	Digital	76% (number of assessments: 467, range: 197–869)	0	Linear mixed model investigating the relationship between mood states and weekly symptoms scores on the same day of weekly assessment: (Moraga et al. 2023)
Bowden et al. 2021	KIOS app—self report assessment of 8 different symptoms e.g. sadness/pessimism and delivery of guidance in relation to symptom change	3 months	Active	Digital	85% performed at least 1 assessment weekly	15%	Nil validation
Dominiak et al. 2022	BDmon app—Passive ambulatory assessment: phone/SMS logs, participant speech information extracted from daily phone calls, Active ambulatory assessment: self report mood	mean: 208 days (SD: 32)	Active & Passive	Digital	79.6% of participants had usable data. Completeness of self-report mood and sleep 27.3% and 27.5 respectively. Completeness of passive data varied from 61.1% to 100%	20.4% with no smartphone data over required periods	Associations between active and passive smartphone data and the HDRS-17/YMRS: (Dominiak et al. 2022)
Emden et al. 2021	ReMAP system—Active ambulatory assessment—single mood likert scale, single item sleep scale assessing self-report sleep time, voice sample—weekly. Passive ambulatory assessment: step-count, GPS location, accelerometer—continuous	12 months	Active & passive	Digital	33.09% still had app installed at 12 months. Median duration of install—135 days (IQR: 111). Average rate of days on which a passive data event was sent was 73.40% (33.73%)	66.91%	Correlation between smartphone based BDI and non-smartphone based BDI: (Goltermann et al. 2021)
Stanislaus et al. 2020	Monsenso system—Active ambulatory assessment: daily smartphone self monitoring items—mood and activity level, HDRS-17 & YMRS every 3 days. Passive ambulatory assessment: Objective smartphone data—phone usage, call/SMS logs, step count	Median 106 days (IQR: 48–204)	Active & passive	Digital	80% provided > 1 month of passive data	4.2% attrition in BD group	Associations between self-reported mood instability using Monsenso system in participants with BD, unaffected first-degree relatives, and HC: (Stanislaus et al. 2020)
Lee et al. 2022	eMoodChart system—Active ambulatory assessment: self report daily mood and energy. Passive ambulatory assessment: Fitbit measuring step-count, heart rate, sleep, ambient light (android online)	mean: 279.7 days (SD: 263.5), median: 505 days (range: 72–1515)	Active & passive	Digital	54.5% wore activity trackers for at least 30 days	Not reported	Performance evaluation of a mood prediction model: (Lee et al. 2022)
Born et al. 2014	NIMH Life Chart Methodology—prospective —twice daily mood self-rating	Unclear—potentially 3 years	Active	Analogue	Not reported	Not reported	Correlation between NIMH Life Chart Methodology-prospective and validated mood measures: (Born et al. 2014)
Lieberman et al. 2011	MoodChart—daily self report mood via email/online, Social Rhythm Metric—activity level over the previous 7 days	mean: 84 (range: 42–90)	Active	Digital	Not reported	Not reported	Nil validation
Kupka et al. 2005	NIMH Life Chart Methodology—prospective — daily mood self-rating	1 year	Active	Analogue	Not reported	Not reported	Prospective NIMH-Life-Chart Method validation: (Denicoff et al. 2000). Relationship of LCM-p to CGI-BP: (Denicoff et al. 2002)
O’Rourke et al. 2021	Twice daily Bipolar Disorder Symptom Scale—time/GPS stamped	4 + months	Active & passive	Digital	196 responses per participant (range: 3–543), over an average of 145 consecutive days (range: 2–435)	Not reported	Associations between BD symptoms and PHQ-9: (O’Rourke et al. 2016). Associations between BDSx and self report/clinical assessment of mood: Osher et al. 2019(Osher et al. 2019)
Tseng et al. 2022	Active ambulatory assessment: daily mood, sleep duration. Weekly ASRM, DASS-21. Passive ambulatory assessment: GPS location	number of days on which participants performed self-assessments—mean: 94.25 days (median 52.5, range 2 to 398)	Active & passive	Digital	75% of participants performed self-assessments over 3 months. Incomplete data over 1 week: 35.87% of daily mood data, 47.45% of sleep duration data, 58.86% of GPS data. Incomplete data over 1 month: 49.50% of daily mood data, 65.38% of sleep duration data, 77.19% of GPS data	25% of participants did not perform self-assessments over 3 months	Associations between smartphone measured daily mood, sleep duration, GPS data and HDRS/ASRM: (Tseng et al. 2022). Associations (repeat-measures correlations) among daily mood, sleep duration and GPS data over different time frames: (Tseng et al. 2022)
Ebner-Priemer et al. 2020	MovisensXS system—Active ambulatory assessment: self-report mood, sleep diary. Passive ambulatory assessment: call/text logs, GPS data, velocity, step-count	12 months	Active & passive	Digital	97% attendance at two weekly assessments, 99% for passive ambulatory assessment, 89% of daily self report mood/sleep data	6.5%	Standardized factor loadings of the latent psychopathological outcome for depression and mania: (Ebner-Priemer et al. 2020) Multilevel SEM modelling of one latent digital phenotype predictor for each domain (sleep, activity, communicativeness): (Ebner-Priemer et al. 2020) Latent digital phenotype predictors related to same‐day latent psychopathological outcomes: (Ebner-Priemer et al. 2020)
Gideon et al. 2016	PRIORI system—records speech made on telephone calls. Weekly HDRS, YMRS	6–12 months	Active & passive	Digital	37 participants made 34,830 calls over 2,436 h	Not reported	Mood classification results comparing speech parameters with HDRS/YMRS using various models (AUCs in bold denote results significantly better than baseline via paired t-test, p-value: 0.05: (Gideon et al. 2016)
Schneider et al. 2022	MINDPAX—actigraphy measuring sleep data	3 months	Passive	Digital	BD: 71.4% submitted data for the duration of the study, HC: 96.1% submitted data for the duration of the study	28.6% attrition at 3 months	Group differences in sleep variables between BD and HC: (Schneider et al. 2022) Random forest classifier model results in participants whose data were not used during model training: (Schneider et al. 2022)
Scharer et al. 2015	PLC app—daily self report mood	18 months	Active	Digital	Not reported	Not reported	Spearman’s rank correlation coefficients comparing PLC daily self report mood and IDS-C/YMRS, in this study and other NIMH life chart studies: (Schärer et al. 2015)
van den Heuvel et al. 2018	PHR-BD system—including 9 modules covering: medical record, medication, treatment and medical passport, general information about BD, medical results/reports, platform to send messages to appointed clinician, mood chart with daily self report mood, personal crisis plan	12 months	Active	Digital	88.9% utilised mood chart, 66.7% utilised mood graph interface, 50% used crisis plan, messages module and medical/results module	41% at 12 months	Nil validation of PHR-BD
Arribas et al. 2018	AMoSS study system—Active ambulatory assessment: daily mood rating across categories of anxiety, elation, sadness, anger, irritability and energy using MoodZoom questionnaire, ASRM, QIDS-SR16, EQ-5D, GAD-7 assessed weekly. Ambulatory assessment: GPS, actigraphy, ambient light, call/SMS logs, heart rate via smartphone/Fitbit/GENEActive accelerometer/Proteus patch (heart rate data only for one week)	3 months, with 61 participants continuing for 12 months	Active & passive	Digital	81.2%	53.1% at 12 months	Accuracy and area under the ROC curve using pairs of diagnosis for classification (rather than all 3 groups): (Perez Arribas et al. 2018) Accuracy of signature based model to predict subsequent mood score using 20 consecutive observations: (Perez Arribas et al. 2018)
Lewis et al. 2023	Bipolar Disorder Research Network using True Colours: weekly ASRM/QIDS-16-SR	21 months	Active	Digital	73.5% of participants had available/sufficient data for 21 month duration analysis	26.4% at 21 months	ASRM/QIDS-SR16 are established measures—nil novel ambulatory assessment measures used
McKnight et al. 2017	OXTET-1 using True Colours—ASRM/QIDS-SR-16 delivered via weekly SMS/email	27.5 ± 22.5 months (range: 1–81)	Active	Digital	92.1%. Missing data ASRM: 7.9% (IQR: 0–4.1), QIDS-SR-16: 7.8% (0–2.7)	19.1% attrition	ASRM/QIDS-SR-16 are established measures—nil novel ambulatory assessment measures used
Ortiz et al. 2023	E-monitoring system—Active ambulatory assessment: daily rating of mood, anxiety, energy level using e-VAS. Weekly: PHQ-9, ASRM. Passive ambulatory assessment: Oura Health Oy 3 d accelerometer/hyroscope measuring activity, sleep, Infrared optical pulse measuring heart rate, heart rate variability	229.4 days (± 12.4)	Active & passive	Digital	Daily e-VAS: 74.6%, Weekly PHQ-9/ASRM: 78.5%, Passive measures: 79.5%	Not reported	Sensitivity and specificity to detect sleep/wake. Agreement with polysomnography to detect sleep phases: (Zambotti et al. 2019)
Ambulatory assessment protocols of randomised studies							
Bilderbeck et al. 2016	QIDS-SR-16, ASRM—administered weekly via TrueColours	12 months	Active	Digital	Compliance/adherence to weekly measures: Both groups: 73.65%, FIMM group: 76.6% (SD 22.9%), MIMM group: 70.7% (24.6%)	Total attrition: 27% at 12 months. Full relapse data over 12-month follow-up was available for 73% of the participants	Nil validation of this particular protocol but – QIDS-SR-16 & ASRM validation more broadly: (Rush et al. 2003; Altman et al. 1997)
Castle et al. 2010	Weekly telephone calls – weekly for 12 weeks	12 weeks	Active	Analogue	Not reported	Total attrition: 14% at 12 months, Intervention group: 24%, Control group: 5%	Nil validation of this ambulatory assessment protocol – telephone mood monitoring/contact used as an intervention not as an assessment method
Denicoff et al. 2002	NIMH Life Chart Methodology—prospective — daily mood self-rating	3 years	Active	Analogue	Not reported	Total attrition over 3 treatment phases: 44% attrition at 3 years	Prospective NIMH-Life-Chart Method validation: (Denicoff et al. 2000)
Depp et al. 2012	NIMH Life Chart Methodology—prospective — daily mood self-rating via paper and pen and via smartphone	3 months	Active	Analogue vs Digital	Mean observations in paper and pen charting 51.2, mean observations in smartphone charting 72.3	34% attrition at 3 months	Prospective NIMH-Life-Chart Method validation: (Denicoff et al. 2000)
Faurholt-Jepsen et al. 2015	Daily smartphone self monitoring—mood, sleep duration, medication taken, activity, irritability, mixed mood, cognitive problems, alcohol consumption, stress, menstruation, individualised EWS	6 months	Active & Passive	Digital	> 93% of patients randomised to intervention group self reported on a daily basis	Intervention group: 3% attrition over 6 months, control group: 3% attrition over 6 months	Associations between active and passive smartphone data (including self-reported mood) and established scales: (Faurholt-Jepsen et al. 2014) Sensitivity, specificity, PPV, NPV and AUC data of objective smartphone data in distinguishing between people with BD and healthy controls: Faurholt-Jepsen et al. 2022(Faurholt-Jepsen et al. 2019b) Associations between daily active/passive self-reported/monitored data and validated measures: Faurholt-Jepsen et al. 2016(Faurholt-Jepsen et al. 2016b)
Faurholt-Jepsen et al. 2019	Daily smartphone self monitoring items—mood, sleep duration, medication taken, activity, irritability, mixed mood, cognitive problems, alcohol consumption, stress, menstruation, individualised EWS, anxiety, self-defined personal parameters, free-text note. Objective smartphone data—phone usage, social activity, step count, GPS location	9 months	Active & Passive	Digital	Over 9 months patients in the intervention group adhered to the daily self-monitoring 72.6% of the days	Intervention group: 7% attrition at 9 months, control group: 7% attrition at 9 months	Associations between active and passive smartphone data (including self-reported mood) and established scales: Faurholt-Jepsen et al. 2014(Faurholt-Jepsen et al. 2014) Sensitivity, specificity, PPV, NPV and AUC data of objective smartphone data in distinguishing between people with BD and healthy controls: Faurholt-Jepsen et al. 2019(Faurholt-Jepsen et al. 2019b) Associations between daily active/passive self-reported/monitored data and validated measures: Faurholt-Jepsen et al. 2016(Faurholt-Jepsen et al. 2016b)
Faurholt-Jepsen et al. 2020	Daily smartphone self monitoring items—mood, sleep duration, medication taken, activity, irritability, mixed mood, cognitive problems, alcohol consumption, stress, menstruation, individualised EWS, anxiety, self-defined personal parameters, free-text note. Objective smartphone data—phone usage, social activity, step count, GPS location	6 months	Active & Passive	Digital	80.6% adherence to daily self-monitoring in intervention group over 6 months	Total attrition: 35% at 6 months, Intervention group: 22%, Control group: 53%	Associations between active and passive smartphone data (including self-reported mood) and established scales: (Faurholt-Jepsen et al. 2014) Sensitivity, specificity, PPV, NPV and AUC data of objective smartphone data in distinguishing between people with BD and healthy controls: Faurholt-Jepsen et al. 2019(Faurholt-Jepsen et al. 2019b) Associations between daily active/passive self-reported/monitored data and validated measures: Faurholt-Jepsen et al. 2016(Faurholt-Jepsen et al. 2016b) Comparison of sensitivity, specificity and AUC data in activity and mobility data between BD and UD: (Faurholt-Jepsen et al. 2022)
Gliddon et al. 2019	Online mood-monitoring via MoodSwings & MoodSwings-Plus websites	12 months	Active	Digital	Control group: 89% accessed discussion forum, MoodSwings group: 86% accessed the modules, MoodSwings-Plus: 74% accessed the tools	Total attrition: 9% at 12 months, Control group: 6%, MoodSwings group: 7%, MoodSwings-Plus: 13%	Nil validation of this ambulatory assessment protocol – online mood monitoring used as an intervention not as an assessment method
Goldberg et al. 2006	NIMH Life Chart Methodology—daily mood self-rating	6.5 months	Active	Analogue	Not reported	~ 50% attrition at 3 months, > 90% attrition at 6.5 months	Prospective NIMH-Life-Chart Method validation: (Denicoff et al. 2000)
Goulding et al. 2023	Smartphone based self management intervention—daily and weekly check-ins for weeks 1–16. Daily—adherence, sleep, duration, routine, wellness levels. Weekly—symptom severity scoring for all individual DSM-IV mood symptoms	4 months	Active	Digital	The mean (SE) percentage of daily check-ins completed during weeks 1 through 4 was 78% (3%), 74% (3%), 71% (3%), and 64% (3%), respectively, 66% (3%) during week 6, and 47% (4%) during week 16	Intervention group: 15% attrition at 4 months, Control group: 15% attrition at 4 months	Nil validation
Langosch et al. 2008	NIMH Life Chart Methodology—daily mood self-rating	12 months	Active	Analogue	Not reported	55% attrition at 12 months	Prospective NIMH-Life-Chart Method validation: (Denicoff et al. 2000)
Lauder et al. 2015	Online mood-monitoring via MoodSwings & MoodSwings-Plus	12 months	Active	Digital	48% completed all 5 MoodSwings modules, 86.2% completed at least 2 modules, 75.4% completed at least 3 modules	Total attrition: 81% at 12 months, Intervention group: 83%, Control group: 78%	Nil validation of this ambulatory assessment protocol – online mood monitoring used as an intervention not as an assessment method
Leverich et al. 2006	NIMH Life Chart Methodology—daily mood self-rating	12 months	Active	Analogue	Not reported	Not reported	Prospective NIMH-Life-Chart Method validation: (Denicoff et al. 2000)
Lieberman et al. 2010	NIMH Life Chart Methodology—prospective — daily mood self-rating/online Life Chart adaptation	3 months	Active	Analogue vs Digital	Online group rated 44.3 days, standard group rated 20.4 days. Online group entered complete data on 55.2% of days compared to the standard group of 27.7% of days	Total attrition: 46% attrition at 3 months, paper chart: 68% attrition at 3 months, online chart: 22% attrition at 3 months	Prospective NIMH-Life-Chart Method validation: (Denicoff et al. 2000)
Pahwa et al. 2024	KIOS app—self report assessment of 8 different symptoms e.g. sadness/pessimism and delivery of guidance in relation to symptom change eMoods app – self report mood and symptoms diary tracking daily outlook, motivation, habits, sleep, medications etc	53 weeks	Active	Digital	KIOS: 84.4%, eMoods: 54%	KIOS: 12.30%, eMoods: 26.31% at 52 weeks	Nil validation of this ambulatory assessment protocol – online mood monitoring used as an intervention not as an assessment method
Petzold et al. 2019	ChronoRecord—daily mood, sleep, life events, menstrual data, psychiatric medication, weekly—weight	12.5 months	Active	Digital	Not reported	Total: 54.8% over 2 years, Intervention group: 51% attrition over 2 years, Control group: 59% attrition over 2 years	Concurrent validity of analogue ChronoRecord & validated measures of mood and functioning: (Bauer et al. 2004) Concurrent validity of electronic ChronoRecord & validated measures of mood and functioning: (Whybrow et al. 2003) Concurrent validity of electronic ChronoRecord & validated measures of mood and functioning: (Bauer et al. 2008)
van den Berg et al. 2023	NIMH Life Chart Methodology—daily mood and anxiety self-rating	4 months	Active	Digital	Not reported	Total attrition: 18% at 4 months, Intervention group: 16% at 4 months, Control group: 20% at 4 months	Prospective NIMH-Life-Chart Method validation: (Denicoff et al. 2000)

ALS-18 – Affect Lability Score Short Version, AMoSS – Automated Monitoring of Symptoms Severity, ASERT – Aktibipo Self-rating Questionnaire, ASRM – Altman Self-Rated Mania Scale/Altman Self Rating Mania Scale, AUC – Area Under Curve, BAI – Beck Anxiety Inventory, BAS – Behavioural Activation Scale, BDI – Beck Depression Inventory, BD – Bipolar Disorder, BHS – Beck Hopelessness Scale, CGI-BP – Clinical Global Impressions – Bipolar Scale, CI – Confidence Interval, DASS-21 – Depression, Anxiety and Stress Scale 21 item, DSM-5 – Diagnostic and Statistical Manual of Mental Disorders 5th edition, EMA – Ecological Momentary Assessment, FAST – Functional Assessment Short Test, GAD-7 – General Anxiety Disorder-7, GPS – Global Positioning System, GSE – General Self-Efficacy Scale, HAM-D – Hamilton Depression Rating Scale, HC – Healthy Controls, HDRS-17 – Hamilton Depression Rating Scale 17 item, HDRS6–6 item Hamilton Depression Rating Scale, HLOC – Health Locus of Control Scale, IDS – Inventory of Depressive Symptomatology, IDS-C – Inventory of Depressive Symptomatology Clinician Rated, IQR – Inter-Quartile Range, Life-Rift – Level of general functioning and coping: Longitudinal Interval Follow up Evaluation, MARS – Medication Adherence Rating Scale, NIMH – National Institute of Mental Health, NOS – Not Otherwise Specified, PHQ-9 – Patient Health Questionnaire 9 item, PHR-BD – Personal Health Record for Bipolar Disorder, PICS – Perceived Involvement in Care Scales, PLC – Personal Life Chart App, PSWQ – Penn State Worry Questionnaire, PSS – Perceived Stress Scale, QIDS – Quick Inventory of Depressive Symptomatology, QIDS-SR16 – Quick Inventory of Depressive Symptomatology-Self Report-16, RBANS – Repeatable Battery for the Assessment of Neuropsychological Status, ReMAP – Remote Monitoring in Psychiatry, ROC – Receiver Operating Characteristic, RRS – Ruminitive Response Scale, SCID – Structured Clinical Interview for DSM-IV, VAS – Visual Analogue Scale, VSS-A – Verona Satisfaction Scale-Affective Disorder, WHOQoL-BREF – World Health Organisation Quality of Life Scale, YMRS – Young Mania Rating Scale

### Ambulatory assessment protocols of non-randomised studies

25 studies (Anýž et al. 2021; Hidalgo-Mazzei et al. 2016; Garcia-Estela et al. 2022; Bauer et al. 2023; Bos et al. 2022; Bowden et al. 2021a; Dominiak et al. 2022; Emden et al. 2021; Stanislaus et al. 2020; Lee et al. 2022; Born et al. 2014; Lieberman et al. 2011; Kupka et al. 2005; O’Rourke and Sixsmith 2021; Tseng et al. 2022; Ebner-Priemer et al. 2020; Gideon et al. 2016; Schneider et al. 2022; Schärer et al. 2015; Heuvel et al. 2018; Perez Arribas et al. 2018; Lewis et al. 2023; McKnight et al. 2017; Ortiz et al. 2023) used 21 different ambulatory assessment/mood tracking procedures. These were: ASERT (Anýž et al. 2021), SIMPLe (Hidalgo-Mazzei et al. 2016, 2018; Garcia-Estela et al. 2022), ChronoRecord (Bauer et al. 2023), RoQua (Bos et al. 2022), KIOS (Bowden et al. 2021b), BDmon (Dominiak et al. 2022), ReMAP (Emden et al. 2021), Monsenso (Stanislaus et al. 2020), eMoodChart (Lee et al. 2022), NIMH Life Chart Methodology – prospective (Born et al. 2014; Kupka et al. 2005), Social Rhythm Metric (Lieberman et al. 2011), Bipolar Disorder Symptom Scale (O’Rourke and Sixsmith 2021), unspecified ambulatory assessment smartphone application (Tseng et al. 2022), MovisensXS (Ebner-Priemer et al. 2020), PRIORI (Gideon et al. 2016), MINDPAX (Schneider et al. 2022), PLC (Schärer et al. 2015), PHR-BD (Heuvel et al. 2018), AMoSS (Perez Arribas et al. 2018), True Colours (Lewis et al. 2023; McKnight et al. 2017) and e-monitoring system (Ortiz et al. 2023).

17 of these ambulatory assessment procedures involved daily charting (SIMPLe (Hidalgo-Mazzei et al. 2016, 2018; Garcia-Estela et al. 2022), ChronoRecord (Bauer et al. 2023), RoQua (Bos et al. 2022), BDmon (Dominiak et al. 2022), ReMAP (Emden et al. 2021), Monsesnso (Stanislaus et al. 2020), eMoodChart (Lee et al. 2022), NIMH Life Chart Methodology (Born et al. 2014; Kupka et al. 2005), MoodChart (Lee et al. 2022), Bipolar Disorder Symptom Scale (O’Rourke and Sixsmith 2021), unspecified smartphone application (Tseng et al. 2022), MovisensXS (Ebner-Priemer et al. 2020), PRIORI (Gideon et al. 2016), MINDPAX (Schneider et al. 2022), PLC (Schärer et al. 2015), PHR-BD (Heuvel et al. 2018), AMoSS (Perez Arribas et al. 2018)) while 3 involved weekly charting (ASERT (Anýž et al. 2021), KIOS (Bowden et al. 2021b), True Colours (Lewis et al. 2023; McKnight et al. 2017)). Passive ambulatory assessment was used by: BDmon (Dominiak et al. 2022), ReMAP (Emden et al. 2021), Monsenso (Stanislaus et al. 2020), eMoodChart (Lee et al. 2022), Bipolar Disorder Symptom Scale (O’Rourke and Sixsmith 2021), unspecified ambulatory assessment smartphone application (Tseng et al. 2022), MovisensXS (Ebner-Priemer et al. 2020), PRIORI (Gideon et al. 2016), MINDPAX (Schneider et al. 2022), AMoSS (Perez Arribas et al. 2018), e-monitoring system (Ortiz et al. 2023) all of which combined active and passive ambulatory assessment apart from MINDPAX (Schneider et al. 2022) which just used passive ambulatory assessment via actigraphy. ASERT (Anýž et al. 2021), SIMPLe (Hidalgo-Mazzei et al. 2016, 2018; Garcia-Estela et al. 2022), ChronoRecord (Bauer et al. 2023), RoQua (Bos et al. 2022), KIOS (Bowden et al. 2021b), NIMH Life Chart Methodology (Born et al. 2014; Kupka et al. 2005), Social Rhythm Metric (Lieberman et al. 2011), PLC (Schärer et al. 2015), PHR-BD (Heuvel et al. 2018), True Colours (Lewis et al. 2023; McKnight et al. 2017) used active ambulatory assessment alone.

Only the NIMH Life Chart studies (Born et al. 2014; Kupka et al. 2005) used analogue methods of assessment with the rest using digital methods. The follow up of included studies ranged from 3 months (Hidalgo-Mazzei et al. 2016; Lieberman et al. 2011; Schneider et al. 2022; Perez Arribas et al. 2018; Bowden et al. 2021b) to 2 + years (McKnight et al. 2017).

### Ambulatory assessment protocols of randomised studies

17 trials (Bilderbeck et al. 2016; Denicoff et al. 2002; Faurholt-Jepsen et al. 2015, 2020, 2021; Lauder et al. 2015; Gliddon et al. 2019; Castle et al. 2010; Goulding et al. 2023; Petzold et al. 2019; Berg et al. 2023; Goldberg et al. 2008; Langosch et al. 2008; Lieberman et al. 2010; Depp et al. 2012; Leverich et al. 2006; Pahwa et al. 2024) used 9 different ambulatory assessment/mood tracking procedures. These were: TrueColours (Bilderbeck et al. 2016), NIMH Life Chart Methodology – prospective (we only included prospective life chart studies rather than retrospective ones (Leverich et al. 2006; Ellison et al. 2020)), the MONARCA/Monsenseo system, Livewell, MoodSwings (Lauder et al. 2015; Gliddon et al. 2019), telephone calls (Castle et al. 2010), ChronoRecord (Petzold et al. 2019), KIOS (Pahwa et al. 2024) and eMoods (Pahwa et al. 2024). Only the NIMH Life Chart studies used ambulatory assessment as their primary outcome, with the others using more standardized infrequent assessments of mood e.g. MADRS, YMRS. Most of these ambulatory assessment procedures involved daily charting (NIMH LC, MONARCA/Monsenseo/Livewell/ChronoRecord, MoodSwings, KIOS, eMoods), apart from TrueColours and Castle et al. 2010 which utilized weekly assessments.

Only the MONARCA/Monsenseo system explored the impact of passive data capture in a formal randomized trial where the system was deployed as an intervention with clinical monitoring and subsequent feedback to the participant. This is obviously different to using passive monitoring as a method of assessment, despite validation of the measures. The NIMH life chart method and telephone calls in Castle et al. 2010 were the only analogue methods examined. The NIMH life chart method was also compared against a digital version. The follow up of included studies ranged from 3 months (Berg et al. 2023) to 3 years (Denicoff et al. 2002).

### Risk of bias assessments

The methodological quality of these non-randomised studies was variable but most were considered of low-moderate risk of bias (Supplementary Table 4).

### Performance

We included 462 correlation coefficients and 25 confusion matrices (Supplementary Tables 2 & 3). 318 of these statistics compared active ambulatory assessment vs established measures, 74 compared passive ambulatory assessment vs another type of passive ambulatory assessment, 66 compared active ambulatory assessment with passive ambulatory assessment, 23 compared active ambulatory assessment via another type of active ambulatory assessment and 6 compared passive ambulatory assessment with established measures. There were 487 different smartphone-based measures of mood, functioning, sleep, quality of life, diagnosis and physical activity (Supplementary Tables 2 & 3). A robust evaluation of performance was not possible as no study measured the same parameters in a similar enough way to be able to conduct a meaningful meta-analysis. 11 out of 42 studies did not report a validation study.

Correlation coefficients ranged from −0.84 to 0.97. The mean correlation coefficient for active ambulatory assessment vs established measures was 0.22 (SD: 0.38, range −0.84 to −0.97), for passive ambulatory assessment measures vs established measures it was −0.07 (SD: 0.10, range: −0.21 to 0.05). For active ambulatory assessment vs established measures there were 33 strong, 127 moderate and 147 weak correlation coefficients. For passive ambulatory assessment versus established measures there were 6 weak correlation coefficients. For active ambulatory assessment measures vs passive ambulatory assessment measures there was 1 strong, 21 moderate and 42 weak correlation coefficients.

The active ambulatory assessment items that performed well were often smartphone delivered self-report questionnaires that were similar to the established measure (e.g. ‘REMAP BDI vs non-smartphone based BDI’), or single item mood questions e.g. ‘NIMH Life chart method’. None of the passive ambulatory assessment items had good performance when compared to existing measures and there was a paucity of data for this variable. The only passive ambulatory assessment item that had good performance when compared with active ambulatory assessment was ‘Week-to-week repeat measures correlations between Total daily distance of movement Time 1/Total daily distance of movement Time 2’ (Tseng et al. 2022) – although it is possible that this item could also have poor performance when compared to an established measure. These two measures may have correlated well as they are both measuring the same construct – daily distance, or alternatively differences in daily distance between two time points may actually be a better measure of mood than comparing daily distance versus sleep duration or self-reported mood (which had weak correlations). As the daily mood measure used a combination of the ASRM, YMRS, HAM-D and DAS-21 – all of which are established measures of mood – it is surprising that this did not correlate with daily distance – and suggests that the moderate strength of the correlation reported here between the two time points of the distance measure is more related to measurement similarity rather than true performance. The study does not specify if these were pre-specified analyses or post-hoc observations but as the study only analysed three metrics (daily distance, sleep duration, daily mood) it is reasonable to assume these analyses were pre-specified.

## Discussion

This systematic review highlights large heterogeneity in ambulatory assessment protocols for bipolar disorder which prevents substantial conclusions on their performance and comparative utility. We also highlight the paucity of validation studies investigating passive ambulatory assessment for people with bipolar disorder, particularly when compared to ambulatory assessment protocols in depression which appear more advanced (Wright 2025a). Despite the promise of ambulatory assessment in providing less subjective behavioural mood measurement methods (via things such as phone usage, GPS location, heart rate) the evidence summarised here lacks sufficient methodological consistency to support its widespread implementation in people with bipolar disorder. More consistency in the measurement and reporting of these statistics is necessary to understand these measures and replicate their findings robustly.

This is the first systematic review of the performance of active and passive ambulatory assessment in people with bipolar disorder. Despite extracting 462 correlation coefficients from validation studies, our primary finding was that it was not possible to aggregate performance data in a robust and meaningful way. This was because of the substantial heterogeneity in measures, which reported a wide variety of correlation coefficients (e.g., Spearman’s, Pearson’s, weighted group level correlation coefficient, intraclass correlation coefficient etc.) and were derived from diverse data types (e.g., GPS location, SMS logs, call logs, heart rate) collected over wide ranging time periods (weekly, daily, or continuous) and in substantially different populations (community, inpatient environments) drawn from different countries. Some studies reported aggregate scores, while others relied on individual items. A consensus framework for evaluating ambulatory assessment performance—with standardised timepoints, outcome metrics, and analysis methods—would support more rigorous comparisons. The establishment of core outcome sets would also facilitate cross-study and cross-population comparability. Currently, there are no established mental health guidelines for reporting ambulatory assessment performance, and developing these standards would require consensus among experts on design, methodology and reporting e.g. statistical considerations. Existing guidelines do not address this key gap (Research WO 2025). In our view, validation of digital measures should be included within the main trial or cohort publication. Without this, the reliability of the findings cannot be confirmed, and it remains impossible to pool results in a meta-analysis to draw broader conclusions about ambulatory assessment performance. Furthermore, our results raise further questions around the appropriateness of validating new measures against established self-report measures which themselves have known flaws (Guo et al. 2017).

Despite not conducting a meta-analysis, we did observe some patterns in the overall data. The vast majority of validation work was assessing active ambulatory assessment against established measures and yet the results for this were mixed with 48% of these demonstrating weak performance. There were very few comparisons of passive ambulatory assessment, particularly against established self-report measures. Passive ambulatory assessment is poorly developed and validated against established measures of mood. We were not able to determine if single item passive digital measures e.g. heart rate performed less well than aggregate measures (e.g. incorporating multiple data types across multiple sensors). Single item measures of mood or simply digital versions of established self-report scales generally had the best performance against established mood measures and these appeared ready for clinical/research utilisation, but the vast majority of measures did not demonstrate sufficient performance for established use.

The central issue of whether these advanced digital measures have good performance in measuring mood or not remains unsolved. Answering this question is critical for subsequent implementation in research and clinical services as good performance will lead to trust in a metric that then allows people with BD and clinicians to make key decisions for their illness and recovery planning as well as accurately measure mood in research (Wright et al. 2025b). How to ensure ambulatory assessment is clinically useful/useful for research/implemented well alongside people with BD is explored extensively in our other research underpinning this review (Wright et al. 2025b) which highlights the importance of performance – which does not have to be perfect, but sufficiently right to create trust in the metric and promote insight or to be comparable to existing research measures (Wright et al. 2024). Advances in technology have enabled the tracking of complex behaviour such as step count and voice, creating a rapidly evolving field that often relies on sophisticated statistical methods to evaluate these tools. Unfortunately, many of the validation studies were not published in the primary trial or cohort reports, making them difficult to locate—often necessitating direct communication with study authors to obtain the necessary data. Many of the studies we included in this review did not report any validation of their measures. It is recommended that researchers validate any new or untested measures against established self-report measures to benchmark these. There are known limitations to self-report measures and standardised clinical assessment may be a more reliable gold standard. Furthermore, people with BD want multiple methods of assessing and tracking their symptoms and so a diversity of measures is important (Wright 2024). Using both active and passive ambulatory assessment approaches in parallel may also help to better evaluate and compare the performance of each method.

This review incorporates studies which use ambulatory assessment in a wide variety of ways, some of which may affect the performance of the ambulatory assessment – although performance was sometimes (but not always) assessed in smaller separate studies that often differed from the main study in important ways – e.g. smaller follow up periods and in the case of ambulatory assessment used as a research outcome – excluding any additional interventional elements. Thus there are multiple factors which may impact the performance of the ambulatory assessment protocol e.g. adherence, attrition, adverse events, user experience, effectiveness of ambulatory assessment in changing mood, all of which are also important to consider and because of the wealth of data for each of these factors we have published them as separate reviews (Wright et al. 2025b, 2025c; Aw 2025a, 2025b). All of these factors affect clinical/research utility and must be considered in depth individually. It is important to note that while this review focusses on performance, these other factors (e.g. user experience, or improve adherence/attrition) may affect and compensate for sub-optimal psychometric properties. We hoped to assess some of these factors quantitatively via sub-group analysis to assess if these affected performance but unfortunately this was not possible due to the aforementioned limitations in the data, but was possible in some of the reviews highlighted above. These questions remain crucial for future research and implementation of these protocols.

We found 476 different smartphone-based measures although there was a degree of overlap between some measures. The vast majority of these were evaluating active ambulatory assessment measures which are generally much less complicated and nuanced measures that require less statistical processing to use. Many more measures reported non-correlation coefficient statistics that we did not report here. This paper highlights the key questions that remain in the field of ambulatory assessment and bipolar disorder – how passive ambulatory assessment measures will perform against established measures, and how to optimally combine these numerous smartphone-based measures of activity and mobility available to accurately predict mood on both an individual and population level. Ensuring these measures have clinical meaning should be a major focus for future research. It is likely that some passive measures will be much better indicators of either depression or mania and there is likely to be a wide variability in this performance. Some of this is observed in Supplementary Table 2. The research required to answer some of these questions will likely involve large sample sizes, which are necessary to capture the large variability in human behaviour both in normal and abnormal mental functioning (Torous et al. 2020; Matcham et al. 2019). Performance is key for future development and once singular items are discovered with high degrees of performance, or combined with other metrics to produce good performance, this can be translated into meaningful clinically relevant metrics to use as a treatment outcome in RCTs or as novel mood-monitoring based interventions, for example.

This paper focused on the performance of active ambulatory assessment measures and these require certain considerations when evaluating them. As they are isolated measurements rather than continuous observations (like, for example, heart rate variability over one week) they do not take into account real time variability. For example it is important to acknowledge the diurnal variation in bipolar disorder (e.g. bipolar depression can display positive mood variation – worse mood on waking with improved mood in the evening) (Carr et al. 2018) and thus ambulatory assessment should ideally be administered at the same time each day.

This paper has several strengths and limitations. We focused on studies that employed ambulatory assessment at least weekly over a minimum duration of three months. Although the choice of a three-month follow-up is somewhat arbitrary, we believe it is necessary to evaluate mood patterns and usability in a way that is clinically meaningful. Numerous studies explored both active and passive ambulatory assessment validation through cross-sectional designs with shorter follow-up periods, but these were excluded as they fall outside the scope of this review and are less applicable to real-world clinical practice. By selecting studies with follow-up periods relevant to clinical settings, we aimed to draw conclusions about ambulatory assessment approaches that could realistically impact current mental health trial methodologies. Our search strategy followed Cochrane guidelines and was comprehensive, but the variability in terminology used by authors means that some relevant studies may have been overlooked. Nonetheless, we believe the number of missed studies is minimal, as we also conducted reference checks, consulted field experts, and used a broad search strategy that likely captured more relevant literature than prior reviews (Dubad et al. 2018; Watt et al. 2020; Ameringen et al. 2017). Significant heterogeneity was observed across studies in how ambulatory assessment was applied, the interventions used, and the basis for validation or association, which limited our ability to synthesize performance outcomes through meta-analysis. Still, this variability is itself a valuable finding, highlighting the need for greater consistency in future research to enhance clinical relevance. Additionally, most studies faced risk of bias issues related to lack of blinding in ambulatory assessment or intervention procedures—an ongoing challenge in psychological research where full blinding is often not feasible. Furthermore, many of the included studies reported high attrition/low adherence rates and we discuss this comprehensively elsewhere in a separate paper of similar studies currently under review (Aw 2025a). As some of the validation studies quantifying performance were separate studies there are limits to how much the concerning attrition/adherence rates affected the performance metrics documented here.

This review highlights the need for greater standardisation in ambulatory assessment methodologies to enable widespread implementation of these emerging behavioural measures. Although ambulatory assessment offers a more adaptable and efficient approach to monitoring mood and mental states, current protocols are often poorly developed, validated and show marked inconsistency in methods and outcome reporting. The wide variation in ambulatory assessment protocols across studies makes it difficult to compare performance reliably, which hinders the practical application of these tools in bipolar disorder. At present, it remains unclear whether passive ambulatory assessment truly offers a less subjective approach to mood assessment. We therefore call for improved standardisation in how performance is measured and reported. Future studies should focus on developing clear reporting frameworks and standardised protocols, which would facilitate the replication of results and enable more robust comparisons—ultimately advancing the use of ambulatory assessment as a valuable research tool in bipolar disorder.

## Supplementary Information

Additional file 1.

## Data Availability

No datasets were generated or analysed during the current study.

## References

[CR1] aan het Rot M, Hogenelst K, Schoevers RA. Mood disorders in everyday life: a systematic review of experience sampling and ecological momentary assessment studies. Clin Psychol Rev. 2012;32(6):510–23. 22721999 10.1016/j.cpr.2012.05.007

[CR2] Altman EG, Hedeker D, Peterson JL, Davis JM. The altman self-rating mania scale. Biol Psychiatry. 1997;42(10):948–55. 9359982 10.1016/S0006-3223(96)00548-3

[CR3] Van Ameringen M, Turna J, Khalesi Z, Pullia K, Patterson B. There is an app for that! The current state of mobile applications (apps) for DSM-5 obsessive-compulsive disorder, posttraumatic stress disorder, anxiety and mood disorders. Depress Anxiety. 2017;34(6):526–39. 28569409 10.1002/da.22657

[CR4] Antosik-Wojcinska AZ, Dominiak M, Chojnacka M, Kaczmarek-Majer K, Opara KR, Radziszewska W, et al. Smartphone as a monitoring tool for bipolar disorder: a systematic review including data analysis, machine learning algorithms and predictive modelling. Int J Med Inform. 2020;138. 10.1016/j.ijmedinf.2020.104131 32305023

[CR5] Anýž J, Bakštein E, Dally A, Kolenič M, Hlinka J, Hartmannová T, et al. Validity of the aktibipo self-rating questionnaire for the digital self-assessment of mood and relapse detection in patients with bipolar disorder: instrument validation study. JMIR Ment Health. 2021. 10.2196/26348. 34383689 10.2196/26348 PMC8386400

[CR6] L AW. Dropout, attrition, adherence and compliance in mood monitoring studies and ambulatory assessment studies in depression and bipolar disorder: a systematic review and meta-analysis. 2025a. 10.2196/83765 PMC12795494 41525703

[CR7] L AW. Mood monitoring and mood tracking interventions in depression and bipolar disorder: a systematic review and meta-analysis of randomized controlled trials. 2025b. 10.2196/84020 PMC12779106 41499681

[CR8] Bauer M, Grof P, Gyulai L, Rasgon N, Glenn T, Whybrow PC. Using technology to improve longitudinal studies: self-reporting with ChronoRecord in bipolar disorder. Bipolar Disord. 2004;6(1):67–74. 14996143 10.1046/j.1399-5618.2003.00085.x

[CR9] Bauer M, Wilson T, Neuhaus K, Sasse J, Pfennig A, Lewitzka U, et al. Self-reporting software for bipolar disorder: validation of ChronoRecord by patients with mania. Psychiatry Res. 2008;159(3):359–66. 18423616 10.1016/j.psychres.2007.04.013

[CR10] Bauer M, Glenn T, Alda M, Grof P, Bauer R, Ebner-Priemer UW, et al. Longitudinal digital mood charting in bipolar disorder: experiences with chronorecord over 20 years. Pharmacopsychiatry. 2023;56(5):182–7. 37678394 10.1055/a-2156-5667 PMC10484643

[CR11] Bowden CL, Priesmeyer R, Tohen M, Singh V, Calabrese JR, Ketter T, Nierenberg A, Thase ME, Siegel G, Siegel LH, Mintz J, El-Mallakh RS, McElroy SL, Martinez M. Development of a Patient-Centered Software System to Facilitate Effective Management of Bipolar Disorder. Psychopharmacol Bull. 2021 Mar 16;51(2):8-19. 10.64719/pb.4404. 10.64719/pb.4404 PMC8146566 34092819

[CR12] Bilderbeck AC, Atkinson LZ, McMahon HC, Voysey M, Simon J, Price J, et al. Psychoeducation and online mood tracking for patients with bipolar disorder: a randomised controlled trial. J Affect Disord. 2016;205:245–51. 27454410 10.1016/j.jad.2016.06.064

[CR13] Born C, Amann BL, Grunze H, Post RM, Schärer L. Saving time and money: a validation of the self ratings on the prospective NIMH life-chart method (NIMH-LCM). BMC Psychiatry. 2014;14:130. 24886463 10.1186/1471-244X-14-130 PMC4031162

[CR14] Bos FM, Schreuder MJ, George SV, Doornbos B, Bruggeman R, van der Krieke L, et al. Anticipating manic and depressive transitions in patients with bipolar disorder using early warning signals. Int J Bipolar Disord. 2022. 10.1186/s40345-022-00258-4. 35397076 10.1186/s40345-022-00258-4 PMC8994809

[CR15] Bowden CL, Priesmeyer R, Tohen M, Singh V, Calabrese JR, Ketter T, et al. Development of a patient-centered software system to facilitate effective management of bipolar disorder. Psychopharmacol Bull. 2021;51(2):8–19. 34092819 10.64719/pb.4404 PMC8146566

[CR16] Brietzke E, Hawken ER, Idzikowski M, Pong J, Kennedy SH, Soares CN. Integrating digital phenotyping in clinical characterization of individuals with mood disorders. Neurosci Biobehav Rev. 2019;104:223–30. 31330197 10.1016/j.neubiorev.2019.07.009

[CR17] Carr O, Saunders KEA, Tsanas A, Bilderbeck AC, Palmius N, Geddes JR, et al. Variability in phase and amplitude of diurnal rhythms is related to variation of mood in bipolar and borderline personality disorder. Sci Rep. 2018;8(1):1649. 29374207 10.1038/s41598-018-19888-9 PMC5786095

[CR18] Castle D, White C, Chamberlain J, Berk M, Berk L, Lauder S, et al. Group-based psychosocial intervention for bipolar disorder: randomised controlled trial. Br J Psychiatry. 2010;196(5):383–8. 20435965 10.1192/bjp.bp.108.058263

[CR19] Denicoff KD, Leverich GS, Nolen WA, Rush AJ, McElroy SL, Keck PE, et al. Validation of the prospective NIMH-Life-Chart Method (NIMH-LCM-p) for longitudinal assessment of bipolar illness. Psychol Med. 2000;30(6):1391–7. 11097079 10.1017/s0033291799002810

[CR20] Denicoff KD, Ali SO, Sollinger AB, Smith-Jackson EE, Leverich GS, Post RM. Utility of the daily prospective National Institute of Mental Health Life-Chart Method (NIMH-LCM-p) ratings in clinical trials of bipolar disorder. Depress Anxiety. 2002;15(1):1–9. 11816046 10.1002/da.1078

[CR21] Depp CA, Kim DH, de Dios LV, Wang V, Ceglowski J. A pilot study of mood ratings captured by mobile phone versus paper-and-pencil mood charts in bipolar disorder. J Dual Diagn. 2012;8(4):326–32. 23646035 10.1080/15504263.2012.723318 PMC3640573

[CR22] Dominiak M, Kaczmarek-Majer K, Antosik-Wojcinska AZ, Opara KR, Olwert A, Radziszewska W, et al. Behavioral and self-reported data collected from smartphones for the assessment of depressive and manic symptoms in patients with bipolar disorder: prospective observational study. J Med Internet Res. 2022;24(1):e28647. 34874015 10.2196/28647 PMC8811705

[CR23] Dubad M, Winsper C, Meyer C, Livanou M, Marwaha S. A systematic review of the psychometric properties, usability and clinical impacts of mobile mood-monitoring applications in young people. Psychol Med. 2018;48(2):208–28. 28641609 10.1017/S0033291717001659

[CR24] Ebner-Priemer UW, Mühlbauer E, Neubauer AB, Hill H, Beier F, Santangelo PS, et al. Digital phenotyping: towards replicable findings with comprehensive assessments and integrative models in bipolar disorders. Int J Bipolar Disord. 2020;8(1):35. 33211262 10.1186/s40345-020-00210-4 PMC7677415

[CR25] Ellison WD, Trahan AC, Pinzon JC, Gillespie ME, Simmons LM, King KY. For whom, and for what, is experience sampling more accurate than retrospective report? Pers Individ Dif. 2020;163:110071.

[CR26] Emden D, Goltermann J, Dannlowski U, Hahn T, Opel N. Technical feasibility and adherence of the remote monitoring application in psychiatry (ReMAP) for the assessment of affective symptoms. J Affect Disord. 2021;294:652–60. 34333173 10.1016/j.jad.2021.07.030

[CR27] Excellence NIfHaC. BIPOLAR DISORDER (UPDATE) REVIEW PROTOCOLS. 2021.

[CR28] Faurholt-Jepsen M, Frost M, Ritz C, Christensen E, Jacoby A, Mikkelsen R, et al. Daily electronic self-monitoring in bipolar disorder using smartphones-the MONARCA I trial: a randomized, placebo-controlled, single-blind, parallel group trial. Psychol Med. 2015;45(13):2691–704. 26220802 10.1017/S0033291715000410

[CR29] Faurholt-Jepsen M, Munkholm K, Frost M, Bardram JE, Kessing LV. Electronic self-monitoring of mood using IT platforms in adult patients with bipolar disorder: a systematic review of the validity and evidence. BMC Psychiatry. 2016a;16(1):7. 26769120 10.1186/s12888-016-0713-0 PMC4714425

[CR30] Faurholt-Jepsen M, Vinberg M, Frost M, Debel S, Christensen EM, Bardram JE, et al. Behavioral activities collected through smartphones and the association with illness activity in bipolar disorder. Int J Methods Psychiatr Res. 2016b;25(4):309–23. 27038019 10.1002/mpr.1502 PMC6860202

[CR31] Faurholt-Jepsen M, Geddes JR, Goodwin GM, Bauer M, Duffy A, Vedel Kessing L, et al. Reporting guidelines on remotely collected electronic mood data in mood disorder (eMOOD)-recommendations. Transl Psychiatry. 2019a. 10.1038/s41398-019-0484-8. (**(no pagination)**). 31175283 10.1038/s41398-019-0484-8 PMC6555812

[CR32] Faurholt-Jepsen M, Busk J, Þórarinsdóttir H, Frost M, Bardram JE, Vinberg M, et al. Objective smartphone data as a potential diagnostic marker of bipolar disorder. Aust N Z J Psychiatry. 2019b;53(2):119–28. 30387368 10.1177/0004867418808900

[CR33] Faurholt-Jepsen M, Frost M, Christensen EM, Bardram JE, Vinberg M, Kessing LV. The effect of smartphone-based monitoring on illness activity in bipolar disorder: the MONARCA II randomized controlled single-blinded trial. Psychol Med. 2020;50(5):838–48. 30944054 10.1017/S0033291719000710

[CR34] Faurholt-Jepsen M, Frost M, Vinberg M, Christensen EM, Bardram JE, Kessing LV. Smartphone data as objective measures of bipolar disorder symptoms. Psychiatry Res. 2014 Jun 30;217(1-2):124-7. 10.1016/j.psychres.2014.03.009. 10.1016/j.psychres.2014.03.009 24679993

[CR35] Faurholt-Jepsen M, Lindbjerg Tønning M, Fros M, Martiny K, Tuxen N, Rosenberg N, et al. Reducing the rate of psychiatric re-admissions in bipolar disorder using smartphones-the RADMIS trial. Acta Psychiatr Scand. 2021;143(5):453–65. 33354769 10.1111/acps.13274

[CR36] Faurholt-Jepsen M, Busk J, Rohani DA, Frost M, Tonning ML, Bardram JE, et al. Differences in mobility patterns according to machine learning models in patients with bipolar disorder and patients with unipolar disorder. J Affect Disord. 2022;306:246–53. 35339568 10.1016/j.jad.2022.03.054

[CR37] Faurholt-Jepsen M, Vinberg M, Frost M, Debel S, Margrethe Christensen E, Bardram JE, Kessing LV. Behavioral activities collected through smartphones and the association with illness activity in bipolar disorder. Int J Methods Psychiatr Res. 2016 Dec;25(4):309-323. 10.1002/mpr.1502. 10.1002/mpr.1502 PMC6860202 27038019

[CR38] Garcia-Estela A, Cantillo J, Angarita-Osorio N, Mur-Mila E, Anmella G, Perez V, et al. Real-world implementation of a smartphone-based psychoeducation program for bipolar disorder: observational ecological study. J Med Internet Res. 2022;24(2):e31565. 35107440 10.2196/31565 PMC8851334

[CR39] Gideon J, Provost EM, McInnis M. Mood state prediction from speech of varying acoustic quality for individuals with bipolar disorder. Proc IEEE Int Conf Acoust Speech Signal Process. 2016;2016:2359-63. 10.1109/ICASSP.2016.7472099 PMC4995442 27570493

[CR41] Gliddon E, Cosgrove V, Berk L, Lauder S, Mohebbi M, Grimm D, Dodd S, Coulson C, Raju K, Suppes T, Berk M. A randomized controlled trial of MoodSwings 2.0: An internet-based self-management program for bipolar disorder. Bipolar Disord. 2019 Feb;21(1):28-39. 10.1111/bdi.12669. 10.1111/bdi.12669 29931798

[CR42] Goldberg JF, Bowden CL, Calabrese JR, Ketter TA, Dann RS, Frye MA, et al. Six-month prospective life charting of mood symptoms with lamotrigine monotherapy versus placebo in rapid cycling bipolar disorder. Biol Psychiatry. 2008;63(1):125–30. 17543894 10.1016/j.biopsych.2006.12.031

[CR43] Goldberg JF, Bowden CL, Calabrese JR, Ketter TA, Dann RS, Frye MA, Suppes T, Post RM. Six-month prospective life charting of mood symptoms with lamotrigine monotherapy versus placebo in rapid cycling bipolar disorder. Biol Psychiatry. 2008 Jan 1;63(1):125-30. 10.1016/j.biopsych.2006.12.031. 10.1016/j.biopsych.2006.12.031 17543894

[CR44] Goltermann J, Emden D, Leehr EJ, Dohm K, Redlich R, Dannlowski U, et al. Smartphone-based self-reports of depressive symptoms using the Remote Monitoring Application in Psychiatry (ReMAP): interformat validation study. JMIR Ment Health. 2021;8(1):e24333. 33433392 10.2196/24333 PMC7837996

[CR46] Goulding EH, Dopke CA, Rossom R, Jonathan G, Mohr D, Kwasny MJ. Effects of a Smartphone-Based Self-management Intervention for Individuals With Bipolar Disorder on Relapse, Symptom Burden, and Quality of Life: A Randomized Clinical Trial. JAMA Psychiatry. 2023 Feb 1;80(2):109-118. doi: 10.1001/jamapsychiatry.2022.4304. 10.1001/jamapsychiatry.2022.4304 PMC9857325 36542401

[CR47] Grunze H, Born C. The impact of subsyndromal bipolar symptoms on patient’s functionality and quality of life. Front Psychiatry. 2020;11:510. 32595531 10.3389/fpsyt.2020.00510 PMC7304232

[CR48] Guo B, Kaylor-Hughes C, Garland A, Nixon N, Sweeney T, Simpson S, et al. Factor structure and longitudinal measurement invariance of PHQ-9 for specialist mental health care patients with persistent major depressive disorder: exploratory structural equation modelling. J Affect Disord. 2017;219:1–8. 28501679 10.1016/j.jad.2017.05.020 PMC6602881

[CR49] van den Heuvel SC, Meije D, Regeer EJ, Sinnema H, Riemersma RF, Kupka RW. The user experiences and clinical outcomes of an online personal health record to support self-management of bipolar disorder: a pretest-posttest pilot study. J Affect Disord. 2018;238:261–8. 29894931 10.1016/j.jad.2018.05.069

[CR50] Hidalgo-Mazzei D, Mateu A, Reinares M, Murru A, del Mar Bonnin C, Varo C, et al. Psychoeducation in bipolar disorder with a SIMPLe smartphone application: feasibility, acceptability and satisfaction. J Affect Disord. 2016;200:58–66. 27128358 10.1016/j.jad.2016.04.042

[CR51] Hidalgo-Mazzei D, Reinares M, Mateu A, Nikolova VL, Bonnín CDM, Samalin L, et al. OpenSIMPLe: a real-world implementation feasibility study of a smartphone-based psychoeducation programme for bipolar disorder. J Affect Disord. 2018;241:436–45. 30145515 10.1016/j.jad.2018.08.048

[CR52] Koenders MA, Nolen WA, Giltay EJ, Hoencamp E, Spijker AT. The use of the prospective NIMH life chart method as a bipolar mood assessment method in research: a systematic review of different methods, outcome measures and interpretations. J Affect Disord. 2015;175:260–8. 25658502 10.1016/j.jad.2015.01.005

[CR53] Kupka RW, Luckenbaugh DA, Post RM, Suppes T, Altshuler LL, Keck PE Jr., et al. Comparison of rapid-cycling and non-rapid-cycling bipolar disorder based on prospective mood ratings in 539 outpatients. Am J Psychiatry. 2005;162(7):1273–80. 15994709 10.1176/appi.ajp.162.7.1273

[CR54] Langosch JM, Drieling T, Biedermann NC, Born C, Sasse J, Bauer H, et al. Efficacy of quetiapine monotherapy in rapid-cycling bipolar disorder in comparison with sodium valproate. J Clin Psychopharmacol. 2008;28(5):555–60. 18794653 10.1097/JCP.0b013e318185e75f

[CR55] Lauder S, Chester A, Castle D, Dodd S, Gliddon E, Berk L, et al. A randomized head to head trial of MoodSwings.net.au: an Internet based self-help program for bipolar disorder. J Affect Disord. 2015;171:13–21. 25282145 10.1016/j.jad.2014.08.008

[CR56] Lee HJ, Cho CH, Lee T, Jeong J, Yeom JW, Kim S, et al. Prediction of impending mood episode recurrence using real-time digital phenotypes in major depression and bipolar disorders in South Korea: a prospective nationwide cohort study. Psychol Med. 2022:1–9. 10.1017/S0033291722002847 36146953

[CR57] Leverich GS, Altshuler LL, Frye MA, Suppes T, McElroy SL, Keck PE Jr., et al. Risk of switch in mood polarity to hypomania or mania in patients with bipolar depression during acute and continuation trials of venlafaxine, sertraline, and bupropion as adjuncts to mood stabilizers. Am J Psychiatry. 2006;163(2):232–9. 16449476 10.1176/appi.ajp.163.2.232

[CR58] Lewis KJS, Tilling K, Gordon-Smith K, Saunders KEA, Di Florio A, Jones L, et al. The dynamic interplay between sleep and mood: an intensive longitudinal study of individuals with bipolar disorder. Psychol Med. 2023;53(8):3345–54. 35074035 10.1017/S0033291721005377 PMC10277721

[CR59] Lieberman DZ, Kelly TF, Douglas L, Goodwin FK. A randomized comparison of online and paper mood charts for people with bipolar disorder. J Affect Disord. 2010;124(1–2):85–9. 19896202 10.1016/j.jad.2009.10.019

[CR60] Lieberman DZ, Swayze S, Goodwin FK. An automated Internet application to help patients with bipolar disorder track social rhythm stabilization. Psychiatr Serv. 2011;62(11):1267–9. 22211203 10.1176/ps.62.11.pss6211_1267

[CR61] Lublóy Á, Keresztúri JL, Németh A, Mihalicza P. Exploring factors of diagnostic delay for patients with bipolar disorder: a population-based cohort study. BMC Psychiatry. 2020;20(1):75. 32075625 10.1186/s12888-020-2483-y PMC7031950

[CR62] Malhi GS, Hamilton A, Morris G, Mannie Z, Das P, Outhred T. The promise of digital mood tracking technologies: are we heading on the right track? Evid Based Ment Health. 2017;20(4):102–7. 28855245 10.1136/eb-2017-102757 PMC10516397

[CR63] Matcham F, di San B, Pietro C, Bulgari V, de Girolamo G, Dobson R, et al. Remote assessment of disease and relapse in major depressive disorder (RADAR-MDD): a multi-centre prospective cohort study protocol. BMC Psychiatry. 2019;19:72. 30777041 10.1186/s12888-019-2049-z PMC6379954

[CR64] McIntyre RS, Berk M, Brietzke E, Goldstein BI, López-Jaramillo C, Kessing LV, et al. Bipolar disorders. Lancet. 2020;396(10265):1841–56. 33278937 10.1016/S0140-6736(20)31544-0

[CR65] McKnight RF, Bilderbeck AC, Miklowitz DJ, Hinds C, Goodwin GM, Geddes JR. Longitudinal mood monitoring in bipolar disorder: course of illness as revealed through a short messaging service. J Affect Disord. 2017;223:139–45. 28753472 10.1016/j.jad.2017.07.029

[CR66] Moraga SBF, Doornbos B, Bruggeman R, van der Krieke L, Snippe E, Aarts E. Evidence for severe mood instability in patients with bipolar disorder: Applying multilevel hidden Markov modelling to intensive longitudinal ecological momentary assessment data. 2023. 10.1037/abn0000915 38829323

[CR67] Morriss RK, Faizal MA, Jones AP, Williamson PR, Bolton C, McCarthy JP. Interventions for helping people recognise early signs of recurrence in bipolar disorder. Cochrane Database Syst Rev. 2007;1:Cd004854. 10.1002/14651858.CD004854.pub2 PMC6544804 17253526

[CR68] Nelson B, McGorry PD, Wichers M, Wigman JTW, Hartmann JA. Moving from static to dynamic models of the onset of mental disorder: a review. JAMA Psychiat. 2017;74(5):528–34. 10.1001/jamapsychiatry.2017.0001 28355471

[CR69] O’Rourke N, Sixsmith A, King DB, Yaghoubi-Shahir H, Canham SL. Development and validation of the BD SX: a brief measure of mood and symptom variability for use with adults with bipolar disorder. Int J Bipolar Disord. 2016;4(1):8. 26928123 10.1186/s40345-016-0048-2 PMC4771673

[CR70] O’Rourke N, Sixsmith A. Ecological momentary assessment of mood and movement with bipolar disorder over time: participant recruitment and efficacy of study methods. Int J Methods Psychiatr Res. 2021. 10.1002/mpr.1895 PMC8633933 34652054

[CR71] Ortiz A, Maslej MM, Husain MI, Daskalakis ZJ, Mulsant BH. Apps and gaps in bipolar disorder: a systematic review on electronic monitoring for episode prediction. J Affect Disord. 2021;295:1190–200. 34706433 10.1016/j.jad.2021.08.140

[CR72] Ortiz A, Park C, Gonzalez-Torres C, Alda M, Blumberger D, Husain I, et al. Predictors of adherence to electronic self-monitoring in patients with bipolar disorder: a contactless study using growth mixture models. Eur Psychiatry. 2023;66(Supplement 1):S504–5. 10.1186/s40345-023-00297-5 PMC10192477 37195477

[CR73] Osher Y, Bersudsky Y, O'Rourke N, Belotherkovsky D, Bachner YG. Clinical validation of the BDSx scale with bipolar disorder outpatients. Arch Psychiatr Nurs. 2020 Feb;34(1):49-52. 10.1016/j.apnu.2019.11.002. 10.1016/j.apnu.2019.11.002 32035589

[CR75] Pahwa M, McElroy SL, Priesmeyer R, Siegel G, Siegel P, Nuss S, et al. KIOS: a smartphone app for self-monitoring for patients with bipolar disorder. Bipolar Disord. 2024;26(1):84–92. 37340215 10.1111/bdi.13362 PMC10730767

[CR76] Palmier-Claus J, Lobban F, Mansell W, Jones S, Tyler E, Lodge C, et al. Mood monitoring in bipolar disorder: is it always helpful? Bipolar Disord. 2021;23(4):429–31. 33570820 10.1111/bdi.13057

[CR77] Perez Arribas I, Goodwin GM, Geddes JR, Lyons T, Saunders KEA. A signature-based machine learning model for distinguishing bipolar disorder and borderline personality disorder. Transl Psychiatry. 2018;8(1):274. 30546013 10.1038/s41398-018-0334-0 PMC6293318

[CR78] Petzold J, Mayer-Pelinski R, Pilhatsch M, Luthe S, Barth T, Bauer M, et al. Short group psychoeducation followed by daily electronic self-monitoring in the long-term treatment of bipolar disorders: a multicenter, rater-blind, randomized controlled trial. Int J Bipolar Disord. 2019;7(1):23. 31680193 10.1186/s40345-019-0158-8 PMC6826047

[CR79] PROSPERO. Mood-tracking and self-monitoring in bipolar disorder and depression. PROSPERO. 2023.

[CR80] The Wellcome Trust. Advancing the use of sensor-based digital health technologies (sDHTs) for mental health research and clinical practice [version 1; not peer reviewed]. Wellcome Open Res. 2025;10:74. 10.21955/wellcomeopenres.1115405.1.

[CR81] Rush A, Trivedi MH, Ibrahim HM, Carmody TJ, Arnow B, Klein DN, et al. The 16-item Quick Inventory of Depressive Symptomatology (QIDS), clinician rating (QIDS-C), and self-report (QIDS-SR): a psychometric evaluation in patients with chronic major depression. Biol Psychiatry. 2003;54(5):573–83. 12946886 10.1016/s0006-3223(02)01866-8

[CR82] Schärer LO, Krienke UJ, Graf SM, Meltzer K, Langosch JM. Validation of life-charts documented with the personal life-chart app - a self-monitoring tool for bipolar disorder. BMC Psychiatry. 2015;15:49. 25885225 10.1186/s12888-015-0414-0 PMC4367878

[CR83] Schneider J, Bakštein E, Kolenič M, Vostatek P, Correll CU, Novák D, et al. Motor activity patterns can distinguish between interepisode bipolar disorder patients and healthy controls. CNS Spectr. 2022;27(1):82–92. 32883376 10.1017/S1092852920001777

[CR84] Stanislaus S, Faurholt-Jepsen M, Vinberg M, Coello K, Kjærstad HL, Melbye S, et al. Mood instability in patients with newly diagnosed bipolar disorder, unaffected relatives, and healthy control individuals measured daily using smartphones. J Affect Disord. 2020;271:336–44. 32479333 10.1016/j.jad.2020.03.049

[CR85] Sterne JA, Hernán MA, Reeves BC, Savović J, Berkman ND, Viswanathan M, et al. ROBINS-I: a tool for assessing risk of bias in non-randomised studies of interventions. BMJ. 2016;355:i4919. 27733354 10.1136/bmj.i4919 PMC5062054

[CR86] Torous J, Choudhury T, Barnett I, Keshavan M, Kane J. Smartphone relapse prediction in serious mental illness: a pathway towards personalized preventive care. World Psychiatry. 2020;19(3):308–9. 32931109 10.1002/wps.20805 PMC7491614

[CR87] Trull TJ, Ebner-Priemer U. Ambulatory assessment. Annu Rev Clin Psychol. 2013;9:151–76. 23157450 10.1146/annurev-clinpsy-050212-185510 PMC4249763

[CR88] Tseng YC, Lin EC, Wu CH, Huang HL, Chen PS. Associations among smartphone app-based measurements of mood, sleep and activity in bipolar disorder. Psychiatry Res. 2022;310:114425. 35152069 10.1016/j.psychres.2022.114425

[CR89] van den Berg KC, Hendrickson AT, Hales SA, Voncken M, Keijsers GPJ. Comparing the effectiveness of imagery focussed cognitive therapy to group psychoeducation for patients with bipolar disorder: a randomised trial. J Affect Disord. 2023;320:691–700. 36206888 10.1016/j.jad.2022.09.160

[CR90] Van der Watt AS, Odendaal W, Louw K, Seedat S. Distant mood monitoring for depressive and bipolar disorders: a systematic review. BMC Psychiatry. 2020;20(1):1–14. 32698802 10.1186/s12888-020-02782-y PMC7374077

[CR91] Wenze SJ, Miller IW. Use of ecological momentary assessment in mood disorders research. Clin Psychol Rev. 2010;30(6):794–804. 20619520 10.1016/j.cpr.2010.06.007

[CR92] Whybrow PC, Grof P, Gyulai L, Rasgon N, Glenn T, Bauer M. The electronic assessment of the longitudinal course of bipolar disorder: the ChronoRecord software. Pharmacopsychiatry. 2003;36(Suppl 3):S244-9. 14677086 10.1055/s-2003-45137

[CR93] Wright A. The user experience of ecological momentary assessment and mood monitoring in bipolar disorder: a systematic review and meta-synthesis of qualitative studies. 2024. 10.2196/71525 PMC12533931 41105870

[CR94] Wright LA, MM, Reeves S, Perez Vallejos E, Morriss R. Co-production to improve the utility, safety and ethical use of a passive mood tracking application for people with bipolar disorder. Under Rev JMIR Form Res. 2024. 10.2196/65140 PMC11845880 39918865

[CR95] Wright LA. Performance of active and passive ecological momentary assessment measures in depression: a systematic review In press. 2025a.

[CR96] Wright LA, Majid M, Moore M, Momoh G, Patil R, Shajan G, et al. The user experience of ambulatory assessment and mood monitoring in bipolar disorder: systematic review and meta-synthesis of qualitative studies. J Med Internet Res. 2025b;27:e71525. 41105870 10.2196/71525 PMC12533931

[CR97] Wright LA, Monk-Cunliffe J, Guo B, Morriss R. Adverse events of mood monitoring and ambulatory assessment in depression and bipolar disorder: systematic review and meta-analysis. JMIR Ment Health. 2025c;12:e79500. 41129719 10.2196/79500 PMC12548826

[CR98] de Zambotti M, Rosas L, Colrain IM, Baker FC. The sleep of the ring: comparison of the ŌURA sleep tracker against polysomnography. Behav Sleep Med. 2019;17(2):124–36. 28323455 10.1080/15402002.2017.1300587 PMC6095823

