# Supplementary material for: Performance of active and passive ambulatory assessment measures and mood monitoring in bipolar disorder: a systematic review
Source: Int J Bipolar Disord. 2026 Jan 23;14:4. doi: 10.1186/s40345-025-00407-5 (PMC12852565; doi:10.1186/s40345-025-00407-5)
Supplement: Supplementary file 1 — Additional file 1. [file 40345_2025_407_MOESM1_ESM.docx]

| **Supplementary Table 1: Ambulatory assessment protocols of included non-randomised and randomised studies** | | | | | | | | | | | | | | | | | | |
| --- | --- | --- | --- | --- | --- | --- | --- | --- | --- | --- | --- | --- | --- | --- | --- | --- | --- | --- |
| **Ambulatory assessment protocols for non-randomised studies** | | | | | | | | | | | | | | | | | | |
| **Study** | **Country** | | **Sample** | | **n** | | **Mean age in years (SD)** | | **% female** | | **Intervention** | | **Setting** | | **Ambulatory assessment/Mood tracking  Procedure** | | **ambulatory assessment Duration** | **Established Mood Outcome** |
| Anyz et al 2021 | Czech Republic | | Not reporrted | | 99 | | 37.1 (11) | | 61 | | Aktibipo self-rating questionnaire | | Not reported | | ASERT mood self-reports weekly - 10 items that map depressive, manic and nonspecific symptoms on a likert scale. | | 18 months | MADRS, YMRS |
| Hidalgo-Mazzei et al 2016 | Spain | | Bipolar 1: 67.3, Bipolar 2: 26.5 | | 51 | | 43.92 (11.36) | | 42.9 | | SIMPLe 1.0 - Self-report 5 item test assessing mood, energy, sleep duration, medication adherence and irritability - daily. DSM-5 criteria for manic/depressive episodes - weekly. Daily notification of psychoeducation relapse prevention message (500 different messages possible) targeting specific situations based on ambulatory assessment data. | | Tertiary care - mood disorders service | | Self-report 5 item test assessing mood, energy, sleep duration, medication adherence and irritability - daily. DSM-5 criteria for manic/depressive episodes - weekly. | | 3 months | HDRS, YMRS, FAST, Morisky-Green 8-item test - baseline, 3 months |
| Hidalgo-Mazzei et al 2018 | Spain, Argentina | | Not reported | | 201 | | 36.59 (11) | | 63.2 | | SIMPLe 1.5 - Self-report 5 item test assessing mood, energy, sleep duration, medication adherence and irritability - daily. DSM-5 criteria for manic/depressive episodes - weekly. Daily notification of psychoeducation relapse prevention message (500 different messages possible) targeting specific situations based on ambulatory assessment data. Additional app components/modules: medication reminders, personalised prodromal symptoms, gamification module, mood-chart sharing, psychoeducational messages community. | | Tertiary care - mood disorders service | | Self-report 5 item test assessing mood, energy, sleep duration, medication adherence and irritability - daily. DSM-5 criteria for manic/depressive episodes - weekly. | | 12 months | MDQ - baseline, WHO-5, SF-36 - baseline, 6 months |
| Garcia-Estela et al 2022 | Spain, Chile, Argentina, Mexico, Colombia, Guatemala, Brazil, Other | | Not reported | | 503 | | 34.74 (10.48) | | 67.7 | | SIMPLe 1.5 - Self-report 5 item test assessing mood, energy, sleep duration, medication adherence and irritability - daily. DSM-5 criteria for manic/depressive episodes - weekly. Daily notification of psychoeducation relapse prevention message (500 different messages possible) targeting specific situations. Additional app components/modules: medication reminders, personalised prodromal symptoms, gamification module, mood-chart sharing, psychoeducational messages community. | | Mixed sample – participants recruited online. | | Self-report 5 item test assessing mood, energy, sleep duration, medication adherence and irritability - daily. DSM-5 criteria for manic/depressive episodes - weekly. | | 6 months | MDQ - baseline. WHO-5 - baseline, 6 months. Satisfaction and perceived helpfulness, System Usability Scale - 6 months. |
| Bauer et al 2023 | Germany, USA | | Bipolar 1: 55.0%, Bipolar 2: 31.5%, Bipolar NOS: 5.1, Unipolar: 8.4 | | 609 | | 40.3 (11.8) | | 71.4 | | ChronoRecord - daily mood via 100 point VAS, sleep, life events, menstrual data, psychiatric medication, weekly - weight. | | Secondary care - outpatient | | ChronoRecord - daily mood via 100 point VAS, sleep, life events, menstrual data, psychiatric medication, weekly - weight. | | Average follow up: 227 days | Varied between studies and not reported in summary paper |
| Bos et al 2022 | The Netherlands | | Bipolar 1: 40%, Bipolar 2: 60% | | 20 | | 20-35 years: n = 9, 36-50 years: n = 8, 51-65 years: n = 3 | | 80 | | 5x EMA smartphone assessments daily - 29 items assessing monetary mood, symptoms, sleep and activities. Weekly ASRM, QIDS-SR-16 delivered via RoQua platform. | | Tertiary care - mood disorders service | | 5x EMA smartphone assessments daily - 29 items assessing monetary mood, symptoms, sleep and activities. Weekly ASRM, QIDS-SR-16 delivered via RoQua platform. | | 4 months (range: 16-32 weeks) | ASRM, QIDS-SR-16 - weekly |
| Bowden et al 2021 | USA | | Not reported | | 20 | | Not reported | | Not reported | | KIOS app - self report assessment of 8 different symptoms e.g sadness/pessimism and delivery of guidance in relation to symptom change. | | Tertiary care - mood disorders service | | KIOS app - self report assessment of 8 different symptoms e.g sadness/pessimism and delivery of guidance in relation to symptom change. | | 3 months | SCID - baseline. Nil other validated measures. |
| Dominiak et al 2022 | Poland | | Bipolar 1: 61%, Bipolar 2: 39% | | 84 | | 36.2 (9.5) | | 55 | | BDmon app - Passive ambulatory assessment: phone/SMS logs, participant speech information extracted from daily phone calls, Active ambulatory assessment: self report mood | | Mixed sample – participants recruited via inpatient/outpatient services | | BDmon app - Passive ambulatory assessment: phone/SMS logs, participant speech information extracted from daily phone calls, Active ambulatory assessment: self report mood | | mean: 208 days (SD: 32) | HDRS-17, YMRS - 2 weekly |
| Emden et al 2021 | Germany | | Not reported | | Total: 997, Depression: 409, Bipolar Disorder: 48, Anxiety: 58, Psychosis: 21, Healthy controls: 458 | | 35.99 (13.57) | | 67.3 | | ReMAP system | | Unclear - ReMAP offered to a variety of participants already enrolled in multiple longitudinal studies | | ReMAP system - Active ambulatory assessment - single mood likert scale, single item sleep scale assessing self-report sleep time, voice sample - weekly. Passive ambulatory assessment: step-count, GPS location, accelerometer - continuous. | | 12 months | BDI - 2 weekly |
| Stanislaus et al 2020 | Denmark | | Bipolar 1: 33.5, Bipolar 2: 66.5 | | Bipolar Disorder: 203, Unaffected first-degree relatives: 54, HC: 109 | | Median: 28 (IQR: 24-35) | | 69 | | Monsenso system | | Tertiary care - mood disorders service | | Monsenso system - Active ambulatory assessment: daily smartphone self monitoring items - mood and activity level, HDRS-17 & YMRS every 3 days. Passive ambulatory assessment: Objective smartphone data - phone usage, call/SMS logs, step count | | Median 106 days (IQR: 48-204) | SCAN, HDRS-17, YMRS, FAST, IPAQ - baseline and yearly |
| Lee et al 2022 | South Korea | | Bipolar 1: 28.9, Bipolar 2: 35.9, Depression: 35.2 | | Total: 495, Depression: 95, Bipolar Disorder: 175 | | Subjects included in analysis: 23.3 (3.63), subjects not included in analysis: 22.8 (3.10) | | Subjects included in analysis: 54.4, subjects not included in analysis: 59.1 | | eMoodChart system | | Secondary care | | eMoodChart system - Active ambulatory assessment: self report daily mood and energy, . Passive ambulatory assessment: Fitbit measuring step-count, heart rate, sleep, ambient light (android online) | | mean: 279.7 days (SD: 263.5), median: 505 days (range: 72-1515) | Nil other measures |
| Born et al 2014 | Germany | | Bipolar 1: 72.2, Bipolar 2: 25.9 | | 108 | | 39.3 (13.3) | | 47.2 | | NIMH Life Chart Methodology - prospective | | Tertiary care - mood disorders service | | NIMH Life Chart Methodology - prospective —twice daily mood self-rating | | Unclear - potentially 3 years | YMRS, IDS-C, CGI-BP - monthly |
| Lieberman et al 2011 | USA | | Not reported | | 64 | | Not reported | | Not reported | | MoodChart | | Mixed sample – participants recruited online. | | MoodChart - daily self report mood via email/online, Social Rhythm Metric - activity level over the previous 7 days | | mean: 84 (range: 42-90) | Nil other measures |
| Kupka et al 2005 | USA, Netherlands, Germany | | Bipolar 1: 77.7, Bipolar 2: 19.3 | | 539 | | 42.1 (11.5) | | 56 | | NIMH Life Chart Methodology - prospective | | Tertiary care - mood disorders service | | NIMH Life Chart Methodology - prospective — daily mood self-rating | | 1 year | Nil other measures |
| O’Rourke et al 2021 | Canada, USA, UK, South Africa, Australia | | Not reported | | 50 | | 50% >45 years | | Not reported | | Twice daily Bipolar Disorder Symptom Scale | | Mixed sample – participants recruited online. | | Twice daily Bipolar Disorder Symptom Scale | | 4+ months | Nil other measures |
| Tseng et al 2022 | Taiwan | | Not reported | | 159 | | 34.5 (11.34) | | 55.97 | | Smartphone app collecting daily/weekly active and passive ambulatory assessment. | | Secondary care - outpatients | | Active ambulatory assessment: daily mood, sleep duration. Weekly ASRM, DASS-21. Passive ambulatory assessment: GPS location | | number of days on which participants performed self-assessments - mean: 94.25 days (median 52.5, range 2 to 398) | HDRS, YMRS, DASS-21, PSQI - baseline |
| Ebner-Priemer et al 2020 | Germany | | Bipolar 1: 58.6, Bipolar 2: 41.4 | | 31 | | 44 (11.9) | | 55 | | MovisensXS | | Tertiary care - mood disorders service | | MovisensXS system - Active ambulatory assessment: self-report mood, sleep diary.  Passive ambulatory assessment: call/text logs, GPS data, velocity, step-count. | | 12 months | SCID-I section A, YMRS, BRMRS, MADRS - 2 weekly. |
| Gideon et al 2016 | USA | | All participants had rapid cycling | | 37 | | Not reported | | Not reported | | PRIORI app | | Secondary care - outpatients and inpatients | | PRIORI system - records speech made on telephone calls. Weekly HDRS, YMRS. | | 6-12 months. Mean: 29.2 weeks (SD: 16.4) | HDRS, YMRS - weekly |
| Schneider et al 2022 | Czech Republic | | Bipolar 1: 64, Bipolar 2: 36 | | Bipolar disorder: 35, HC: 26 | | 39.75 (SD: 12.85) | | 60 | | MINDPAX - actigraphy | | Tertiary care - mood disorders service | | MINDPAX - actigraphy measuring sleep data | | 3 months | MINI, MADRS, YMRS - baseline |
| Scharer et al 2015 | Germany | | Not reported | | 54 | | 40.6 | | 46.3 | | PLC app | | Mixed sample – participants recruited via advertising | | PLC app - daily self report mood | | 18 months | IDS-C, YMRS - monthly (mean: 32 days, median: 23 days, range: 2 to 205 days) |
| van den Heuvel et al 2018 | Netherlands | | Bipolar 1: 76.7, Bipolar 2: 23.3 | | 66 | | 45.17 (10.67) | | 66.7 | | PHR-BD system | | Tertiary care - mood disorders service | | PHR-BD system - including 9 modules covering: medical record, medication, treatment and medical passport, general information about BD, medical results/reports, platform to send messages to appointed clinician, mood chart with daily self report mood, personal crisis plan. | | 12 months | QBL-NL, OQ-45.2, ASRM, IDS-SR, MANSA, NEL - baseline, 12 months |
| Arribas et al 2018 | UK | | Bipolar Disorder: 53, Borderline Personality Disorder: 33, HC: 53 | | 139 | | Bipolar Disorder: 38 (+/-21), Borderline Personality Disorder: 34 (+/-15), HC:37 (+/-20) | | Bipolar Disorder: 69.8%, Borderline Personality Disorder: 94%, HC: 66% | | AMoSS study system | | Mixed sample – participants recruited via advertising | | AMoSS study system - Active ambulatory assessment: daily mood rating across categories of anxiety, elation, sadness, anger, irritability and energy using MoodZoom questionnaire, ASRM, QIDS-SR16, EQ-5D, GAD-7 assessed weekly. Passive ambulatory assessment: GPS, actigraphy, ambient light, call/SMS logs, heart rate via smartphone/Fitbit/GENEActive accelerometer/Proteus patch (heart rate data only for one week). | | 3 months, with 61 participants continuing for 12 months | KEAS, IPDE - baseline, ASRM, QIDS-SR16, EQ-5D, GAD-7 - weekly |
| Lewis et al 2023 | UK | | Bipolar 1: 61.6, Bipolar 2: 38.4 | | 649 | | 53 (range: 22-83) | | 68 | | Bipolar Disorder Research Network using True Colours | | Mixed sample – participants recruited via secondary care/advertising | | Bipolar Disorder Research Network using True Colours: weekly ASRM/QIDS-16SR | | 21 months | SCAN - baseline, ASRM/QIDS-SR16 weekly |
| McKnight et al 2017 | UK | | Bipolar 1: 62.6, Bipolar 2: 33.3 | | 367 | | 41 (SD: 13.7, range: 16-76) | | 66.7 | | OXTET-1 using True Colours | | Mixed sample – participants recruited via secondary care/advertising | | OXTET-1 using True Colours - ASRM/QIDS-SR16 delivered via weekly SMS/email | | 27.5 ± 22.5 months (range: 1-81) | Clinical interview to confirm diagnosis - baseline, ASRM/QIDS-SR16 weekly |
| Ortiz et al 2023 | Canada | | Bipolar 1: 60.9, Bipolar 2: 39.1 | | 87 | | 38.9 (SD: 12.4) | | 67.8 | | E-monitoring system | | Secondary care | | E-monitoring system - Active ambulatory assessment: daily rating of mood, anxiety, energy level using e-VAS. Weekly: PHQ-9, ASRM. Passive ambulatory assessment: Oura Health Oy 3d accelerometer/hyroscope measuring activity, sleep, Infrared optical pulse measuring heart rate, heart rate variability | | 229.4 days (± 12.4) | Weekly PHQ-9, ASRM |
| **Ambulatory assessment protocols for randomised studies** | | | | | | | | | | | | | | | | | | |
| **Study** | | **Country** | | **Sample** | | **n** | | **Mean age in years (SD)** | | **% female** | | **Intervention** | | **Comparator** | **Setting** | **Ambulatory assessment protocol** | **ambulatory assessment Duration** | **Established Mood Outcome** |
| Bilderbeck et al 2016 | | UK | | Bipolar 1: 65.3%, Bipolar 2: 34.7% | | 121 | | 44 (1) | | 72.7 | | Facilitated Integrated Mood Management (therapist administered psychoeducation) | | Manualised Integrated Mood Management (self-administered psychoeducation) | Primary care – not in current mood episode | QIDS-SR-16, ASRM - administered weekly via TrueColours | 12 months | QIDS-SR-16, ASRM – weekly |
| Castle et al 2018 | | Australia | | Bipolar 1: 71%, Bipolar 2: 28% | | 84 | | Control group: 42.6 (11.3), Treatment group: 41.6 (11.0) | | 84 | | Structured group programme comprising an initial block of 12 weekly sessions with 3 additional monthly booster sessions to support participants in applying knowledge and skills to their lives. Included weekly telephone calls to remind participants of the next group session and to offer support for homework tasks. | | Usual care plus weekly telephone calls | Mixed sample – participants recruited via clinician referral, advertising. | Weekly telephone calls – weekly for 12 weeks. | 12 months | Structured telephone interview to determine relapse – monthly for 12 months, MADRS, YMRS – baseline, 3, 12 months. |
| Denicoff et al 2002 | | USA | | Bipolar 1: 63%, Bipolar 2: 37% | | 52 | | 41.3 (11.4) | | 52 | | year 1: lithium, year 2: switch to carbamazepine, year 3: lithium & carbamazepine | | year 1: carbamazepine, year 2: switch to lithium, year 3: lithium & carbamazepine | Tertiary care – specialist mood disorder service with the majority of patients experiencing rapid cycling | NIMH Life Chart Methodology - prospective —twice daily mood self-rating | 3 years | HAM-D, IDS, YMRS, CGI-BP – monthly |
| Depp et al 2012 | | USA | | Bipolar 1: 90%, Bipolar 2: 10% | | 56 | | Paper chart: 46.1 (13.5), Phone chart: 44.0 (14.0) | | 57.5 | | Paper and pen life charting | | Smartphone based life charting | Mixed sample – participants recruited via advertising. | NIMH Life Chart Methodology - prospective — daily mood self-rating via paper and pen and via smartphone | 3 months | MADRS, YMRS - baseline, 6 weeks, 12 weeks. RBANS - baseline. |
| Faurholt-Jepsen et al 2015 | | Denmark | | Bipolar 1: 67%, Bipolar 2: 33% | | 67 | | 29.3 (8.43) | | 67 | | MONARCA system plus: 1. study nurse reviewing data and contacting patients if sign of deterioration to offer advice 2. self-monitored data graphically visualised | | Normal smartphone use | Tertiary care – specialist mood disorder service for patients with a new diagnosis of bipolar or treatment resistance | Daily smartphone self monitoring - mood, sleep duration, medication taken, activity, irritability, mixed mood, cognitive problems, alcohol consumption, stress, menstruation, individualised EWS | 6 months | HAMD, YMRS, PSS, FAST - monthly |
| Faurholt-Jepsen et al 2019 | | Denmark | | Bipolar 1: 59%, Bipolar 2: 41% | | 129 | | 43 (12) | | 59 | | Monsenso system plus: 1. study nurse reviewing data and contacting patients if sign of deterioration to offer advice 2. self-monitored data graphically visualised | | Normal smartphone use | Tertiary care – specialist mood disorder service for patients with a new diagnosis of bipolar or treatment resistance | Daily smartphone self monitoring items - mood, sleep duration, medication taken, activity, irritability, mixed mood, cognitive problems, alcohol consumption, stress, menstruation, individualised EWS, anxiety, self-defined personal parameters, free-text note.                     Objective smartphone data - phone usage, social activity, step count, GPS location | 9 months | HDRS-17, YMRS, FAST, PSS, WHOQoL-BREF, BDI, ASRM, MARS Roger’s Empowerment Scale - baseline, months 1, 3, 6, 9 |
| Faurholt-Jepsen et al 2020 | | Denmark | | Bipolar 1: 58%, Bipolar 2: 42% | | 98 | | 42.69 (13.46) | | 52 | | Monsenso system plus: 1. study nurse reviewing data and contacting patients if sign of deterioration to offer advice 2. self-monitored data graphically visualised | | Usual care | Tertiary care – specialist mood disorder service for patients with a new diagnosis of bipolar or treatment resistance | Daily smartphone self monitoring items - mood, sleep duration, medication taken, activity, irritability, mixed mood, cognitive problems, alcohol consumption, stress, menstruation, individualised EWS, anxiety, self-defined personal parameters, free-text note.                     Objective smartphone data - phone usage, social activity, step count, GPS location | 6 months | HDRS-17, YMRS, FAST, PSS, WHOQoL-BREF, BDI, HDRS-6, ASRM, RAS, MARS Roger’s Empowerment Scale, RRS, PSWQ, BAS, VSS-A - baseline, months 3 and 6 |
| Gliddon et al. 2018 | | Austrailia & USA | | Bipolar 1: 55%, Bipolar 2: 38% | | 304 | | 39.47 (11.19) | | 82 | | Intervention 1: Discussion forum plus MoodSwings-Plus: MoodSwngs plus additional CBT-based interactive elements – tools to support mood and medication monitoring, life-chart development, cognitive strategies, motivational interviewing techniques, self reflection, problem solving, identification of personal triggers and a relapse prevention plan.  Intervention 2: Discussion forum plus MoodSwings: Online intervention comprising: mood monitoring, assessing prodromal mood states, preventing relapse, setting SMART goals. Online delivery of MAPS (Mood Assessment Prevent SMART) programme. | | Discussion forum | Mixed sample – participants recruited via advertising. | Online mood-monitoring via MoodSwings & MoodSwings-Plus websites | 12 months | MADRS, YMRS, SF-12, Q-LES-Q, MARS, TIME – baseline, 3, 6, 9, 12 months. |
| Goldberg et al. 2006 | | USA | | Bipolar 1: 70%, Bipolar 2: 30% | | 177 | | Intervention: 38.5 (9.1), Control 37.7 (10.5) | | 56.5 | | Lamotrogine monotherapy | | Placebo | Tertiary care – specialist mood disorder service with past year rapid cycling. | NIMH Life Chart Methodology - daily mood self-rating | 6.5 months | NIMH Life Chart Methodology - nil other |
| Goulding et al 2022 | | USA | | Bipolar 1: 100%, Bipolar 2: 0% | | 205 | | 42 (12) | | 61 | | Livewell | | Usual care | Secondary care – 1 previous mood episode in the past year and current care by psychiatrist/nurse practitioner. | Smartphone based self management intervention - daily and weekly check-ins for weeks 1-16. Daily - adherence, sleep, duration, routine, wellness levels. Weekly - symptom severity scoring for all individual DSM-IV mood symptoms. | 4 months | QIDS, YMRS, WHOQOL-BREF - baseline, months 2, 4, 8, 10, 11 |
| Langosch et al 2008 | | Germany | | Bipolar 1: 50%, Bipolar 2: 50% | | 44 | | Quetiapine: 45.4 (11), Valproate 37.8 (13.8) | | 60 | | Quetiapine monotherapy | | Valproate monotherapy | Tertiary care – specialist mood disorder service with past year rapid cycling. | NIMH Life Chart Methodology - daily mood self-rating | 12 months | CGI-BP-II, HDRS, MADRS, YMRS, SAS - weekly for 1st 6 weeks, then monthly until 12 months |
| Lauder et al 2015 | | Australia | | Bipolar 1: 52%, Bipolar 2: 48% | | 156 | | MoodSwings-Plus: 39.87 (11.26), MoodSwings: 41.35 (9.85) | | 62 | | MoodSwings-Plus: MoodSwings plus additional CBT-based interactive elements – tools to support mood and medication monitoring, life-chart development, cognitive strategies, motivational interviewing techniques, self reflection, problem solving, identification of personal triggers and a relapse prevention plan. | | MoodSwings: Online intervention comprising: mood monitoring, assessing prodromal mood states, preventing relapse, setting SMART goals. Online delivery of MAPS (Mood Assessment Prevent SMART) programme. | Mixed sample – participants recruited via clinician referral, advertising. | Online mood-monitoring via MoodSwings & MoodSwings-Plus websites | 12 months | ASRM, MADRS-S, MOS-SSS, Levenson's Internal, Powerful Others and Chance Locus of Control scale, MARS, Exploratory Global Assessment Measures, GSEVDEP, GSEVMANIA, GPF:Depression, GPF: Mania, GQOL, SCID, Self-report Relapse – Baseline, 3, 6, 12 months. |
| Leverich et al 2006 | | USA | | Bipolar 1: 72.3, Bipolar 2: 26.4 | | 159 | | 41.6 (12.2) | | 47.8 | | Buproprion or sertraline or venlafaxine as an adjunct to mood stabilisers | | Buproprion or sertraline or venlafaxine as an adjunct to mood stabilisers | Tertiary care – specialist mood disorder service | NIMH Life Chart Methodology - daily mood self-rating | 12 months | CGI-BP – unclear frequency |
| Lieberman et al 2010 | | USA | | Bipolar 1: 13%, Bipolar 2: 71%, Bipolar NOS: 16% | | 48 | | Paper chart: 39.5 (12.9), Online chart: 35.8 (12.0) | | 75 | | NIMH Life Chart Methodology - prospective — daily mood self-rating | | Online Life Chart adaptation | Secodary care - currently under the care of psychiatrist/nurse practitioner | NIMH Life Chart Methodology - prospective — daily mood self-rating / online Life Chart adaptation | 3 months | NIMH Life Chart Methodology - nil else |
| Pahwa et al 2023 | | USA | | Bipolar 1: 100% | | 122 | | 43.75 (14.05) | | 68.60 | | KIOS app | | eMoods app | Tertiary care | KIOS app - self report assessment of 8 different symptoms e.g sadness/pessimism and delivery of guidance in relation to symptom change.  eMoods app – self report mood and symptoms diary tracking daily outlook, motivation, habits, sleep, medications etc | 52 weeks | BISS - monthly |
| Petzold et al. 2019 | | Germany | | Bipolar 1: 71%, Bipolar 2: 39% | | 73 | | Intervention: 44.32 (11.63), Control: 42.69 (12.34) | | 45 | | 6 weekly group Psychoeducation sessions plus 54 weeks of ChronoRecord | | 6 weekly supportive non-structured group meetings plus 54 weeks of daily unstructured computer-based self-reports/diary | Secondary care – remission for 2 months but 1 mood episode in the past 3 years. | ChronoRecord - daily mood, sleep, life events, menstrual data, psychiatric medication, weekly – weight. | 12.5 months | SCID, YMRS, HDRS-17 - baseline, 1.5 months, then every 3 months up to 24 months. SF-36, GSE, HLOC, PICS - baseline, 1.5, 6, 12, 15, 24 months |
| Van den Berg et al 2023 | | Netherlands | | Bipolar 1: 50%, Bipolar 2: 50% | | 62 | | Intervention: 46.5 (11.1), Control: 42.73 (13.0) | | 58 | | Imagery Focussed CBT | | Group Psychoeducation | Mixed sample – participants recruited via advertising. | NIMH Life Chart Methodology - daily mood and anxiety self-rating. | 4 months | QIDS-SR, ASRM, BAI, ALS-18, Life-Rift, BHS, VAS Imagery, MICQ-BD – weekly |
| **ALS-18 – Affect Lability Score Short Version, ASERT – Aktibipo Self-rating Questionnaire, ASRM – Altman Self-Rated Mania Scale / Altman Self Rating Mania Scale, BAI – Beck Anxiety Inventory, BAS – Behavioural Activation Scale, BDI – Beck Depression Inventory, BHS – Beck Hopelessness Scale, BRMRS – Bech-Rafaelsen Mania Rating Scale, CGI-BP – Clinical Global Impression Scale Bipolar Version, DASS-21 – Depression, Anxiety and Stress Scale 21 item, DSM-5 – Diagnostic and Statistical Manual of Mental Disorders, 5th edition, EMA – Ecological Momentary Assessment, FAST – Functional Assessment Short Test / Functional Assessment Screening Tool, GAD-7 – General Anxiety Disorder-7, GPF – Global Measure of Psychosocial Functioning: Mania, GPF:Mania – Global Measure of Psychosocial Functioning (GPF): Mania, GQOL – Global measure of Quality of Life, GSE – General Self-Efficacy Scale, GSEVDEP – Global measure of Severity of Depression, GSEVMANIA – Global measure of Severity of Mania, HAM-D – Hamilton Depression Rating Scale, HC – Healthy Controls, HDRS-17 – Hamilton Depression Rating Scale 17 item, HDRS6 – 6-item Hamilton Depression Rating Scale, HLOC – Health Locus of Control Scale, IDS – Inventory of Depressive Symptomatology, IDS-C – Inventory of Depressive Symptomatology Clinician Rated, IPAQ – International Physical Activity Questionnaire, Life-Rift – Level of general functioning and coping: Longitudinal Interval Follow-Up Evaluation, MADRAS-S – Montgomery–Asberg Depression Rating Scale Self-Assessment, MADRS – Montgomery–Asberg Depression Rating Scale, MANSA – Manchester Short Assessment of Quality of Life, MARS – Medication Adherence Rating Scale, MDQ – Mood Disorder Questionnaire, MOS-SSS – Medical Outcomes Study Social Support Survey, NEL – 40-item Dutch Questionnaire on Patient Empowerment, NIMH – National Institute of Mental Health, NOS – Not Otherwise Specified, OQ-45.2 – 45 item Outcome Questionnaire, PHR-BD – Personal Health Record for Bipolar Disorder, PICS – Perceived Involvement in Care Scales, PLC – Personal Life Chart App, PSQI – Pittsburgh Sleep Quality Index, PSWQ – Penn State Worry Questionnaire, PSS – Perceived Stress Scale, QBL-NL – Questionnaire for Bipolar Illness Netherlands, QIDS – Quick Inventory of Depressive Symptomatology, QIDS-SR16 – Quick Inventory of Depressive Symptomatology-Self Report-16, RBANS – Repeatable Battery for the Assessment of Neuropsychological Status, ReMAP – Remote Monitoring in Psychiatry, RRS – Ruminative Response Scale, SCAN – Schedules for Clinical Assessment in Neuropsychiatry, SCID – Structured Clinical Interview for DSM-IV, SF-12 – Short Form Health Survey, SF-36 – Short Form Survey 36-item, SMART – Specific Measurable Achievable Realistic Timebound, SMS – Short Messaging Service, VSS-A – Verona Satisfaction Scale – Affective Disorder, WHO-5 – World Health Organisation Five Well-Being Index, WHOQoL-BREF – World Health Organisation Quality of Life Scale, YMRS – Young Mania Rating Scale** | | | | | | | | | | | | | | | | | | |

| **Supplementary Table 2: Performance data for relevant studies - correlation coefficients** | | | | | | | | | |
| --- | --- | --- | --- | --- | --- | --- | --- | --- | --- |
| Original study | Validation‎ study | Statistic | Comparison | Classification | Mood state | n | Correlation coefficient | p.value1 | Correlation strength |
| Anyz et al 2021 | Anyz et al 2021 - https://mental.jmir.org/2021/8/e26348/ | Weighted group level correlation coefficient | ASERT sum of questions about depression / MADRS sum of questions | A-AA vs EM | Depression | 99.00 | 0.51 | <0.001 | Moderate |
| Anyz et al 2021 | Anyz et al 2021 | Weighted group level correlation coefficient | ASERT sum of questions about depression and nonspecific symptoms / MADRS sum of questions | A-AA vs EM | Depression | 99.00 | 0.53 | <0.001 | Moderate |
| Anyz et al 2021 | Anyz et al 2021 | Weighted group level correlation coefficient | ASERT sum of questions about mania / YMRS sum of questions | A-AA vs EM | Mania | 99.00 | 0.32 | <0.001 | Moderate |
| Anyz et al 2021 | Anyz et al 2021 | Weighted group level correlation coefficient | ASERT sum of questions about mania and nonspecific symptoms / YMRS sum of questions | A-AA vs EM | Mania | 99.00 | 0.25 | <0.001 | Weak |
| Anyz et al 2021 | Anyz et al 2021 | Weighted group level correlation coefficient | ASERT sadness / MADRS sadness | A-AA vs EM | Depression | 99.00 | 0.49 | <0.001 | Moderate |
| Anyz et al 2021 | Anyz et al 2021 | Weighted group level correlation coefficient | ASERT future / MADRS pessimism | A-AA vs EM | Depression | 99.00 | 0.41 | <0.001 | Moderate |
| Anyz et al 2021 | Anyz et al 2021 | Weighted group level correlation coefficient | ASERT energy / YMRS energy | A-AA vs EM | Mania | 99.00 | 0.40 | <0.001 | Moderate |
| Anyz et al 2021 | Anyz et al 2021 | Weighted group level correlation coefficient | ASERT acceleration / YMRS speech and thinking | A-AA vs EM | Mania | 99.00 | 0.46 | <0.001 | Moderate |
| Anyz et al 2021 | Anyz et al 2021 | Weighted group level correlation coefficient | ASERT sleep / YMRS sleep | A-AA vs EM | Mania | 99.00 | 0.31 | <0.001 | Moderate |
| Anyz et al 2021 | Anyz et al 2021 | Weighted group level correlation coefficient | ASERT unrest / MADRS internal tension | A-AA vs EM | Depression | 99.00 | 0.31 | <0.001 | Moderate |
| Anyz et al 2021 | Anyz et al 2021 | Weighted group level correlation coefficient | ASERT concentration / MADRS concentration | A-AA vs EM | Depression | 99.00 | 0.46 | <0.001 | Moderate |
| Hidalgo-Mazzei et al 2016, Hidalgo-Mazzei et al 2018, Garcia-Estela et al 2022 | Hidalgo-Mazzei et al 2016 - https://pubmed.ncbi.nlm.nih.gov/27128358/ | Pearson's correlation | Daily SIMPLe test / YMRS | A-AA vs EM | Mania | 51.00 | 0.56 | 0.001 | Moderate |
| Hidalgo-Mazzei et al 2016, Hidalgo-Mazzei et al 2018, Garcia-Estela et al 2022 | Hidalgo-Mazzei et al 2016 | Pearson's correlation | Daily SIMPLe test / HDRS | A-AA vs EM | Depression | 51.00 | -0.36 | 0.01 | Moderate |
| Bauer et al 2023 | Whybrow et al 2003 - https://pubmed.ncbi.nlm.nih.gov/14677086/ and Bauer et al 2004 - https://pubmed.ncbi.nlm.nih.gov/14996143/ | Not reported | ChronoRecord / HDRS-17 | A-AA vs EM | Depression | 281.00 | -0.68 | <0.001 | Moderate |
| Bauer et al 2023 | Whybrow et al 2003 | Not reported | ChronoRecord / BDI | A-AA vs EM | Depression | 281.00 | -0.67 | <0.001 | Moderate |
| Bauer et al 2023 | Whybrow et al 2003 | Not reported | ChronoRecord / YMRS | A-AA vs EM | Mania | 281.00 | 0.40 | <0.001 | Moderate |
| Bauer et al 2023 | Bauer et al 2008 - https://pubmed.ncbi.nlm.nih.gov/18423616/ | Pearson's correlation | ChronoRecord / YMRS inpatients | A-AA vs EM | Mania | 27.00 | 0.61 | <0.001 | Moderate |
| Bauer et al 2023 | Bauer et al 2008 | Pearson's correlation | ChronoRecord / MRS inpatients | A-AA vs EM | Mania | 27.00 | 0.54 | <0.001 | Moderate |
| Bauer et al 2023 | Bauer et al 2008 | Pearson's correlation | ChronoRecord / YMRS only YMRS >12 | A-AA vs EM | Mania | 35.00 | 0.54 | <0.001 | Moderate |
| Bauer et al 2023 | Bauer et al 2008 | Pearson's correlation | ChronoRecord / YMRS only Chronorecord >60 | A-AA vs EM | Mania | 38.00 | 0.70 | <0.001 | Moderate |
| Bauer et al 2023 | Bauer et al 2008 | Pearson's correlation | ChronoRecord / YMRS inpatients plus outpatients with HAMD <8 | A-AA vs EM | Mania | 97.00 | 0.83 | <0.001 | Strong |
| Emden et al 2021 | Goltermann et al 2021 - https://pubmed.ncbi.nlm.nih.gov/33433392/ | ICC | ReMAP BDI-I / non-smartphone based BDI - full sample | A-AA vs EM | Depression | 173.00 | 0.92 |  | Strong |
| Emden et al 2021 | Goltermann et al 2021 | ICC | ReMAP BDI-I / non-smartphone based BDI-I | A-AA vs EM | Depression | 64.00 | 0.92 |  | Strong |
| Emden et al 2021 | Goltermann et al 2021 | ICC | ReMAP BDI-I / non-smartphone based BDI-II | A-AA vs EM | Depression | 109.00 | 0.92 |  | Strong |
| Emden et al 2021 | Goltermann et al 2021 | ICC | ReMAP BDI-I / non-smartphone based BDI ≤1 week interval | A-AA vs EM | Depression | 126.00 | 0.93 |  | Strong |
| Emden et al 2021 | Goltermann et al 2021 | ICC | ReMAP BDI-I / non-smartphone based BDI >1 week interval | A-AA vs EM | Depression | 47.00 | 0.89 |  | Strong |
| Emden et al 2021 | Goltermann et al 2021 | ICC | ReMAP BDI-I / non-smartphone based BDI in affective disorders | A-AA vs EM | Depression | 57.00 | 0.91 |  | Strong |
| Emden et al 2021 | Goltermann et al 2021 | ICC | ReMAP BDI-I / non-smartphone based BDI in age ≤35 | A-AA vs EM | Depression | 131.00 | 0.90 |  | Strong |
| Emden et al 2021 | Goltermann et al 2021 | ICC | ReMAP BDI-I / non-smartphone based BDI in age >35 | A-AA vs EM | Depression | 42.00 | 0.96 |  | Strong |
| Emden et al 2021 | Goltermann et al 2021 | ICC | ReMAP BDI-I / non-smartphone based BDI in male | A-AA vs EM | Depression | 41.00 | 0.97 |  | Strong |
| Emden et al 2021 | Goltermann et al 2021 | ICC | ReMAP BDI-I / non-smartphone based BDI in female | A-AA vs EM | Depression | 132.00 | 0.90 |  | Strong |
| Emden et al 2021 | Goltermann et al 2021 | Pearson's correlation | ReMAP single mood item / BDI-I non-smartphone | A-AA vs EM | Depression | 35.00 | -0.46 | 0.005 | Moderate |
| Emden et al 2021 | Goltermann et al 2021 | Pearson's correlation | ReMAP single mood item / BDI-II non-smartphone | A-AA vs EM | Depression | 23.00 | -0.32 | 0.138 | Moderate |
| Emden et al 2021 | Goltermann et al 2021 | Pearson's correlation | ReMAP single sleep item / BDI-I non-smartphone sleep item | A-AA vs EM | Depression | 36.00 | -0.30 | 0.077 | Weak |
| Emden et al 2021 | Goltermann et al 2021 | Pearson's correlation | ReMAP single sleep item / BDI-II non-smartphone sleep item | A-AA vs EM | Depression | 21.00 | -0.38 | 0.090 | Moderate |
| Kupka et al 2005, Born et al 2014 | Denicoff et al 2000 - https://pubmed.ncbi.nlm.nih.gov/11097079/ | Pearson's correlation | LCM Depression / IDS-C | A-AA vs EM | Depression | 3306.00 | -0.79 | <0.001 | Strong |
| Kupka et al 2005, Born et al 2014 | Denicoff et al 2000 | Pearson's correlation | LCM Mania / YMRS | A-AA vs EM | Mania | 3306.00 | 0.71 | <0.001 | Strong |
| Kupka et al 2005, Born et al 2014 | Denicoff et al 2000 | Pearson's correlation | LCM average / GAF | A-AA vs EM | Functioning | 3306.00 | -0.74 | <0.001 | Strong |
| Kupka et al 2005, Born et al 2014 | Denicoff et al 2000 | Pearson's correlation | LCM Depression / IDS-C | A-AA vs EM | Depression | 196.00 | -0.84 | <0.001 | Strong |
| Kupka et al 2005, Born et al 2014 | Denicoff et al 2000 | Pearson's correlation | LCM Mania / YMRS | A-AA vs EM | Depression | 196.00 | 0.75 | <0.001 | Strong |
| Kupka et al 2005, Born et al 2014 | Denicoff et al 2000 | Pearson's correlation | LCM average / GAF | A-AA vs EM | Functioning | 196.00 | -0.80 | <0.001 | Strong |
| Kupka et al 2005, Born et al 2014 | Denicoff et al 2000 | Pearson's correlation | LCM / IDS-C | A-AA vs EM | Depression | 270.00 | -0.79 | <0.001 | Strong |
| Kupka et al 2005, Born et al 2014 | Denicoff et al 2000 | Pearson's correlation | LCM / YMRS | A-AA vs EM | Mania | 270.00 | 0.66 | <0.001 | Moderate |
| Kupka et al 2005, Born et al 2014 | Denicoff et al 2000 | Pearson's correlation | LCM / GAF | A-AA vs EM | Functioning | 270.00 | -0.73 | <0.001 | Strong |
| Kupka et al 2005, Born et al 2014 | Born et al 2014 -https://pubmed.ncbi.nlm.nih.gov/24886463/ | Pearson's correlation | LCM / IDS-C | A-AA vs EM | Depression | 108.00 | -0.72 | <0.001 | Strong |
| Kupka et al 2005, Born et al 2014 | Born et al 2014 | Pearson's correlation | LCM / YMRS | A-AA vs EM | Mania | 108.00 | 0.49 | <0.001 | Moderate |
| Kupka et al 2005, Born et al 2014 | Born et al 2014 | Pearson's correlation | LCM mania / CGI-BP Mania | A-AA vs EM | Mania | 108.00 | 0.49 | <0.001 | Moderate |
| Kupka et al 2005, Born et al 2014 | Born et al 2014 | Pearson's correlation | LCM depression / CGI-BP Depression | A-AA vs EM | Depression | 108.00 | 0.72 | <0.001 | Strong |
| Kupka et al 2005, Born et al 2014 | Born et al 2014 | Pearson's correlation | LCM / CGI-BP Overall Mood | A-AA vs EM | Overall Mood | 108.00 | 0.65 | <0.001 | Moderate |
| O'Rouke et al 2021 | O'Rouke et al 2021 - https://pubmed.ncbi.nlm.nih.gov/26928123/ | Pearson's correlation | BDSX somatic/depressive symptoms / PHQ-9 | A-AA vs EM | Depression | 411.00 | 0.71 | <0.01 | Strong |
| O'Rouke et al 2021 | O'Rouke et al 2021 | Pearson's correlation | BDSX somatic/depressive symptoms / PHQ-9 | A-AA vs EM | Depression | 411.00 | 0.49 | <0.01 | Moderate |
| O'Rouke et al 2021 | O'Rouke et al 2021 | Pearson's correlation | BDSX affrontive symptoms of mania / PHQ-9 | A-AA vs EM | Depression | 411.00 | 0.50 | <0.01 | Moderate |
| O'Rouke et al 2021 | O'Rouke et al 2021 | Pearson's correlation | BDSX elation/loss of insight factor / PHQ-9 | A-AA vs EM | Depression | 411.00 | -0.03 | 0.38 | Weak |
| O'Rouke et al 2021 | Osher et al 2020 - https://pubmed.ncbi.nlm.nih.gov/32035589/ | Pearson's correlation | BDSX depressive subscale / HDRS-6 | A-AA vs EM | Depression | 60.00 | 0.75 | <0.01 | Strong |
| O'Rouke et al 2021 | Osher et al 2020 | Pearson's correlation | BDSX hypo/mania subscale / ASRM | A-AA vs EM | Mania | 60.00 | 0.35 | <0.01 | Moderate |
| O'Rouke et al 2021 | Osher et al 2020 | Pearson's correlation | BDSX depressive subscale / HDRS-17 | A-AA vs EM | Depression | 60.00 | 0.72 | <0.01 | Strong |
| O'Rouke et al 2021 | Osher et al 2020 | Pearson's correlation | BDSX hypo/mania subscale / YMRS | A-AA vs EM | Mania | 60.00 | 0.44 | <0.01 | Moderate |
| Tseng et al 2022 | Tseng et al 2022 - https://pubmed.ncbi.nlm.nih.gov/35152069/ | simple correlations | YMRS total score / Daily mood | A-AA vs EM | Mania | 159.00 | 0.03 | 0.538 | Weak |
| Tseng et al 2022 | Tseng et al 2022 | simple correlations | YMRS sub-item 1 (level of elevated mood) / Daily mood | A-AA vs EM | Mania | 159.00 | 0.11 | 0.021 | Weak |
| Tseng et al 2022 | Tseng et al 2022 | simple correlations | HDRS total score / Daily mood | A-AA vs EM | Depression | 159.00 | -0.25 | <0.001 | Weak |
| Tseng et al 2022 | Tseng et al 2022 | simple correlations | HDRS sub-item 1 (level of decreased mood) / Daily mood | A-AA vs EM | Depression | 159.00 | -0.23 | <0.001 | Weak |
| Tseng et al 2022 | Tseng et al 2022 | simple correlations | ASRM total score / Daily mood | A-AA vs EM | Mania | 159.00 | 0.03 | 0.51 | Weak |
| Tseng et al 2022 | Tseng et al 2022 | simple correlations | ASRM sub-item 1 (elevated mood) / Daily mood | A-AA vs EM | Mania | 159.00 | 0.16 | <0.001 | Weak |
| Tseng et al 2022 | Tseng et al 2022 | simple correlations | DASS-21 depression subscale / Daily mood | A-AA vs EM | Depression | 159.00 | -0.36 | <0.001 | Moderate |
| Tseng et al 2022 | Tseng et al 2022 | simple correlations | YMRS total score / Sleep duration | A-AA vs EM | Mania | 159.00 | -0.06 | 0.397 | Weak |
| Tseng et al 2022 | Tseng et al 2022 | simple correlations | YMRS sub-item 4 (reduced sleep duration) / Sleep duration | A-AA vs EM | Mania | 159.00 | 0.01 | 0.867 | Weak |
| Tseng et al 2022 | Tseng et al 2022 | simple correlations | HDRS total score / Sleep duration | A-AA vs EM | Depression | 159.00 | 0.02 | 0.731 | Weak |
| Tseng et al 2022 | Tseng et al 2022 | simple correlations | HDRS sub-item 4 (problems falling asleep) / Sleep duration | A-AA vs EM | Depression | 159.00 | -0.05 | 0.512 | Weak |
| Tseng et al 2022 | Tseng et al 2022 | simple correlations | HDRS sub-item 4 (problems with mid-sleep wake-ups) / Sleep duration | A-AA vs EM | Depression | 159.00 | 0.01 | 0.912 | Weak |
| Tseng et al 2022 | Tseng et al 2022 | simple correlations | HDRS sub-item 6 (problems with early-morning awakening) / Sleep duration | A-AA vs EM | Depression | 159.00 | -0.09 | 0.176 | Weak |
| Tseng et al 2022 | Tseng et al 2022 | simple correlations | ASRM total score / Sleep duration | A-AA vs EM | Mania | 159.00 | -0.03 | 0.624 | Weak |
| Tseng et al 2022 | Tseng et al 2022 | simple correlations | ASRM sub-item 3 (decreased need for sleep) / Sleep duration | A-AA vs EM | Mania | 159.00 | -0.05 | 0.374 | Weak |
| Tseng et al 2022 | Tseng et al 2022 | simple correlations | PSQI total score / Sleep duration | A-AA vs EM | Sleep | 159.00 | 0.06 | 0.677 | Weak |
| Tseng et al 2022 | Tseng et al 2022 | simple correlations | PSQI sleep duration / Sleep duration | A-AA vs EM | Sleep | 159.00 | 0.32 | 0.016* | Moderate |
| Tseng et al 2022 | Tseng et al 2022 | simple correlations | YMRS total score / Total daily distance of movement | P-AA vs EM | Mania | 159.00 | -0.09 | 0.458 | Weak |
| Tseng et al 2022 | Tseng et al 2022 | simple correlations | YMRS sub-item 2 (level of increased motor activity) / Total daily distance of movement | P-AA vs EM | Mania | 159.00 | 0.00 | 0.993 | Weak |
| Tseng et al 2022 | Tseng et al 2022 | simple correlations | HDRS total score / Total daily distance of movement | P-AA vs EM | Depression | 159.00 | -0.13 | 0.255 | Weak |
| Tseng et al 2022 | Tseng et al 2022 | simple correlations | HDRS sub-item 8 (level of psychomotor retardation) / Total daily distance of movement | P-AA vs EM | Depression | 159.00 | -0.21 | 0.068 | Weak |
| Tseng et al 2022 | Tseng et al 2022 | simple correlations | ASRM total score / Total daily distance of movement | P-AA vs EM | Mania | 159.00 | -0.03 | 0.802 | Weak |
| Tseng et al 2022 | Tseng et al 2022 | simple correlations | ASRM sub-item 5 (psychomotor agitation) / Total daily distance of movement | P-AA vs EM | Mania | 159.00 | 0.05 | 0.583 | Weak |
| Tseng et al 2022 | Tseng et al 2022 | simple correlations | Frequency of exercise / Total daily distance of movement | P-AA vs P-AA | Overall Mood | 159.00 | 0.08 | 0.552 | Weak |
| Tseng et al 2022 | Tseng et al 2022 | simple correlations | Frequency of high-intensity exercise / Total daily distance of movement | P-AA vs P-AA | Overall Mood | 159.00 | 0.06 | 0.655 | Weak |
| Tseng et al 2022 | Tseng et al 2022 - https://pubmed.ncbi.nlm.nih.gov/35152069/ | Repeated measures correlation coefficient | Day-to-day repeat measures correlations between Daily Mood Time 1 / Daily Mood Time 2 | A-AA vs A-AA | Overall Mood | 159.00 | 0.32 | <0.001 | Moderate |
| Tseng et al 2022 | Tseng et al 2022 | Repeated measures correlation coefficient | Day-to-day repeat measures correlations between Daily Mood Time 1 / Sleep Duration Time 2 | A-AA vs A-AA | Overall Mood | 159.00 | 0.01 | 0.595 | Weak |
| Tseng et al 2022 | Tseng et al 2022 | Repeated measures correlation coefficient | Day-to-day repeat measures correlations between Daily Mood Time 1 / Total daily distance of movement Time 2 | A-AA vs P-AA | Overall Mood | 159.00 | 0.08 | 0.073 | Weak |
| Tseng et al 2022 | Tseng et al 2022 | Repeated measures correlation coefficient | Day-to-day repeat measures correlations between Sleep Duration Time 1 / Daily Mood Time 2 | A-AA vs A-AA | Sleep | 159.00 | 0.03 | 0.154 | Weak |
| Tseng et al 2022 | Tseng et al 2022 | Repeated measures correlation coefficient | Day-to-day repeat measures correlations between Sleep Duration Time 1 / Sleep Duration Time 2 | A-AA vs A-AA | Sleep | 159.00 | 0.21 | <0.001 | Weak |
| Tseng et al 2022 | Tseng et al 2022 | Repeated measures correlation coefficient | Day-to-day repeat measures correlations between Sleep Duration Time 1 / Total daily distance of movement Time 2 | A-AA vs P-AA | Sleep | 159.00 | -0.03 | 0.652 | Weak |
| Tseng et al 2022 | Tseng et al 2022 | Repeated measures correlation coefficient | Day-to-day repeat measures correlations between Total daily distance of movement Time 1 / Daily Mood Time 2 | A-AA vs A-AA | Physical Activity | 159.00 | 0.06 | 0.168 | Weak |
| Tseng et al 2022 | Tseng et al 2022 | Repeated measures correlation coefficient | Day-to-day repeat measures correlations between Total daily distance of movement Time 1 / Sleep Duration Time 2 | A-AA vs A-AA | Physical Activity | 159.00 | 0.02 | 0.722 | Weak |
| Tseng et al 2022 | Tseng et al 2022 | Repeated measures correlation coefficient | Day-to-day repeat measures correlations between Total daily distance of movement Time 1 / Total daily distance of movement Time 2 | A-AA vs P-AA | Physical Activity | 159.00 | 0.39 | <0.001 | Moderate |
| Tseng et al 2022 | Tseng et al 2022 | Repeated measures correlation coefficient | Week-to-week repeat measures correlations between Daily Mood Time 1 / Daily Mood Time 2 | A-AA vs A-AA | Overall Mood | 159.00 | 0.39 | <0.001 | Moderate |
| Tseng et al 2022 | Tseng et al 2022 | Repeated measures correlation coefficient | Week-to-week repeat measures correlations between Daily Mood Time 1 / Sleep Duration Time 2 | A-AA vs A-AA | Overall Mood | 159.00 | -0.04 | 0.099 | Weak |
| Tseng et al 2022 | Tseng et al 2022 | Repeated measures correlation coefficient | Week-to-week repeat measures correlations between Daily Mood Time 1 / Total daily distance of movement Time 2 | A-AA vs P-AA | Overall Mood | 159.00 | 0.21 | <0.001 | Weak |
| Tseng et al 2022 | Tseng et al 2022 | Repeated measures correlation coefficient | Week-to-week repeat measures correlations between Sleep Duration Time 1 / Daily Mood Time 2 | A-AA vs A-AA | Sleep | 159.00 | 0.07 | 0.002 | Weak |
| Tseng et al 2022 | Tseng et al 2022 | Repeated measures correlation coefficient | Week-to-week repeat measures correlations between Sleep Duration Time 1 / Sleep Duration Time 2 | A-AA vs A-AA | Sleep | 159.00 | 0.21 | <0.001 | Weak |
| Tseng et al 2022 | Tseng et al 2022 | Repeated measures correlation coefficient | Week-to-week repeat measures correlations between Sleep Duration Time 1 / Total daily distance of movement Time 2 | A-AA vs P-AA | Sleep | 159.00 | 0.34 | <0.001 | Moderate |
| Tseng et al 2022 | Tseng et al 2022 | Repeated measures correlation coefficient | Week-to-week repeat measures correlations between Total daily distance of movement Time 1 / Daily Mood Time 2 | A-AA vs A-AA | Physical Activity | 159.00 | 0.30 | <0.001 | Weak |
| Tseng et al 2022 | Tseng et al 2022 | Repeated measures correlation coefficient | Week-to-week repeat measures correlations between Total daily distance of movement Time 1 / Sleep Duration Time 2 | A-AA vs A-AA | Physical Activity | 159.00 | 0.26 | <0.001 | Weak |
| Tseng et al 2022 | Tseng et al 2022 | Repeated measures correlation coefficient | Week-to-week repeat measures correlations between Total daily distance of movement Time 1 / Total daily distance of movement Time 2 | A-AA vs P-AA | Physical Activity | 159.00 | 0.77 | <0.001 | Strong |
| Tseng et al 2022 | Tseng et al 2022 | Repeated measures correlation coefficient | Month-to-month repeat measures correlations between Daily Mood Time 1 / Daily Mood Time 2 | A-AA vs A-AA | Overall Mood | 159.00 | 0.05 | <0.001 | Weak |
| Tseng et al 2022 | Tseng et al 2022 | Repeated measures correlation coefficient | Month-to-month repeat measures correlations between Daily Mood Time 1 / Sleep Duration Time 2 | A-AA vs A-AA | Overall Mood | 159.00 | 0.02 | 0.2 | Weak |
| Tseng et al 2022 | Tseng et al 2022 | Repeated measures correlation coefficient | Month-to-month repeat measures correlations between Daily Mood Time 1 / Total daily distance of movement Time 2 | A-AA vs P-AA | Overall Mood | 159.00 | 0.20 | <0.001 | Weak |
| Tseng et al 2022 | Tseng et al 2022 | Repeated measures correlation coefficient | Month-to-month repeat measures correlations between Sleep Duration Time 1 / Daily Mood Time 2 | A-AA vs A-AA | Sleep | 159.00 | 0.01 | 0.572 | Weak |
| Tseng et al 2022 | Tseng et al 2022 | Repeated measures correlation coefficient | Month-to-month repeat measures correlations between Sleep Duration Time 1 / Sleep Duration Time 2 | A-AA vs A-AA | Sleep | 159.00 | 0.10 | <0.001 | Weak |
| Tseng et al 2022 | Tseng et al 2022 | Repeated measures correlation coefficient | Month-to-month repeat measures correlations between Sleep Duration Time 1 / Total daily distance of movement Time 2 | A-AA vs P-AA | Sleep | 159.00 | 0.68 | <0.001 | Moderate |
| Tseng et al 2022 | Tseng et al 2022 | Repeated measures correlation coefficient | Month-to-month repeat measures correlations between Total daily distance of movement Time 1 / Daily Mood Time 2 | A-AA vs A-AA | Physical Activity | 159.00 | 0.21 | <0.001 | Weak |
| Tseng et al 2022 | Tseng et al 2022 | Repeated measures correlation coefficient | Month-to-month repeat measures correlations between Total daily distance of movement Time 1 / Sleep Duration Time 2 | A-AA vs A-AA | Physical Activity | 159.00 | 0.66 | <0.001 | Moderate |
| Tseng et al 2022 | Tseng et al 2022 | Repeated measures correlation coefficient | Month-to-month repeat measures correlations between Total daily distance of movement Time 1 / Total daily distance of movement Time 2 | P-AA vs P-AA | Physical Activity | 159.00 | 0.81 | <0.001 | Strong |
| Ebner-Priemer et al 2020 | Ebner-Priemer et al 2020 - https://pubmed.ncbi.nlm.nih.gov/33211262/ Additional File 3 | Within-person correlations of latent factors | Depression / Sleep | A-AA vs A-AA | Depression | 29.00 | -0.01 |  | Weak |
| Ebner-Priemer et al 2020 | Ebner-Priemer et al 2020 | Within-person correlations of latent factors | Depression / Activity | A-AA vs P-AA | Depression | 29.00 | -0.14 |  | Weak |
| Ebner-Priemer et al 2020 | Ebner-Priemer et al 2020 | Within-person correlations of latent factors | Depression / Communicativeness | A-AA vs P-AA | Depression | 29.00 | 0.00 |  | Weak |
| Ebner-Priemer et al 2020 | Ebner-Priemer et al 2020 | Within-person correlations of latent factors | Activity / Sleep | A-AA vs P-AA | Depression | 29.00 | -0.26 |  | Weak |
| Ebner-Priemer et al 2020 | Ebner-Priemer et al 2020 | Within-person correlations of latent factors | Communicativeness / Sleep | A-AA vs P-AA | Depression | 29.00 | -0.14 |  | Weak |
| Ebner-Priemer et al 2020 | Ebner-Priemer et al 2020 | Within-person correlations of latent factors | Communicativeness / Activity | P-AA vs P-AA | Depression | 29.00 | 0.17 |  | Weak |
| Ebner-Priemer et al 2020 | Ebner-Priemer et al 2020 | Within-person correlations of latent factors | Mania / Sleep | A-AA vs A-AA | Mania | 29.00 | -0.13 |  | Weak |
| Ebner-Priemer et al 2020 | Ebner-Priemer et al 2020 | Within-person correlations of latent factors | Mania / Activity | A-AA vs P-AA | Mania | 29.00 | 0.15 |  | Weak |
| Ebner-Priemer et al 2020 | Ebner-Priemer et al 2020 | Within-person correlations of latent factors | Mania / Communicativeness | A-AA vs P-AA | Mania | 29.00 | 0.02 |  | Weak |
| Ebner-Priemer et al 2020 | Ebner-Priemer et al 2020 | Within-person correlations of latent factors | Activity / Sleep | A-AA vs P-AA | Mania | 29.00 | -0.26 |  | Weak |
| Ebner-Priemer et al 2020 | Ebner-Priemer et al 2020 | Within-person correlations of latent factors | Communicativeness / Sleep | A-AA vs P-AA | Mania | 29.00 | -0.14 |  | Weak |
| Ebner-Priemer et al 2020 | Ebner-Priemer et al 2020 | Within-person correlations of latent factors | Communicativeness / Activity | P-AA vs P-AA | Mania | 29.00 | 0.17 |  | Weak |
| Ebner-Priemer et al 2020 | Ebner-Priemer et al 2020 - https://pubmed.ncbi.nlm.nih.gov/33211262/ Additional File 2 | Within-person correlations of latent factors | Hours asleep / Sleep wake changes | A-AA vs A-AA | Overall Mood | 29.00 | 0.19 |  | Weak |
| Ebner-Priemer et al 2020 | Ebner-Priemer et al 2020 | Within-person correlations of latent factors | Hours asleep / Wakeup time | A-AA vs A-AA | Overall Mood | 29.00 | 0.39 |  | Moderate |
| Ebner-Priemer et al 2020 | Ebner-Priemer et al 2020 | Within-person correlations of latent factors | Sleep wake changes / Wakeup time | A-AA vs A-AA | Overall Mood | 29.00 | -0.15 |  | Weak |
| Ebner-Priemer et al 2020 | Ebner-Priemer et al 2020 | Within-person correlations of latent factors | Phone calls out / Phone calls missed | P-AA vs P-AA | Overall Mood | 29.00 | 0.29 |  | Weak |
| Ebner-Priemer et al 2020 | Ebner-Priemer et al 2020 | Within-person correlations of latent factors | Phone calls out / Phone calls not reach | P-AA vs P-AA | Overall Mood | 29.00 | 0.44 |  | Moderate |
| Ebner-Priemer et al 2020 | Ebner-Priemer et al 2020 | Within-person correlations of latent factors | Phone calls out / Total call duration | P-AA vs P-AA | Overall Mood | 29.00 | 0.39 |  | Moderate |
| Ebner-Priemer et al 2020 | Ebner-Priemer et al 2020 | Within-person correlations of latent factors | Phone calls out / Number dialogue partners | P-AA vs P-AA | Overall Mood | 29.00 | 0.85 |  | Strong |
| Ebner-Priemer et al 2020 | Ebner-Priemer et al 2020 | Within-person correlations of latent factors | Phone calls out / n contacts SMS | P-AA vs P-AA | Overall Mood | 29.00 | 0.23 |  | Weak |
| Ebner-Priemer et al 2020 | Ebner-Priemer et al 2020 | Within-person correlations of latent factors | Phone calls missed / Phone calls not reached | P-AA vs P-AA | Overall Mood | 29.00 | 0.18 |  | Weak |
| Ebner-Priemer et al 2020 | Ebner-Priemer et al 2020 | Within-person correlations of latent factors | Phone calls missed / Total call duration | P-AA vs P-AA | Overall Mood | 29.00 | 0.13 |  | Weak |
| Ebner-Priemer et al 2020 | Ebner-Priemer et al 2020 | Within-person correlations of latent factors | Phone calls missed / Number dialogue partners | P-AA vs P-AA | Overall Mood | 29.00 | 0.27 |  | Weak |
| Ebner-Priemer et al 2020 | Ebner-Priemer et al 2020 | Within-person correlations of latent factors | Phone calls missed / n contacts SMS | P-AA vs P-AA | Overall Mood | 29.00 | 0.25 |  | Weak |
| Ebner-Priemer et al 2020 | Ebner-Priemer et al 2020 | Within-person correlations of latent factors | Phone calls not reached / Total call duration | P-AA vs P-AA | Overall Mood | 29.00 | 0.56 |  | Moderate |
| Ebner-Priemer et al 2020 | Ebner-Priemer et al 2020 | Within-person correlations of latent factors | Phone calls not reached / Number dialogue partners | P-AA vs P-AA | Overall Mood | 29.00 | 0.10 |  | Weak |
| Ebner-Priemer et al 2020 | Ebner-Priemer et al 2020 | Within-person correlations of latent factors | Phone calls not reached / n contacts SMS | P-AA vs P-AA | Overall Mood | 29.00 | 0.24 |  | Weak |
| Ebner-Priemer et al 2020 | Ebner-Priemer et al 2020 | Within-person correlations of latent factors | Steps / Minutes in vehicle | P-AA vs P-AA | Overall Mood | 29.00 | 0.44 |  | Moderate |
| Ebner-Priemer et al 2020 | Ebner-Priemer et al 2020 | Within-person correlations of latent factors | Steps / Minutes on foot | P-AA vs P-AA | Overall Mood | 29.00 | 0.73 |  | Strong |
| Ebner-Priemer et al 2020 | Ebner-Priemer et al 2020 | Within-person correlations of latent factors | Steps / Minutes still | P-AA vs P-AA | Overall Mood | 29.00 | -0.42 |  | Moderate |
| Ebner-Priemer et al 2020 | Ebner-Priemer et al 2020 | Within-person correlations of latent factors | Steps / Distance travelled fast | P-AA vs P-AA | Overall Mood | 29.00 | 0.29 |  | Weak |
| Ebner-Priemer et al 2020 | Ebner-Priemer et al 2020 | Within-person correlations of latent factors | Steps / Distance travelled slowly | P-AA vs P-AA | Overall Mood | 29.00 | 0.37 |  | Moderate |
| Ebner-Priemer et al 2020 | Ebner-Priemer et al 2020 | Within-person correlations of latent factors | Steps / Acceleration | P-AA vs P-AA | Overall Mood | 29.00 | 0.19 |  | Weak |
| Ebner-Priemer et al 2020 | Ebner-Priemer et al 2020 | Within-person correlations of latent factors | Steps / Duration of accelerometer | P-AA vs P-AA | Overall Mood | 29.00 | 0.18 |  | Weak |
| Ebner-Priemer et al 2020 | Ebner-Priemer et al 2020 | Within-person correlations of latent factors | Steps / Frequency of display on | P-AA vs P-AA | Overall Mood | 29.00 | 0.24 |  | Weak |
| Ebner-Priemer et al 2020 | Ebner-Priemer et al 2020 | Within-person correlations of latent factors | Steps / Duration display on | P-AA vs P-AA | Overall Mood | 29.00 | 0.17 |  | Weak |
| Ebner-Priemer et al 2020 | Ebner-Priemer et al 2020 | Within-person correlations of latent factors | Minutes in vehicle / Minutes on foot | P-AA vs P-AA | Overall Mood | 29.00 | 0.46 |  | Moderate |
| Ebner-Priemer et al 2020 | Ebner-Priemer et al 2020 | Within-person correlations of latent factors | Minutes in vehicle / Minutes still | P-AA vs P-AA | Overall Mood | 29.00 | -0.49 |  | Moderate |
| Ebner-Priemer et al 2020 | Ebner-Priemer et al 2020 | Within-person correlations of latent factors | Minutes in vehicle / Distance travelled fast | P-AA vs P-AA | Overall Mood | 29.00 | 0.60 |  | Moderate |
| Ebner-Priemer et al 2020 | Ebner-Priemer et al 2020 | Within-person correlations of latent factors | Minutes in vehicle / Distance travelled slowly | P-AA vs P-AA | Overall Mood | 29.00 | 0.51 |  | Moderate |
| Ebner-Priemer et al 2020 | Ebner-Priemer et al 2020 | Within-person correlations of latent factors | Minutes in vehicle / Acceleration | P-AA vs P-AA | Overall Mood | 29.00 | 0.13 |  | Weak |
| Ebner-Priemer et al 2020 | Ebner-Priemer et al 2020 | Within-person correlations of latent factors | Minutes in vehicle / Duration of accelerometer | P-AA vs P-AA | Overall Mood | 29.00 | 0.18 |  | Weak |
| Ebner-Priemer et al 2020 | Ebner-Priemer et al 2020 | Within-person correlations of latent factors | Minutes in vehicle / Frequency of display on | P-AA vs P-AA | Overall Mood | 29.00 | 0.18 |  | Weak |
| Ebner-Priemer et al 2020 | Ebner-Priemer et al 2020 | Within-person correlations of latent factors | Minutes in vehicle / Duration display on | P-AA vs P-AA | Overall Mood | 29.00 | 0.12 |  | Weak |
| Ebner-Priemer et al 2020 | Ebner-Priemer et al 2020 | Within-person correlations of latent factors | Minutes on foot / Minutes still | P-AA vs P-AA | Overall Mood | 29.00 | -0.46 |  | Moderate |
| Ebner-Priemer et al 2020 | Ebner-Priemer et al 2020 | Within-person correlations of latent factors | Minutes on foot / Distance travelled fast | P-AA vs P-AA | Overall Mood | 29.00 | 0.27 |  | Weak |
| Ebner-Priemer et al 2020 | Ebner-Priemer et al 2020 | Within-person correlations of latent factors | Minutes on foot / Distance travelled slowly | P-AA vs P-AA | Overall Mood | 29.00 | 0.40 |  | Moderate |
| Ebner-Priemer et al 2020 | Ebner-Priemer et al 2020 | Within-person correlations of latent factors | Minutes on foot / Acceleration | P-AA vs P-AA | Overall Mood | 29.00 | 0.18 |  | Weak |
| Ebner-Priemer et al 2020 | Ebner-Priemer et al 2020 | Within-person correlations of latent factors | Minutes on foot / Duration of accelerometer | P-AA vs P-AA | Overall Mood | 29.00 | 0.16 |  | Weak |
| Ebner-Priemer et al 2020 | Ebner-Priemer et al 2020 | Within-person correlations of latent factors | Minutes on foot / Frequency of display on | P-AA vs P-AA | Overall Mood | 29.00 | 0.23 |  | Weak |
| Ebner-Priemer et al 2020 | Ebner-Priemer et al 2020 | Within-person correlations of latent factors | Minutes on foot / Duration display on | P-AA vs P-AA | Overall Mood | 29.00 | 0.15 |  | Weak |
| Ebner-Priemer et al 2020 | Ebner-Priemer et al 2020 | Within-person correlations of latent factors | Minutes still / Distance travelled fast | P-AA vs P-AA | Overall Mood | 29.00 | -0.33 |  | Moderate |
| Ebner-Priemer et al 2020 | Ebner-Priemer et al 2020 | Within-person correlations of latent factors | Minutes still / Distance travelled slowly | P-AA vs P-AA | Overall Mood | 29.00 | -0.36 |  | Moderate |
| Ebner-Priemer et al 2020 | Ebner-Priemer et al 2020 | Within-person correlations of latent factors | Minutes still / Acceleration | P-AA vs P-AA | Overall Mood | 29.00 | -0.12 |  | Weak |
| Ebner-Priemer et al 2020 | Ebner-Priemer et al 2020 | Within-person correlations of latent factors | Minutes still / Duration of accelerometer | P-AA vs P-AA | Overall Mood | 29.00 | -0.15 |  | Weak |
| Ebner-Priemer et al 2020 | Ebner-Priemer et al 2020 | Within-person correlations of latent factors | Minutes still / Frequency of display on | P-AA vs P-AA | Overall Mood | 29.00 | -0.16 |  | Weak |
| Ebner-Priemer et al 2020 | Ebner-Priemer et al 2020 | Within-person correlations of latent factors | Minutes still / Duration display on | P-AA vs P-AA | Overall Mood | 29.00 | -0.11 |  | Weak |
| Ebner-Priemer et al 2020 | Ebner-Priemer et al 2020 | Within-person correlations of latent factors | Distance travelled fast / Distance travelled slowly | P-AA vs P-AA | Overall Mood | 29.00 | 0.60 |  | Moderate |
| Ebner-Priemer et al 2020 | Ebner-Priemer et al 2020 | Within-person correlations of latent factors | Distance travelled fast / Acceleration | P-AA vs P-AA | Overall Mood | 29.00 | 0.08 |  | Weak |
| Ebner-Priemer et al 2020 | Ebner-Priemer et al 2020 | Within-person correlations of latent factors | Distance travelled fast / Duration of accelerometer | P-AA vs P-AA | Overall Mood | 29.00 | 0.13 |  | Weak |
| Ebner-Priemer et al 2020 | Ebner-Priemer et al 2020 | Within-person correlations of latent factors | Distance travelled fast / Frequency of display on | P-AA vs P-AA | Overall Mood | 29.00 | 0.11 |  | Weak |
| Ebner-Priemer et al 2020 | Ebner-Priemer et al 2020 | Within-person correlations of latent factors | Distance travelled fast / Duration display on | P-AA vs P-AA | Overall Mood | 29.00 | 0.11 |  | Weak |
| Ebner-Priemer et al 2020 | Ebner-Priemer et al 2020 | Within-person correlations of latent factors | Distance travelled slowly / Acceleration | P-AA vs P-AA | Overall Mood | 29.00 | 0.12 |  | Weak |
| Ebner-Priemer et al 2020 | Ebner-Priemer et al 2020 | Within-person correlations of latent factors | Distance travelled slowly / Duration of accelerometer | P-AA vs P-AA | Overall Mood | 29.00 | 0.15 |  | Weak |
| Ebner-Priemer et al 2020 | Ebner-Priemer et al 2020 | Within-person correlations of latent factors | Distance travelled slowly / Frequency of display on | P-AA vs P-AA | Overall Mood | 29.00 | 0.16 |  | Weak |
| Ebner-Priemer et al 2020 | Ebner-Priemer et al 2020 | Within-person correlations of latent factors | Distance travelled slowly / Duration display on | P-AA vs P-AA | Overall Mood | 29.00 | 0.12 |  | Weak |
| Ebner-Priemer et al 2020 | Ebner-Priemer et al 2020 | Within-person correlations of latent factors | Acceleration / Duration of accelerometer | P-AA vs P-AA | Overall Mood | 29.00 | 0.97 |  | Strong |
| Ebner-Priemer et al 2020 | Ebner-Priemer et al 2020 | Within-person correlations of latent factors | Acceleration / Frequency of display on | P-AA vs P-AA | Overall Mood | 29.00 | 0.01 |  | Weak |
| Ebner-Priemer et al 2020 | Ebner-Priemer et al 2020 | Within-person correlations of latent factors | Acceleration / Duration display on | P-AA vs P-AA | Overall Mood | 29.00 | -0.04 |  | Weak |
| Ebner-Priemer et al 2020 | Ebner-Priemer et al 2020 | Within-person correlations of latent factors | Duration of accelerometer / Frequency of display on | P-AA vs P-AA | Overall Mood | 29.00 | 0.01 |  | Weak |
| Ebner-Priemer et al 2020 | Ebner-Priemer et al 2020 | Within-person correlations of latent factors | Duration of accelerometer / Duration display on | P-AA vs P-AA | Overall Mood | 29.00 | -0.04 |  | Weak |
| Ebner-Priemer et al 2020 | Ebner-Priemer et al 2020 | Within-person correlations of latent factors | Frequency of display on / Duration display on | P-AA vs P-AA | Overall Mood | 29.00 | 0.46 |  | Moderate |
| Scharer et al 2015 | Scharer et al 2015 - https://pubmed.ncbi.nlm.nih.gov/25885225/ | Spearman correlations | IDS-C / PLC daily function - PLC German sample | A-AA vs EM | Depression | 44.00 | -0.73 |  | Strong |
| Scharer et al 2015 | Scharer et al 2015 | Spearman correlations | IDS-C / PLC daily mood - PLC German sample | A-AA vs EM | Depression | 44.00 | 0.62 |  | Moderate |
| Scharer et al 2015 | Scharer et al 2015 | Spearman correlations | YMRS / PLC daily function - PLC German sample | A-AA vs EM | Mania | 44.00 | 0.53 |  | Moderate |
| Scharer et al 2015 | Scharer et al 2015 | Spearman correlations | YMRS / PLC daily mood - PLC German sample | A-AA vs EM | Mania | 44.00 | 0.17 |  | Weak |
| Scharer et al 2015 | Scharer et al 2015 | Spearman correlations | IDS-C / PLC daily function - PLC English sample | A-AA vs EM | Depression | 10.00 | -0.72 |  | Strong |
| Scharer et al 2015 | Scharer et al 2015 | Spearman correlations | IDS-C / PLC daily mood - PLC English sample | A-AA vs EM | Depression | 10.00 | 0.60 |  | Moderate |
| Scharer et al 2015 | Scharer et al 2015 | Spearman correlations | YMRS / PLC daily function - PLC English sample | A-AA vs EM | Mania | 10.00 | 0.61 |  | Moderate |
| Scharer et al 2015 | Scharer et al 2015 | Spearman correlations | YMRS / PLC daily mood - PLC English sample | A-AA vs EM | Mania | 10.00 | 0.39 |  | Moderate |
| Arribas et al 2018 | Carr et al 2018 - https://pubmed.ncbi.nlm.nih.gov/29374207/ | Pearson's correlations | Bipolar: Negative principle component of MoodZoom / Acceleration (Standard deviation of the difference between daily and total sinusoids measures of the diurnal rhythm phase) | A-AA vs P-AA | Depression | 54.00 | -0.37 |  | Moderate |
| Arribas et al 2018 | Carr et al 2018 | Pearson's correlations | Bipolar: Negative principle component of MoodZoom / Acceleration (Standard deviation of the difference between daily and total sinusoids measures of the Midline Estimating Statistic of Rhythms) | A-AA vs P-AA | Depression | 54.00 | 0.30 |  | Weak |
| Arribas et al 2018 | Carr et al 2018 | Pearson's correlations | Bipolar: Negative principle component of MoodZoom / Acceleration (Standard deviation of the difference between daily and total sinusoids measures of the diurnal rhythm amplitudes) | A-AA vs P-AA | Depression | 54.00 | 0.23 |  | Weak |
| Arribas et al 2018 | Carr et al 2018 | Pearson's correlations | Bipolar: Negative principle component of MoodZoom / Acceleration (Standard deviation of successive differences between daily and total sinusoids measures of the diurnal rhythm phase) | A-AA vs P-AA | Depression | 54.00 | -0.37 |  | Moderate |
| Arribas et al 2018 | Carr et al 2018 | Pearson's correlations | Bipolar: Negative principle component of MoodZoom / Acceleration (Standard deviation of successive differences between daily and total sinusoids measures of the Midline Estimating Statistic of Rhythms) | A-AA vs P-AA | Depression | 54.00 | 0.33 |  | Moderate |
| Arribas et al 2018 | Carr et al 2018 | Pearson's correlations | Bipolar: Negative principle component of MoodZoom / Acceleration (Standard deviation of successive differences between daily and total sinusoids measures of the diurnal rhythm amplitudes) | A-AA vs P-AA | Depression | 54.00 | -0.04 |  | Weak |
| Arribas et al 2018 | Carr et al 2018 | Pearson's correlations | Bipolar: Positive principle component of MoodZoom / Acceleration (Standard deviation of the difference between daily and total sinusoids measures of the diurnal rhythm phase) | A-AA vs P-AA | Mania | 54.00 | -0.18 |  | Weak |
| Arribas et al 2018 | Carr et al 2018 | Pearson's correlations | Bipolar: Positive principle component of MoodZoom / Acceleration (Standard deviation of the difference between daily and total sinusoids measures of the Midline Estimating Statistic of Rhythms) | A-AA vs P-AA | Mania | 54.00 | 0.15 |  | Weak |
| Arribas et al 2018 | Carr et al 2018 | Pearson's correlations | Bipolar: Positive principle component of MoodZoom / Acceleration (Standard deviation of the difference between daily and total sinusoids measures of the diurnal rhythm amplitudes) | A-AA vs P-AA | Mania | 54.00 | -0.29 |  | Weak |
| Arribas et al 2018 | Carr et al 2018 | Pearson's correlations | Bipolar: Positive principle component of MoodZoom / Acceleration (Standard deviation of successive differences between daily and total sinusoids measures of the diurnal rhythm phase) | A-AA vs P-AA | Mania | 54.00 | -0.17 |  | Weak |
| Arribas et al 2018 | Carr et al 2018 | Pearson's correlations | Bipolar: Positive principle component of MoodZoom / Acceleration (Standard deviation of successive differences between daily and total sinusoids measures of the Midline Estimating Statistic of Rhythms) | A-AA vs P-AA | Mania | 54.00 | 0.25 |  | Weak |
| Arribas et al 2018 | Carr et al 2018 | Pearson's correlations | Bipolar: Positive principle component of MoodZoom / Acceleration (Standard deviation of successive differences between daily and total sinusoids measures of the diurnal rhythm amplitudes) | A-AA vs P-AA | Mania | 54.00 | -0.12 |  | Weak |
| Arribas et al 2018 | Carr et al 2018 | Pearson's correlations | Bipolar: Irritability principle component of MoodZoom / Acceleration (Standard deviation of the difference between daily and total sinusoids measures of the diurnal rhythm phase) | A-AA vs P-AA | Mania | 54.00 | -0.12 |  | Weak |
| Arribas et al 2018 | Carr et al 2018 | Pearson's correlations | Bipolar: Irritability principle component of MoodZoom / Acceleration (Standard deviation of the difference between daily and total sinusoids measures of the Midline Estimating Statistic of Rhythms) | A-AA vs P-AA | Mania | 54.00 | 0.12 |  | Weak |
| Arribas et al 2018 | Carr et al 2018 | Pearson's correlations | Bipolar: Irritability principle component of MoodZoom / Acceleration (Standard deviation of the difference between daily and total sinusoids measures of the diurnal rhythm amplitudes) | A-AA vs P-AA | Mania | 54.00 | 0.02 |  | Weak |
| Arribas et al 2018 | Carr et al 2018 | Pearson's correlations | Bipolar: Irritability principle component of MoodZoom / Acceleration (Standard deviation of successive differences between daily and total sinusoids measures of the diurnal rhythm phase) | A-AA vs P-AA | Mania | 54.00 | -0.07 |  | Weak |
| Arribas et al 2018 | Carr et al 2018 | Pearson's correlations | Bipolar: Irritability principle component of MoodZoom / Acceleration (Standard deviation of successive differences between daily and total sinusoids measures of the Midline Estimating Statistic of Rhythms) | A-AA vs P-AA | Mania | 54.00 | 0.23 |  | Weak |
| Arribas et al 2018 | Carr et al 2018 | Pearson's correlations | Bipolar: Irritability principle component of MoodZoom / Acceleration (Standard deviation of successive differences between daily and total sinusoids measures of the diurnal rhythm amplitudes) | A-AA vs P-AA | Mania | 54.00 | -0.24 |  | Weak |
| Arribas et al 2018 | Carr et al 2018 | Pearson's correlations | Bipolar: Negative principle component of MoodZoom / Sleep (Standard deviation of the difference between daily and total sinusoids measures of the diurnal rhythm phase) | A-AA vs P-AA | Depression | 54.00 | 0.38 |  | Moderate |
| Arribas et al 2018 | Carr et al 2018 | Pearson's correlations | Bipolar: Negative principle component of MoodZoom / Sleep (Standard deviation of the difference between daily and total sinusoids measures of the diurnal rhythm amplitudes) | A-AA vs P-AA | Depression | 54.00 | 0.41 |  | Moderate |
| Arribas et al 2018 | Carr et al 2018 | Pearson's correlations | Bipolar: Negative principle component of MoodZoom / Sleep (Standard deviation of successive differences between daily and total sinusoids measures of the diurnal rhythm phase) | A-AA vs P-AA | Depression | 54.00 | 0.42 |  | Moderate |
| Arribas et al 2018 | Carr et al 2018 | Pearson's correlations | Bipolar: Negative principle component of MoodZoom / Sleep (Standard deviation of successive differences between daily and total sinusoids measures of the diurnal rhythm amplitudes) | A-AA vs P-AA | Depression | 54.00 | 0.25 |  | Weak |
| Arribas et al 2018 | Carr et al 2018 | Pearson's correlations | Bipolar: Positive principle component of MoodZoom / Sleep (Standard deviation of the difference between daily and total sinusoids measures of the diurnal rhythm phase) | A-AA vs P-AA | Mania | 54.00 | 0.47 |  | Moderate |
| Arribas et al 2018 | Carr et al 2018 | Pearson's correlations | Bipolar: Positive principle component of MoodZoom / Sleep (Standard deviation of the difference between daily and total sinusoids measures of the diurnal rhythm amplitudes) | A-AA vs P-AA | Mania | 54.00 | 0.60 |  | Moderate |
| Arribas et al 2018 | Carr et al 2018 | Pearson's correlations | Bipolar: Positive principle component of MoodZoom / Sleep (Standard deviation of successive differences between daily and total sinusoids measures of the diurnal rhythm phase) | A-AA vs P-AA | Mania | 54.00 | 0.42 |  | Moderate |
| Arribas et al 2018 | Carr et al 2018 | Pearson's correlations | Bipolar: Positive principle component of MoodZoom / Sleep (Standard deviation of successive differences between daily and total sinusoids measures of the diurnal rhythm amplitudes) | A-AA vs P-AA | Mania | 54.00 | 0.31 |  | Moderate |
| Arribas et al 2018 | Carr et al 2018 | Pearson's correlations | Bipolar: Irritability principle component of MoodZoom / Sleep (Standard deviation of the difference between daily and total sinusoids measures of the diurnal rhythm phase) | A-AA vs P-AA | Mania | 54.00 | 0.47 |  | Moderate |
| Arribas et al 2018 | Carr et al 2018 | Pearson's correlations | Bipolar: Irritability principle component of MoodZoom / Sleep (Standard deviation of the difference between daily and total sinusoids measures of the diurnal rhythm amplitudes) | A-AA vs P-AA | Mania | 54.00 | 0.62 |  | Moderate |
| Arribas et al 2018 | Carr et al 2018 | Pearson's correlations | Bipolar: Irritability principle component of MoodZoom / Sleep (Standard deviation of successive differences between daily and total sinusoids measures of the diurnal rhythm phase) | A-AA vs P-AA | Mania | 54.00 | 0.53 |  | Moderate |
| Arribas et al 2018 | Carr et al 2018 | Pearson's correlations | Bipolar: Irritability principle component of MoodZoom / Sleep (Standard deviation of successive differences between daily and total sinusoids measures of the diurnal rhythm amplitudes) | A-AA vs P-AA | Mania | 54.00 | 0.30 |  | Weak |
| Arribas et al 2018 | Carr et al 2018 | Pearson's correlations | Bipolar: Negative principle component of MoodZoom / Heart Rate (Standard deviation of the difference between daily and total sinusoids measures of the diurnal rhythm phase) | A-AA vs P-AA | Depression | 54.00 | -0.27 |  | Weak |
| Arribas et al 2018 | Carr et al 2018 | Pearson's correlations | Bipolar: Negative principle component of MoodZoom / Heart Rate (Standard deviation of the difference between daily and total sinusoids measures of the Midline Estimating Statistic of Rhythms) | A-AA vs P-AA | Depression | 54.00 | -0.15 |  | Weak |
| Arribas et al 2018 | Carr et al 2018 | Pearson's correlations | Bipolar: Negative principle component of MoodZoom / Heart Rate (Standard deviation of the difference between daily and total sinusoids measures of the diurnal rhythm amplitudes) | A-AA vs P-AA | Depression | 54.00 | -0.25 |  | Weak |
| Arribas et al 2018 | Carr et al 2018 | Pearson's correlations | Bipolar: Negative principle component of MoodZoom / Heart Rate (Standard deviation of successive differences between daily and total sinusoids measures of the diurnal rhythm phase) | A-AA vs P-AA | Depression | 54.00 | -0.27 |  | Weak |
| Arribas et al 2018 | Carr et al 2018 | Pearson's correlations | Bipolar: Negative principle component of MoodZoom / Heart Rate (Standard deviation of successive differences between daily and total sinusoids measures of the Midline Estimating Statistic of Rhythms) | A-AA vs P-AA | Depression | 54.00 | -0.20 |  | Weak |
| Arribas et al 2018 | Carr et al 2018 | Pearson's correlations | Bipolar: Negative principle component of MoodZoom / Heart Rate (Standard deviation of successive differences between daily and total sinusoids measures of the diurnal rhythm amplitudes) | A-AA vs P-AA | Depression | 54.00 | -0.37 |  | Moderate |
| Arribas et al 2018 | Carr et al 2018 | Pearson's correlations | Bipolar: Positive principle component of MoodZoom / Heart Rate (Standard deviation of the difference between daily and total sinusoids measures of the diurnal rhythm phase) | A-AA vs P-AA | Mania | 54.00 | 0.11 |  | Weak |
| Arribas et al 2018 | Carr et al 2018 | Pearson's correlations | Bipolar: Positive principle component of MoodZoom / Heart Rate (Standard deviation of the difference between daily and total sinusoids measures of the Midline Estimating Statistic of Rhythms) | A-AA vs P-AA | Mania | 54.00 | 0.14 |  | Weak |
| Arribas et al 2018 | Carr et al 2018 | Pearson's correlations | Bipolar: Positive principle component of MoodZoom / Heart Rate (Standard deviation of the difference between daily and total sinusoids measures of the diurnal rhythm amplitudes) | A-AA vs P-AA | Mania | 54.00 | 0.47 |  | Moderate |
| Arribas et al 2018 | Carr et al 2018 | Pearson's correlations | Bipolar: Positive principle component of MoodZoom / Heart Rate (Standard deviation of successive differences between daily and total sinusoids measures of the diurnal rhythm phase) | A-AA vs P-AA | Mania | 54.00 | 0.05 |  | Weak |
| Arribas et al 2018 | Carr et al 2018 | Pearson's correlations | Bipolar: Positive principle component of MoodZoom / Heart Rate (Standard deviation of successive differences between daily and total sinusoids measures of the Midline Estimating Statistic of Rhythms) | A-AA vs P-AA | Mania | 54.00 | 0.23 |  | Weak |
| Arribas et al 2018 | Carr et al 2018 | Pearson's correlations | Bipolar: Positive principle component of MoodZoom / Heart Rate (Standard deviation of successive differences between daily and total sinusoids measures of the diurnal rhythm amplitudes) | A-AA vs P-AA | Mania | 54.00 | 0.14 |  | Weak |
| Arribas et al 2018 | Carr et al 2018 | Pearson's correlations | Bipolar: Irritability principle component of MoodZoom / Heart Rate (Standard deviation of the difference between daily and total sinusoids measures of the diurnal rhythm phase) | A-AA vs P-AA | Mania | 54.00 | -0.18 |  | Weak |
| Arribas et al 2018 | Carr et al 2018 | Pearson's correlations | Bipolar: Irritability principle component of MoodZoom / Heart Rate (Standard deviation of the difference between daily and total sinusoids measures of the Midline Estimating Statistic of Rhythms) | A-AA vs P-AA | Mania | 54.00 | -0.43 |  | Moderate |
| Arribas et al 2018 | Carr et al 2018 | Pearson's correlations | Bipolar: Irritability principle component of MoodZoom / Heart Rate (Standard deviation of the difference between daily and total sinusoids measures of the diurnal rhythm amplitudes) | A-AA vs P-AA | Mania | 54.00 | -0.17 |  | Weak |
| Arribas et al 2018 | Carr et al 2018 | Pearson's correlations | Bipolar: Irritability principle component of MoodZoom / Heart Rate (Standard deviation of successive differences between daily and total sinusoids measures of the diurnal rhythm phase) | A-AA vs P-AA | Mania | 54.00 | -0.09 |  | Weak |
| Arribas et al 2018 | Carr et al 2018 | Pearson's correlations | Bipolar: Irritability principle component of MoodZoom / Heart Rate (Standard deviation of successive differences between daily and total sinusoids measures of the Midline Estimating Statistic of Rhythms) | A-AA vs P-AA | Mania | 54.00 | -0.47 |  | Moderate |
| Arribas et al 2018 | Carr et al 2018 | Pearson's correlations | Bipolar: Irritability principle component of MoodZoom / Heart Rate (Standard deviation of successive differences between daily and total sinusoids measures of the diurnal rhythm amplitudes) | A-AA vs P-AA | Mania | 54.00 | -0.41 |  | Moderate |
| Lewis et al 2023, McKnight et al 2017 | Tsanas et al 2016 - https://pubmed.ncbi.nlm.nih.gov/27449555/ | Spearman correlations | ASRM: Happy / MoodZoom: Anxious | A-AA vs EM | Mania | 130.00 | 0.08 |  | Weak |
| Lewis et al 2023, McKnight et al 2017 | Tsanas et al 2016 | Spearman correlations | ASRM: Confident / MoodZoom: Anxious | A-AA vs EM | Mania | 130.00 | 0.09 |  | Weak |
| Lewis et al 2023, McKnight et al 2017 | Tsanas et al 2016 | Spearman correlations | ASRM: Sleep / MoodZoom: Anxious | A-AA vs EM | Mania | 130.00 | 0.18 |  | Weak |
| Lewis et al 2023, McKnight et al 2017 | Tsanas et al 2016 | Spearman correlations | ASRM: Talkative / MoodZoom: Anxious | A-AA vs EM | Mania | 130.00 | 0.16 |  | Weak |
| Lewis et al 2023, McKnight et al 2017 | Tsanas et al 2016 | Spearman correlations | ASRM: Active / MoodZoom: Anxious | A-AA vs EM | Mania | 130.00 | 0.14 |  | Weak |
| Lewis et al 2023, McKnight et al 2017 | Tsanas et al 2016 | Spearman correlations | ASRM: Happy / MoodZoom: Elated | A-AA vs EM | Mania | 130.00 | 0.26 |  | Weak |
| Lewis et al 2023, McKnight et al 2017 | Tsanas et al 2016 | Spearman correlations | ASRM: Confident / MoodZoom: Elated | A-AA vs EM | Mania | 130.00 | 0.26 |  | Weak |
| Lewis et al 2023, McKnight et al 2017 | Tsanas et al 2016 | Spearman correlations | ASRM: Sleep / MoodZoom: Elated | A-AA vs EM | Mania | 130.00 | 0.16 |  | Weak |
| Lewis et al 2023, McKnight et al 2017 | Tsanas et al 2016 | Spearman correlations | ASRM: Talkative / MoodZoom: Elated | A-AA vs EM | Mania | 130.00 | 0.21 |  | Weak |
| Lewis et al 2023, McKnight et al 2017 | Tsanas et al 2016 | Spearman correlations | ASRM: Active / MoodZoom: Elated | A-AA vs EM | Mania | 130.00 | 0.21 |  | Weak |
| Lewis et al 2023, McKnight et al 2017 | Tsanas et al 2016 | Spearman correlations | ASRM: Happy / MoodZoom: Sad | A-AA vs EM | Mania | 130.00 | 0.07 |  | Weak |
| Lewis et al 2023, McKnight et al 2017 | Tsanas et al 2016 | Spearman correlations | ASRM: Confident / MoodZoom: Sad | A-AA vs EM | Mania | 130.00 | 0.05 |  | Weak |
| Lewis et al 2023, McKnight et al 2017 | Tsanas et al 2016 | Spearman correlations | ASRM: Sleep / MoodZoom: Sad | A-AA vs EM | Mania | 130.00 | 0.15 |  | Weak |
| Lewis et al 2023, McKnight et al 2017 | Tsanas et al 2016 | Spearman correlations | ASRM: Talkative / MoodZoom: Sad | A-AA vs EM | Mania | 130.00 | 0.14 |  | Weak |
| Lewis et al 2023, McKnight et al 2017 | Tsanas et al 2016 | Spearman correlations | ASRM: Active / MoodZoom: Angry | A-AA vs EM | Mania | 130.00 | 0.12 |  | Weak |
| Lewis et al 2023, McKnight et al 2017 | Tsanas et al 2016 | Spearman correlations | ASRM: Happy / MoodZoom: Angry | A-AA vs EM | Mania | 130.00 | 0.07 |  | Weak |
| Lewis et al 2023, McKnight et al 2017 | Tsanas et al 2016 | Spearman correlations | ASRM: Confident / MoodZoom: Angry | A-AA vs EM | Mania | 130.00 | 0.08 |  | Weak |
| Lewis et al 2023, McKnight et al 2017 | Tsanas et al 2016 | Spearman correlations | ASRM: Sleep / MoodZoom: Angry | A-AA vs EM | Mania | 130.00 | 0.18 |  | Weak |
| Lewis et al 2023, McKnight et al 2017 | Tsanas et al 2016 | Spearman correlations | ASRM: Talkative / MoodZoom: Angry | A-AA vs EM | Mania | 130.00 | 0.15 |  | Weak |
| Lewis et al 2023, McKnight et al 2017 | Tsanas et al 2016 | Spearman correlations | ASRM: Active / MoodZoom: Angry | A-AA vs EM | Mania | 130.00 | 0.13 |  | Weak |
| Lewis et al 2023, McKnight et al 2017 | Tsanas et al 2016 | Spearman correlations | ASRM: Happy / MoodZoom: Irritable | A-AA vs EM | Mania | 130.00 | 0.11 |  | Weak |
| Lewis et al 2023, McKnight et al 2017 | Tsanas et al 2016 | Spearman correlations | ASRM: Confident / MoodZoom: Irritable | A-AA vs EM | Mania | 130.00 | 0.12 |  | Weak |
| Lewis et al 2023, McKnight et al 2017 | Tsanas et al 2016 | Spearman correlations | ASRM: Sleep / MoodZoom: Irritable | A-AA vs EM | Mania | 130.00 | 0.24 |  | Weak |
| Lewis et al 2023, McKnight et al 2017 | Tsanas et al 2016 | Spearman correlations | ASRM: Talkative / MoodZoom: Irritable | A-AA vs EM | Mania | 130.00 | 0.20 |  | Weak |
| Lewis et al 2023, McKnight et al 2017 | Tsanas et al 2016 | Spearman correlations | ASRM: Active / MoodZoom: Irritable | A-AA vs EM | Mania | 130.00 | 0.15 |  | Weak |
| Lewis et al 2023, McKnight et al 2017 | Tsanas et al 2016 | Spearman correlations | ASRM: Happy / MoodZoom: Energetic | A-AA vs EM | Mania | 130.00 | 0.19 |  | Weak |
| Lewis et al 2023, McKnight et al 2017 | Tsanas et al 2016 | Spearman correlations | ASRM: Confident / MoodZoom: Energetic | A-AA vs EM | Mania | 130.00 | 0.19 |  | Weak |
| Lewis et al 2023, McKnight et al 2017 | Tsanas et al 2016 | Spearman correlations | ASRM: Sleep / MoodZoom: Energetic | A-AA vs EM | Mania | 130.00 | 0.08 |  | Weak |
| Lewis et al 2023, McKnight et al 2017 | Tsanas et al 2016 | Spearman correlations | ASRM: Talkative / MoodZoom: Energetic | A-AA vs EM | Mania | 130.00 | 0.11 |  | Weak |
| Lewis et al 2023, McKnight et al 2017 | Tsanas et al 2016 | Spearman correlations | ASRM: Active / MoodZoom: Energetic | A-AA vs EM | Mania | 130.00 | 0.19 |  | Weak |
| Lewis et al 2023, McKnight et al 2017 | Tsanas et al 2016 | Spearman correlations | ASRM: Happy / MoodZoom: Negative | A-AA vs EM | Mania | 130.00 | 0.06 |  | Weak |
| Lewis et al 2023, McKnight et al 2017 | Tsanas et al 2016 | Spearman correlations | ASRM: Confident / MoodZoom: Negative | A-AA vs EM | Mania | 130.00 | 0.06 |  | Weak |
| Lewis et al 2023, McKnight et al 2017 | Tsanas et al 2016 | Spearman correlations | ASRM: Sleep / MoodZoom: Negative | A-AA vs EM | Mania | 130.00 | 0.18 |  | Weak |
| Lewis et al 2023, McKnight et al 2017 | Tsanas et al 2016 | Spearman correlations | ASRM: Talkative / MoodZoom: Negative | A-AA vs EM | Mania | 130.00 | 0.16 |  | Weak |
| Lewis et al 2023, McKnight et al 2017 | Tsanas et al 2016 | Spearman correlations | ASRM: Active / MoodZoom: Negative | A-AA vs EM | Mania | 130.00 | 0.12 |  | Weak |
| Lewis et al 2023, McKnight et al 2017 | Tsanas et al 2016 | Spearman correlations | ASRM: Happy / MoodZoom: Positive | A-AA vs EM | Mania | 130.00 | 0.26 |  | Weak |
| Lewis et al 2023, McKnight et al 2017 | Tsanas et al 2016 | Spearman correlations | ASRM: Confident / MoodZoom: Positive | A-AA vs EM | Mania | 130.00 | 0.26 |  | Weak |
| Lewis et al 2023, McKnight et al 2017 | Tsanas et al 2016 | Spearman correlations | ASRM: Sleep / MoodZoom: Positive | A-AA vs EM | Mania | 130.00 | 0.17 |  | Weak |
| Lewis et al 2023, McKnight et al 2017 | Tsanas et al 2016 | Spearman correlations | ASRM: Talkative / MoodZoom: Positive | A-AA vs EM | Mania | 130.00 | 0.21 |  | Weak |
| Lewis et al 2023, McKnight et al 2017 | Tsanas et al 2016 | Spearman correlations | ASRM: Active / MoodZoom: Positive | A-AA vs EM | Mania | 130.00 | 0.24 |  | Weak |
| Lewis et al 2023, McKnight et al 2017 | Tsanas et al 2016 | Spearman correlations | ASRM: Happy / MoodZoom: Irritability | A-AA vs EM | Mania | 130.00 | -0.06 |  | Weak |
| Lewis et al 2023, McKnight et al 2017 | Tsanas et al 2016 | Spearman correlations | ASRM: Confident / MoodZoom: Irritability | A-AA vs EM | Mania | 130.00 | -0.04 |  | Weak |
| Lewis et al 2023, McKnight et al 2017 | Tsanas et al 2016 | Spearman correlations | ASRM: Sleep / MoodZoom: Irritability | A-AA vs EM | Mania | 130.00 | -0.01 |  | Weak |
| Lewis et al 2023, McKnight et al 2017 | Tsanas et al 2016 | Spearman correlations | ASRM: Talkative / MoodZoom: Irritability | A-AA vs EM | Mania | 130.00 | -0.06 |  | Weak |
| Lewis et al 2023, McKnight et al 2017 | Tsanas et al 2016 | Spearman correlations | ASRM: Active / MoodZoom: Irritability | A-AA vs EM | Mania | 130.00 | -0.06 |  | Weak |
| Lewis et al 2023, McKnight et al 2017 | Tsanas et al 2016 | Spearman correlations | QIDS: Sleep / MoodZoom: Anxious | A-AA vs EM | Depression | 130.00 | 0.38 |  | Moderate |
| Lewis et al 2023, McKnight et al 2017 | Tsanas et al 2016 | Spearman correlations | QIDS: Sad / MoodZoom: Anxious | A-AA vs EM | Depression | 130.00 | 0.65 |  | Moderate |
| Lewis et al 2023, McKnight et al 2017 | Tsanas et al 2016 | Spearman correlations | QIDS: Appetite / MoodZoom: Anxious | A-AA vs EM | Depression | 130.00 | 0.46 |  | Moderate |
| Lewis et al 2023, McKnight et al 2017 | Tsanas et al 2016 | Spearman correlations | QIDS: Concentration / MoodZoom: Anxious | A-AA vs EM | Depression | 130.00 | 0.59 |  | Moderate |
| Lewis et al 2023, McKnight et al 2017 | Tsanas et al 2016 | Spearman correlations | QIDS: Self-view / MoodZoom: Anxious | A-AA vs EM | Depression | 130.00 | 0.59 |  | Moderate |
| Lewis et al 2023, McKnight et al 2017 | Tsanas et al 2016 | Spearman correlations | QIDS: Suicide / MoodZoom: Anxious | A-AA vs EM | Depression | 130.00 | 0.47 |  | Moderate |
| Lewis et al 2023, McKnight et al 2017 | Tsanas et al 2016 | Spearman correlations | QIDS: Interest / MoodZoom: Anxious | A-AA vs EM | Depression | 130.00 | 0.52 |  | Moderate |
| Lewis et al 2023, McKnight et al 2017 | Tsanas et al 2016 | Spearman correlations | QIDS: Energy / MoodZoom: Anxious | A-AA vs EM | Depression | 130.00 | 0.54 |  | Moderate |
| Lewis et al 2023, McKnight et al 2017 | Tsanas et al 2016 | Spearman correlations | QIDS: Restless / MoodZoom: Anxious | A-AA vs EM | Depression | 130.00 | 0.57 |  | Moderate |
| Lewis et al 2023, McKnight et al 2017 | Tsanas et al 2016 | Spearman correlations | QIDS: Sleep / MoodZoom: Elated | A-AA vs EM | Depression | 130.00 | -0.08 |  | Weak |
| Lewis et al 2023, McKnight et al 2017 | Tsanas et al 2016 | Spearman correlations | QIDS: Sad / MoodZoom: Elated | A-AA vs EM | Depression | 130.00 | -0.01 |  | Weak |
| Lewis et al 2023, McKnight et al 2017 | Tsanas et al 2016 | Spearman correlations | QIDS: Appetite / MoodZoom: Elated | A-AA vs EM | Depression | 130.00 | -0.02 |  | Weak |
| Lewis et al 2023, McKnight et al 2017 | Tsanas et al 2016 | Spearman correlations | QIDS: Concentration / MoodZoom: Elated | A-AA vs EM | Depression | 130.00 | -0.09 |  | Weak |
| Lewis et al 2023, McKnight et al 2017 | Tsanas et al 2016 | Spearman correlations | QIDS: Self-view / MoodZoom: Elated | A-AA vs EM | Depression | 130.00 | -0.03 |  | Weak |
| Lewis et al 2023, McKnight et al 2017 | Tsanas et al 2016 | Spearman correlations | QIDS: Suicide / MoodZoom: Elated | A-AA vs EM | Depression | 130.00 | -0.06 |  | Weak |
| Lewis et al 2023, McKnight et al 2017 | Tsanas et al 2016 | Spearman correlations | QIDS: Interest / MoodZoom: Elated | A-AA vs EM | Depression | 130.00 | -0.07 |  | Weak |
| Lewis et al 2023, McKnight et al 2017 | Tsanas et al 2016 | Spearman correlations | QIDS: Energy / MoodZoom: Elated | A-AA vs EM | Depression | 130.00 | -0.12 |  | Weak |
| Lewis et al 2023, McKnight et al 2017 | Tsanas et al 2016 | Spearman correlations | QIDS: Restless / MoodZoom: Elated | A-AA vs EM | Depression | 130.00 | -0.04 |  | Weak |
| Lewis et al 2023, McKnight et al 2017 | Tsanas et al 2016 | Spearman correlations | QIDS: Sleep / MoodZoom: Sad | A-AA vs EM | Depression | 130.00 | 0.33 |  | Moderate |
| Lewis et al 2023, McKnight et al 2017 | Tsanas et al 2016 | Spearman correlations | QIDS: Sad / MoodZoom: Sad | A-AA vs EM | Depression | 130.00 | 0.76 |  | Strong |
| Lewis et al 2023, McKnight et al 2017 | Tsanas et al 2016 | Spearman correlations | QIDS: Appetite / MoodZoom: Sad | A-AA vs EM | Depression | 130.00 | 0.39 |  | Moderate |
| Lewis et al 2023, McKnight et al 2017 | Tsanas et al 2016 | Spearman correlations | QIDS: Concentration / MoodZoom: Sad | A-AA vs EM | Depression | 130.00 | 0.56 |  | Moderate |
| Lewis et al 2023, McKnight et al 2017 | Tsanas et al 2016 | Spearman correlations | QIDS: Self-view / MoodZoom: Sad | A-AA vs EM | Depression | 130.00 | 0.63 |  | Moderate |
| Lewis et al 2023, McKnight et al 2017 | Tsanas et al 2016 | Spearman correlations | QIDS: Suicide / MoodZoom: Sad | A-AA vs EM | Depression | 130.00 | 0.56 |  | Moderate |
| Lewis et al 2023, McKnight et al 2017 | Tsanas et al 2016 | Spearman correlations | QIDS: Interest / MoodZoom: Sad | A-AA vs EM | Depression | 130.00 | 0.57 |  | Moderate |
| Lewis et al 2023, McKnight et al 2017 | Tsanas et al 2016 | Spearman correlations | QIDS: Energy / MoodZoom: Sad | A-AA vs EM | Depression | 130.00 | 0.55 |  | Moderate |
| Lewis et al 2023, McKnight et al 2017 | Tsanas et al 2016 | Spearman correlations | QIDS: Restless / MoodZoom: Sad | A-AA vs EM | Depression | 130.00 | 0.55 |  | Moderate |
| Lewis et al 2023, McKnight et al 2017 | Tsanas et al 2016 | Spearman correlations | QIDS: Sleep / MoodZoom: Angry | A-AA vs EM | Depression | 130.00 | 0.31 |  | Moderate |
| Lewis et al 2023, McKnight et al 2017 | Tsanas et al 2016 | Spearman correlations | QIDS: Sad / MoodZoom: Angry | A-AA vs EM | Depression | 130.00 | 0.55 |  | Moderate |
| Lewis et al 2023, McKnight et al 2017 | Tsanas et al 2016 | Spearman correlations | QIDS: Appetite / MoodZoom: Angry | A-AA vs EM | Depression | 130.00 | 0.35 |  | Moderate |
| Lewis et al 2023, McKnight et al 2017 | Tsanas et al 2016 | Spearman correlations | QIDS: Concentration / MoodZoom: Angry | A-AA vs EM | Depression | 130.00 | 0.46 |  | Moderate |
| Lewis et al 2023, McKnight et al 2017 | Tsanas et al 2016 | Spearman correlations | QIDS: Self-view / MoodZoom: Angry | A-AA vs EM | Depression | 130.00 | 0.45 |  | Moderate |
| Lewis et al 2023, McKnight et al 2017 | Tsanas et al 2016 | Spearman correlations | QIDS: Suicide / MoodZoom: Angry | A-AA vs EM | Depression | 130.00 | 0.41 |  | Moderate |
| Lewis et al 2023, McKnight et al 2017 | Tsanas et al 2016 | Spearman correlations | QIDS: Interest / MoodZoom: Angry | A-AA vs EM | Depression | 130.00 | 0.41 |  | Moderate |
| Lewis et al 2023, McKnight et al 2017 | Tsanas et al 2016 | Spearman correlations | QIDS: Energy / MoodZoom: Angry | A-AA vs EM | Depression | 130.00 | 0.39 |  | Moderate |
| Lewis et al 2023, McKnight et al 2017 | Tsanas et al 2016 | Spearman correlations | QIDS: Restless / MoodZoom: Angry | A-AA vs EM | Depression | 130.00 | 0.44 |  | Moderate |
| Lewis et al 2023, McKnight et al 2017 | Tsanas et al 2016 | Spearman correlations | QIDS: Sleep / MoodZoom: Irritable | A-AA vs EM | Depression | 130.00 | 0.34 |  | Moderate |
| Lewis et al 2023, McKnight et al 2017 | Tsanas et al 2016 | Spearman correlations | QIDS: Sad / MoodZoom: Irritable | A-AA vs EM | Depression | 130.00 | 0.53 |  | Moderate |
| Lewis et al 2023, McKnight et al 2017 | Tsanas et al 2016 | Spearman correlations | QIDS: Appetite / MoodZoom: Irritable | A-AA vs EM | Depression | 130.00 | 0.39 |  | Moderate |
| Lewis et al 2023, McKnight et al 2017 | Tsanas et al 2016 | Spearman correlations | QIDS: Concentration / MoodZoom: Irritable | A-AA vs EM | Depression | 130.00 | 0.49 |  | Moderate |
| Lewis et al 2023, McKnight et al 2017 | Tsanas et al 2016 | Spearman correlations | QIDS: Self-view / MoodZoom: Irritable | A-AA vs EM | Depression | 130.00 | 0.46 |  | Moderate |
| Lewis et al 2023, McKnight et al 2017 | Tsanas et al 2016 | Spearman correlations | QIDS: Suicide / MoodZoom: Irritable | A-AA vs EM | Depression | 130.00 | 0.39 |  | Moderate |
| Lewis et al 2023, McKnight et al 2017 | Tsanas et al 2016 | Spearman correlations | QIDS: Interest / MoodZoom: Irritable | A-AA vs EM | Depression | 130.00 | 0.43 |  | Moderate |
| Lewis et al 2023, McKnight et al 2017 | Tsanas et al 2016 | Spearman correlations | QIDS: Energy / MoodZoom: Irritable | A-AA vs EM | Depression | 130.00 | 0.42 |  | Moderate |
| Lewis et al 2023, McKnight et al 2017 | Tsanas et al 2016 | Spearman correlations | QIDS: Restless / MoodZoom: Irritable | A-AA vs EM | Depression | 130.00 | 0.49 |  | Moderate |
| Lewis et al 2023, McKnight et al 2017 | Tsanas et al 2016 | Spearman correlations | QIDS: Sleep / MoodZoom: Energetic | A-AA vs EM | Depression | 130.00 | -0.13 |  | Weak |
| Lewis et al 2023, McKnight et al 2017 | Tsanas et al 2016 | Spearman correlations | QIDS: Sad / MoodZoom: Energetic | A-AA vs EM | Depression | 130.00 | -0.16 |  | Weak |
| Lewis et al 2023, McKnight et al 2017 | Tsanas et al 2016 | Spearman correlations | QIDS: Appetite / MoodZoom: Energetic | A-AA vs EM | Depression | 130.00 | -0.17 |  | Weak |
| Lewis et al 2023, McKnight et al 2017 | Tsanas et al 2016 | Spearman correlations | QIDS: Concentration / MoodZoom: Energetic | A-AA vs EM | Depression | 130.00 | -0.23 |  | Weak |
| Lewis et al 2023, McKnight et al 2017 | Tsanas et al 2016 | Spearman correlations | QIDS: Self-view / MoodZoom: Energetic | A-AA vs EM | Depression | 130.00 | -0.18 |  | Weak |
| Lewis et al 2023, McKnight et al 2017 | Tsanas et al 2016 | Spearman correlations | QIDS: Suicide / MoodZoom: Energetic | A-AA vs EM | Depression | 130.00 | -0.17 |  | Weak |
| Lewis et al 2023, McKnight et al 2017 | Tsanas et al 2016 | Spearman correlations | QIDS: Interest / MoodZoom: Energetic | A-AA vs EM | Depression | 130.00 | -0.20 |  | Weak |
| Lewis et al 2023, McKnight et al 2017 | Tsanas et al 2016 | Spearman correlations | QIDS: Energy / MoodZoom: Energetic | A-AA vs EM | Depression | 130.00 | -0.27 |  | Weak |
| Lewis et al 2023, McKnight et al 2017 | Tsanas et al 2016 | Spearman correlations | QIDS: Restless / MoodZoom: Energetic | A-AA vs EM | Depression | 130.00 | -0.15 |  | Weak |
| Lewis et al 2023, McKnight et al 2017 | Tsanas et al 2016 | Spearman correlations | QIDS: Sleep / MoodZoom: Negative | A-AA vs EM | Depression | 130.00 | 0.39 |  | Moderate |
| Lewis et al 2023, McKnight et al 2017 | Tsanas et al 2016 | Spearman correlations | QIDS: Sad / MoodZoom: Negative | A-AA vs EM | Depression | 130.00 | 0.71 |  | Strong |
| Lewis et al 2023, McKnight et al 2017 | Tsanas et al 2016 | Spearman correlations | QIDS: Appetite / MoodZoom: Negative | A-AA vs EM | Depression | 130.00 | 0.46 |  | Moderate |
| Lewis et al 2023, McKnight et al 2017 | Tsanas et al 2016 | Spearman correlations | QIDS: Concentration / MoodZoom: Negative | A-AA vs EM | Depression | 130.00 | 0.61 |  | Moderate |
| Lewis et al 2023, McKnight et al 2017 | Tsanas et al 2016 | Spearman correlations | QIDS: Self-view / MoodZoom: Negative | A-AA vs EM | Depression | 130.00 | 0.62 |  | Moderate |
| Lewis et al 2023, McKnight et al 2017 | Tsanas et al 2016 | Spearman correlations | QIDS: Suicide / MoodZoom: Negative | A-AA vs EM | Depression | 130.00 | 0.53 |  | Moderate |
| Lewis et al 2023, McKnight et al 2017 | Tsanas et al 2016 | Spearman correlations | QIDS: Interest / MoodZoom: Negative | A-AA vs EM | Depression | 130.00 | 0.56 |  | Moderate |
| Lewis et al 2023, McKnight et al 2017 | Tsanas et al 2016 | Spearman correlations | QIDS: Energy / MoodZoom: Negative | A-AA vs EM | Depression | 130.00 | 0.57 |  | Moderate |
| Lewis et al 2023, McKnight et al 2017 | Tsanas et al 2016 | Spearman correlations | QIDS: Restless / MoodZoom: Negative | A-AA vs EM | Depression | 130.00 | 0.60 |  | Moderate |
| Lewis et al 2023, McKnight et al 2017 | Tsanas et al 2016 | Spearman correlations | QIDS: Sleep / MoodZoom: Positive | A-AA vs EM | Depression | 130.00 | 0.00 |  | Weak |
| Lewis et al 2023, McKnight et al 2017 | Tsanas et al 2016 | Spearman correlations | QIDS: Sad / MoodZoom: Positive | A-AA vs EM | Depression | 130.00 | 0.08 |  | Weak |
| Lewis et al 2023, McKnight et al 2017 | Tsanas et al 2016 | Spearman correlations | QIDS: Appetite / MoodZoom: Positive | A-AA vs EM | Depression | 130.00 | 0.02 |  | Weak |
| Lewis et al 2023, McKnight et al 2017 | Tsanas et al 2016 | Spearman correlations | QIDS: Concentration / MoodZoom: Positive | A-AA vs EM | Depression | 130.00 | -0.02 |  | Weak |
| Lewis et al 2023, McKnight et al 2017 | Tsanas et al 2016 | Spearman correlations | QIDS: Self-view / MoodZoom: Positive | A-AA vs EM | Depression | 130.00 | 0.04 |  | Weak |
| Lewis et al 2023, McKnight et al 2017 | Tsanas et al 2016 | Spearman correlations | QIDS: Suicide / MoodZoom: Positive | A-AA vs EM | Depression | 130.00 | 0.01 |  | Weak |
| Lewis et al 2023, McKnight et al 2017 | Tsanas et al 2016 | Spearman correlations | QIDS: Interest / MoodZoom: Positive | A-AA vs EM | Depression | 130.00 | 0.00 |  | Weak |
| Lewis et al 2023, McKnight et al 2017 | Tsanas et al 2016 | Spearman correlations | QIDS: Energy / MoodZoom: Positive | A-AA vs EM | Depression | 130.00 | -0.06 |  | Weak |
| Lewis et al 2023, McKnight et al 2017 | Tsanas et al 2016 | Spearman correlations | QIDS: Restless / MoodZoom: Positive | A-AA vs EM | Depression | 130.00 | 0.05 |  | Weak |
| Lewis et al 2023, McKnight et al 2017 | Tsanas et al 2016 | Spearman correlations | QIDS: Sleep / MoodZoom: Irritability | A-AA vs EM | Depression | 130.00 | -0.02 |  | Weak |
| Lewis et al 2023, McKnight et al 2017 | Tsanas et al 2016 | Spearman correlations | QIDS: Sad / MoodZoom: Irritability | A-AA vs EM | Depression | 130.00 | -0.30 |  | Moderate |
| Lewis et al 2023, McKnight et al 2017 | Tsanas et al 2016 | Spearman correlations | QIDS: Appetite / MoodZoom: Irritability | A-AA vs EM | Depression | 130.00 | -0.09 |  | Weak |
| Lewis et al 2023, McKnight et al 2017 | Tsanas et al 2016 | Spearman correlations | QIDS: Concentration / MoodZoom: Irritability | A-AA vs EM | Depression | 130.00 | -0.18 |  | Weak |
| Lewis et al 2023, McKnight et al 2017 | Tsanas et al 2016 | Spearman correlations | QIDS: Self-view / MoodZoom: Irritability | A-AA vs EM | Depression | 130.00 | -0.25 |  | Weak |
| Lewis et al 2023, McKnight et al 2017 | Tsanas et al 2016 | Spearman correlations | QIDS: Suicide / MoodZoom: Irritability | A-AA vs EM | Depression | 130.00 | -0.16 |  | Weak |
| Lewis et al 2023, McKnight et al 2017 | Tsanas et al 2016 | Spearman correlations | QIDS: Interest / MoodZoom: Irritability | A-AA vs EM | Depression | 130.00 | -0.18 |  | Weak |
| Lewis et al 2023, McKnight et al 2017 | Tsanas et al 2016 | Spearman correlations | QIDS: Energy / MoodZoom: Irritability | A-AA vs EM | Depression | 130.00 | -0.21 |  | Weak |
| Lewis et al 2023, McKnight et al 2017 | Tsanas et al 2016 | Spearman correlations | QIDS: Restless / MoodZoom: Irritability | A-AA vs EM | Depression | 130.00 | -0.15 |  | Weak |
| Lewis et al 2023, McKnight et al 2017 | Tsanas et al 2016 | Spearman correlations | GAD7: Nervous/Anxious / MoodZoom: Anxious | A-AA vs EM | Anxiety | 130.00 | 0.72 |  | Strong |
| Lewis et al 2023, McKnight et al 2017 | Tsanas et al 2016 | Spearman correlations | GAD7: Control worries/ MoodZoom: Anxious | A-AA vs EM | Anxiety | 130.00 | 0.67 |  | Moderate |
| Lewis et al 2023, McKnight et al 2017 | Tsanas et al 2016 | Spearman correlations | GAD7: Worried / MoodZoom: Anxious | A-AA vs EM | Anxiety | 130.00 | 0.69 |  | Moderate |
| Lewis et al 2023, McKnight et al 2017 | Tsanas et al 2016 | Spearman correlations | GAD7: Relaxed / MoodZoom: Anxious | A-AA vs EM | Anxiety | 130.00 | 0.68 |  | Moderate |
| Lewis et al 2023, McKnight et al 2017 | Tsanas et al 2016 | Spearman correlations | GAD7: Restless / MoodZoom: Anxious | A-AA vs EM | Anxiety | 130.00 | 0.54 |  | Moderate |
| Lewis et al 2023, McKnight et al 2017 | Tsanas et al 2016 | Spearman correlations | GAD7: Irritable / MoodZoom: Anxious | A-AA vs EM | Anxiety | 130.00 | 0.63 |  | Moderate |
| Lewis et al 2023, McKnight et al 2017 | Tsanas et al 2016 | Spearman correlations | GAD7: Afraid / MoodZoom: Anxious | A-AA vs EM | Anxiety | 130.00 | 0.67 |  | Moderate |
| Lewis et al 2023, McKnight et al 2017 | Tsanas et al 2016 | Spearman correlations | GAD7: Nervous/Anxious / MoodZoom: Elated | A-AA vs EM | Anxiety | 130.00 | 0.00 |  | Weak |
| Lewis et al 2023, McKnight et al 2017 | Tsanas et al 2016 | Spearman correlations | GAD7: Control worries/ MoodZoom: Elated | A-AA vs EM | Anxiety | 130.00 | 0.00 |  | Weak |
| Lewis et al 2023, McKnight et al 2017 | Tsanas et al 2016 | Spearman correlations | GAD7: Worried / MoodZoom: Elated | A-AA vs EM | Anxiety | 130.00 | 0.01 |  | Weak |
| Lewis et al 2023, McKnight et al 2017 | Tsanas et al 2016 | Spearman correlations | GAD7: Relaxed / MoodZoom: Elated | A-AA vs EM | Anxiety | 130.00 | -0.02 |  | Weak |
| Lewis et al 2023, McKnight et al 2017 | Tsanas et al 2016 | Spearman correlations | GAD7: Restless / MoodZoom: Elated | A-AA vs EM | Anxiety | 130.00 | 0.09 |  | Weak |
| Lewis et al 2023, McKnight et al 2017 | Tsanas et al 2016 | Spearman correlations | GAD7: Irritable / MoodZoom: Elated | A-AA vs EM | Anxiety | 130.00 | 0.07 |  | Weak |
| Lewis et al 2023, McKnight et al 2017 | Tsanas et al 2016 | Spearman correlations | GAD7: Afraid / MoodZoom: Elated | A-AA vs EM | Anxiety | 130.00 | -0.04 |  | Weak |
| Lewis et al 2023, McKnight et al 2017 | Tsanas et al 2016 | Spearman correlations | GAD7: Nervous/Anxious / MoodZoom: Sad | A-AA vs EM | Anxiety | 130.00 | 0.64 |  | Moderate |
| Lewis et al 2023, McKnight et al 2017 | Tsanas et al 2016 | Spearman correlations | GAD7: Control worries/ MoodZoom: Sad | A-AA vs EM | Anxiety | 130.00 | 0.66 |  | Moderate |
| Lewis et al 2023, McKnight et al 2017 | Tsanas et al 2016 | Spearman correlations | GAD7: Worried / MoodZoom: Sad | A-AA vs EM | Anxiety | 130.00 | 0.66 |  | Moderate |
| Lewis et al 2023, McKnight et al 2017 | Tsanas et al 2016 | Spearman correlations | GAD7: Relaxed / MoodZoom: Sad | A-AA vs EM | Anxiety | 130.00 | 0.62 |  | Moderate |
| Lewis et al 2023, McKnight et al 2017 | Tsanas et al 2016 | Spearman correlations | GAD7: Restless / MoodZoom: Sad | A-AA vs EM | Anxiety | 130.00 | 0.50 |  | Moderate |
| Lewis et al 2023, McKnight et al 2017 | Tsanas et al 2016 | Spearman correlations | GAD7: Irritable / MoodZoom: Sad | A-AA vs EM | Anxiety | 130.00 | 0.58 |  | Moderate |
| Lewis et al 2023, McKnight et al 2017 | Tsanas et al 2016 | Spearman correlations | GAD7: Afraid / MoodZoom: Sad | A-AA vs EM | Anxiety | 130.00 | 0.67 |  | Moderate |
| Lewis et al 2023, McKnight et al 2017 | Tsanas et al 2016 | Spearman correlations | GAD7: Nervous/Anxious / MoodZoom: Angry | A-AA vs EM | Anxiety | 130.00 | 0.53 |  | Moderate |
| Lewis et al 2023, McKnight et al 2017 | Tsanas et al 2016 | Spearman correlations | GAD7: Control worries/ MoodZoom: Angry | A-AA vs EM | Anxiety | 130.00 | 0.54 |  | Moderate |
| Lewis et al 2023, McKnight et al 2017 | Tsanas et al 2016 | Spearman correlations | GAD7: Worried / MoodZoom: Angry | A-AA vs EM | Anxiety | 130.00 | 0.54 |  | Moderate |
| Lewis et al 2023, McKnight et al 2017 | Tsanas et al 2016 | Spearman correlations | GAD7: Relaxed / MoodZoom: Angry | A-AA vs EM | Anxiety | 130.00 | 0.51 |  | Moderate |
| Lewis et al 2023, McKnight et al 2017 | Tsanas et al 2016 | Spearman correlations | GAD7: Restless / MoodZoom: Angry | A-AA vs EM | Anxiety | 130.00 | 0.44 |  | Moderate |
| Lewis et al 2023, McKnight et al 2017 | Tsanas et al 2016 | Spearman correlations | GAD7: Irritable / MoodZoom: Angry | A-AA vs EM | Anxiety | 130.00 | 0.61 |  | Moderate |
| Lewis et al 2023, McKnight et al 2017 | Tsanas et al 2016 | Spearman correlations | GAD7: Afraid / MoodZoom: Angry | A-AA vs EM | Anxiety | 130.00 | 0.54 |  | Moderate |
| Lewis et al 2023, McKnight et al 2017 | Tsanas et al 2016 | Spearman correlations | GAD7: Nervous/Anxious / MoodZoom: Irritable | A-AA vs EM | Anxiety | 130.00 | 0.55 |  | Moderate |
| Lewis et al 2023, McKnight et al 2017 | Tsanas et al 2016 | Spearman correlations | GAD7: Control worries/ MoodZoom: Irritable | A-AA vs EM | Anxiety | 130.00 | 0.53 |  | Moderate |
| Lewis et al 2023, McKnight et al 2017 | Tsanas et al 2016 | Spearman correlations | GAD7: Worried / MoodZoom: Irritable | A-AA vs EM | Anxiety | 130.00 | 0.54 |  | Moderate |
| Lewis et al 2023, McKnight et al 2017 | Tsanas et al 2016 | Spearman correlations | GAD7: Relaxed / MoodZoom: Irritable | A-AA vs EM | Anxiety | 130.00 | 0.55 |  | Moderate |
| Lewis et al 2023, McKnight et al 2017 | Tsanas et al 2016 | Spearman correlations | GAD7: Restless / MoodZoom: Irritable | A-AA vs EM | Anxiety | 130.00 | 0.45 |  | Moderate |
| Lewis et al 2023, McKnight et al 2017 | Tsanas et al 2016 | Spearman correlations | GAD7: Irritable / MoodZoom: Irritable | A-AA vs EM | Anxiety | 130.00 | 0.69 |  | Moderate |
| Lewis et al 2023, McKnight et al 2017 | Tsanas et al 2016 | Spearman correlations | GAD7: Afraid / MoodZoom: Irritable | A-AA vs EM | Anxiety | 130.00 | 0.54 |  | Moderate |
| Lewis et al 2023, McKnight et al 2017 | Tsanas et al 2016 | Spearman correlations | GAD7: Nervous/Anxious / MoodZoom: Energetic | A-AA vs EM | Anxiety | 130.00 | -0.16 |  | Weak |
| Lewis et al 2023, McKnight et al 2017 | Tsanas et al 2016 | Spearman correlations | GAD7: Control worries/ MoodZoom: Energetic | A-AA vs EM | Anxiety | 130.00 | -0.14 |  | Weak |
| Lewis et al 2023, McKnight et al 2017 | Tsanas et al 2016 | Spearman correlations | GAD7: Worried / MoodZoom: Energetic | A-AA vs EM | Anxiety | 130.00 | -0.13 |  | Weak |
| Lewis et al 2023, McKnight et al 2017 | Tsanas et al 2016 | Spearman correlations | GAD7: Relaxed / MoodZoom: Energetic | A-AA vs EM | Anxiety | 130.00 | -0.15 |  | Weak |
| Lewis et al 2023, McKnight et al 2017 | Tsanas et al 2016 | Spearman correlations | GAD7: Restless / MoodZoom: Energetic | A-AA vs EM | Anxiety | 130.00 | -0.04 |  | Weak |
| Lewis et al 2023, McKnight et al 2017 | Tsanas et al 2016 | Spearman correlations | GAD7: Irritable / MoodZoom: Energetic | A-AA vs EM | Anxiety | 130.00 | -0.12 |  | Weak |
| Lewis et al 2023, McKnight et al 2017 | Tsanas et al 2016 | Spearman correlations | GAD7: Afraid / MoodZoom: Energetic | A-AA vs EM | Anxiety | 130.00 | -0.17 |  | Weak |
| Lewis et al 2023, McKnight et al 2017 | Tsanas et al 2016 | Spearman correlations | GAD7: Nervous/Anxious / MoodZoom: Negative | A-AA vs EM | Anxiety | 130.00 | 0.69 |  | Moderate |
| Lewis et al 2023, McKnight et al 2017 | Tsanas et al 2016 | Spearman correlations | GAD7: Control worries/ MoodZoom: Negative | A-AA vs EM | Anxiety | 130.00 | 0.67 |  | Moderate |
| Lewis et al 2023, McKnight et al 2017 | Tsanas et al 2016 | Spearman correlations | GAD7: Worried / MoodZoom: Negative | A-AA vs EM | Anxiety | 130.00 | 0.67 |  | Moderate |
| Lewis et al 2023, McKnight et al 2017 | Tsanas et al 2016 | Spearman correlations | GAD7: Relaxed / MoodZoom: Negative | A-AA vs EM | Anxiety | 130.00 | 0.67 |  | Moderate |
| Lewis et al 2023, McKnight et al 2017 | Tsanas et al 2016 | Spearman correlations | GAD7: Restless / MoodZoom: Negative | A-AA vs EM | Anxiety | 130.00 | 0.54 |  | Moderate |
| Lewis et al 2023, McKnight et al 2017 | Tsanas et al 2016 | Spearman correlations | GAD7: Irritable / MoodZoom: Negative | A-AA vs EM | Anxiety | 130.00 | 0.67 |  | Moderate |
| Lewis et al 2023, McKnight et al 2017 | Tsanas et al 2016 | Spearman correlations | GAD7: Afraid / MoodZoom: Negative | A-AA vs EM | Anxiety | 130.00 | 0.68 |  | Moderate |
| Lewis et al 2023, McKnight et al 2017 | Tsanas et al 2016 | Spearman correlations | GAD7: Nervous/Anxious / MoodZoom: Positive | A-AA vs EM | Anxiety | 130.00 | 0.08 |  | Weak |
| Lewis et al 2023, McKnight et al 2017 | Tsanas et al 2016 | Spearman correlations | GAD7: Control worries/ MoodZoom: Positive | A-AA vs EM | Anxiety | 130.00 | 0.10 |  | Weak |
| Lewis et al 2023, McKnight et al 2017 | Tsanas et al 2016 | Spearman correlations | GAD7: Worried / MoodZoom: Positive | A-AA vs EM | Anxiety | 130.00 | 0.11 |  | Weak |
| Lewis et al 2023, McKnight et al 2017 | Tsanas et al 2016 | Spearman correlations | GAD7: Relaxed / MoodZoom: Positive | A-AA vs EM | Anxiety | 130.00 | 0.08 |  | Weak |
| Lewis et al 2023, McKnight et al 2017 | Tsanas et al 2016 | Spearman correlations | GAD7: Restless / MoodZoom: Positive | A-AA vs EM | Anxiety | 130.00 | 0.16 |  | Weak |
| Lewis et al 2023, McKnight et al 2017 | Tsanas et al 2016 | Spearman correlations | GAD7: Irritable / MoodZoom: Positive | A-AA vs EM | Anxiety | 130.00 | 0.16 |  | Weak |
| Lewis et al 2023, McKnight et al 2017 | Tsanas et al 2016 | Spearman correlations | GAD7: Afraid / MoodZoom: Positive | A-AA vs EM | Anxiety | 130.00 | 0.07 |  | Weak |
| Lewis et al 2023, McKnight et al 2017 | Tsanas et al 2016 | Spearman correlations | GAD7: Nervous/Anxious / MoodZoom: Irritability | A-AA vs EM | Anxiety | 130.00 | -0.25 |  | Weak |
| Lewis et al 2023, McKnight et al 2017 | Tsanas et al 2016 | Spearman correlations | GAD7: Control worries/ MoodZoom: Irritability | A-AA vs EM | Anxiety | 130.00 | -0.24 |  | Weak |
| Lewis et al 2023, McKnight et al 2017 | Tsanas et al 2016 | Spearman correlations | GAD7: Worried / MoodZoom: Irritability | A-AA vs EM | Anxiety | 130.00 | -0.25 |  | Weak |
| Lewis et al 2023, McKnight et al 2017 | Tsanas et al 2016 | Spearman correlations | GAD7: Relaxed / MoodZoom: Irritability | A-AA vs EM | Anxiety | 130.00 | -0.22 |  | Weak |
| Lewis et al 2023, McKnight et al 2017 | Tsanas et al 2016 | Spearman correlations | GAD7: Restless / MoodZoom: Irritability | A-AA vs EM | Anxiety | 130.00 | -0.15 |  | Weak |
| Lewis et al 2023, McKnight et al 2017 | Tsanas et al 2016 | Spearman correlations | GAD7: Irritable / MoodZoom: Irritability | A-AA vs EM | Anxiety | 130.00 | 0.00 |  | Weak |
| Lewis et al 2023, McKnight et al 2017 | Tsanas et al 2016 | Spearman correlations | GAD7: Afraid / MoodZoom: Irritability | A-AA vs EM | Anxiety | 130.00 | -0.20 |  | Weak |
| Lewis et al 2023, McKnight et al 2017 | Tsanas et al 2016 | Spearman correlations | EQ-5D / MoodZoom: Anxious | A-AA vs EM | Quality of life | 130.00 | -0.58 |  | Moderate |
| Lewis et al 2023, McKnight et al 2017 | Tsanas et al 2016 | Spearman correlations | EQ-5D / MoodZoom: Elated | A-AA vs EM | Quality of life | 130.00 | 0.15 |  | Weak |
| Lewis et al 2023, McKnight et al 2017 | Tsanas et al 2016 | Spearman correlations | EQ-5D / MoodZoom: Sad | A-AA vs EM | Quality of life | 130.00 | -0.55 |  | Moderate |
| Lewis et al 2023, McKnight et al 2017 | Tsanas et al 2016 | Spearman correlations | EQ-5D / MoodZoom: Angry | A-AA vs EM | Quality of life | 130.00 | -0.46 |  | Moderate |
| Lewis et al 2023, McKnight et al 2017 | Tsanas et al 2016 | Spearman correlations | EQ-5D / MoodZoom: Irritable | A-AA vs EM | Quality of life | 130.00 | -0.50 |  | Moderate |
| Lewis et al 2023, McKnight et al 2017 | Tsanas et al 2016 | Spearman correlations | EQ-5D / MoodZoom: Energetic | A-AA vs EM | Quality of life | 130.00 | 0.37 |  | Moderate |
| Lewis et al 2023, McKnight et al 2017 | Tsanas et al 2016 | Spearman correlations | EQ-5D / MoodZoom: Negative | A-AA vs EM | Quality of life | 130.00 | -0.63 |  | Moderate |
| Lewis et al 2023, McKnight et al 2017 | Tsanas et al 2016 | Spearman correlations | EQ-5D / MoodZoom: Positive | A-AA vs EM | Quality of life | 130.00 | 0.11 |  | Weak |
| Lewis et al 2023, McKnight et al 2017 | Tsanas et al 2016 | Spearman correlations | EQ-5D / MoodZoom: Irritability | A-AA vs EM | Quality of life | 130.00 | 0.09 |  | Weak |
| Lewis et al 2023, McKnight et al 2017 | Tsanas et al 2016 | Spearman correlations | ASRM / MoodZoom: Anxious | A-AA vs EM | Mania | 130.00 | 0.19 |  | Weak |
| Lewis et al 2023, McKnight et al 2017 | Tsanas et al 2016 | Spearman correlations | ASRM / MoodZoom: Elated | A-AA vs EM | Mania | 130.00 | 0.26 |  | Weak |
| Lewis et al 2023, McKnight et al 2017 | Tsanas et al 2016 | Spearman correlations | ASRM / MoodZoom: Sad | A-AA vs EM | Mania | 130.00 | 0.16 |  | Weak |
| Lewis et al 2023, McKnight et al 2017 | Tsanas et al 2016 | Spearman correlations | ASRM / MoodZoom: Angry | A-AA vs EM | Mania | 130.00 | 0.17 |  | Weak |
| Lewis et al 2023, McKnight et al 2017 | Tsanas et al 2016 | Spearman correlations | ASRM / MoodZoom: Irritable | A-AA vs EM | Mania | 130.00 | 0.22 |  | Weak |
| Lewis et al 2023, McKnight et al 2017 | Tsanas et al 2016 | Spearman correlations | ASRM / MoodZoom: Energetic | A-AA vs EM | Mania | 130.00 | 0.17 |  | Weak |
| Lewis et al 2023, McKnight et al 2017 | Tsanas et al 2016 | Spearman correlations | ASRM / MoodZoom: Negative | A-AA vs EM | Mania | 130.00 | 0.17 |  | Weak |
| Lewis et al 2023, McKnight et al 2017 | Tsanas et al 2016 | Spearman correlations | ASRM / MoodZoom: Positive | A-AA vs EM | Mania | 130.00 | 0.27 |  | Weak |
| Lewis et al 2023, McKnight et al 2017 | Tsanas et al 2016 | Spearman correlations | ASRM / MoodZoom: Irritability | A-AA vs EM | Mania | 130.00 | -0.07 |  | Weak |
| Lewis et al 2023, McKnight et al 2017 | Tsanas et al 2016 | Spearman correlations | QIDS / MoodZoom: Anxious | A-AA vs EM | Depression | 130.00 | 0.67 |  | Moderate |
| Lewis et al 2023, McKnight et al 2017 | Tsanas et al 2016 | Spearman correlations | QIDS / MoodZoom: Elated | A-AA vs EM | Depression | 130.00 | -0.05 |  | Weak |
| Lewis et al 2023, McKnight et al 2017 | Tsanas et al 2016 | Spearman correlations | QIDS / MoodZoom: Sad | A-AA vs EM | Depression | 130.00 | 0.69 |  | Moderate |
| Lewis et al 2023, McKnight et al 2017 | Tsanas et al 2016 | Spearman correlations | QIDS / MoodZoom: Angry | A-AA vs EM | Depression | 130.00 | 0.53 |  | Moderate |
| Lewis et al 2023, McKnight et al 2017 | Tsanas et al 2016 | Spearman correlations | QIDS / MoodZoom: Irritable | A-AA vs EM | Depression | 130.00 | 0.56 |  | Moderate |
| Lewis et al 2023, McKnight et al 2017 | Tsanas et al 2016 | Spearman correlations | QIDS / MoodZoom: Energetic | A-AA vs EM | Depression | 130.00 | -0.22 |  | Weak |
| Lewis et al 2023, McKnight et al 2017 | Tsanas et al 2016 | Spearman correlations | QIDS / MoodZoom: Negative | A-AA vs EM | Depression | 130.00 | 0.71 |  | Strong |
| Lewis et al 2023, McKnight et al 2017 | Tsanas et al 2016 | Spearman correlations | QIDS / MoodZoom: Positive | A-AA vs EM | Depression | 130.00 | 0.03 |  | Weak |
| Lewis et al 2023, McKnight et al 2017 | Tsanas et al 2016 | Spearman correlations | QIDS / MoodZoom: Irritability | A-AA vs EM | Depression | 130.00 | -0.23 |  | Weak |
| Lewis et al 2023, McKnight et al 2017 | Tsanas et al 2016 | Spearman correlations | GAD-7 / MoodZoom: Anxious | A-AA vs EM | Anxiety | 130.00 | 0.77 |  | Strong |
| Lewis et al 2023, McKnight et al 2017 | Tsanas et al 2016 | Spearman correlations | GAD-7 / MoodZoom: Elated | A-AA vs EM | Anxiety | 130.00 | 0.03 |  | Weak |
| Lewis et al 2023, McKnight et al 2017 | Tsanas et al 2016 | Spearman correlations | GAD-7 / MoodZoom: Sad | A-AA vs EM | Anxiety | 130.00 | 0.72 |  | Strong |
| Lewis et al 2023, McKnight et al 2017 | Tsanas et al 2016 | Spearman correlations | GAD-7 / MoodZoom: Angry | A-AA vs EM | Anxiety | 130.00 | 0.61 |  | Moderate |
| Lewis et al 2023, McKnight et al 2017 | Tsanas et al 2016 | Spearman correlations | GAD-7 / MoodZoom: Irritable | A-AA vs EM | Anxiety | 130.00 | 0.65 |  | Moderate |
| Lewis et al 2023, McKnight et al 2017 | Tsanas et al 2016 | Spearman correlations | GAD-7 / MoodZoom: Energetic | A-AA vs EM | Anxiety | 130.00 | -0.15 |  | Weak |
| Lewis et al 2023, McKnight et al 2017 | Tsanas et al 2016 | Spearman correlations | GAD-7 / MoodZoom: Negative | A-AA vs EM | Anxiety | 130.00 | 0.77 |  | Strong |
| Lewis et al 2023, McKnight et al 2017 | Tsanas et al 2016 | Spearman correlations | GAD-7 / MoodZoom: Positive | A-AA vs EM | Anxiety | 130.00 | 0.13 |  | Weak |
| Lewis et al 2023, McKnight et al 2017 | Tsanas et al 2016 | Spearman correlations | GAD-7 / MoodZoom: Irritability | A-AA vs EM | Anxiety | 130.00 | -0.23 |  | Weak |
| **A-AA: Active Ambulatory Assessment, P-AA: Passive Ambulatory Assessment, EM: Established Measures** | | | | | | | | | |

| **Supplementary Table 3: Performance data for relevant studies - confusion matrices** | | | | | | | | | | |
| --- | --- | --- | --- | --- | --- | --- | --- | --- | --- | --- |
| Original study | Validation‎ study | Statistic | Comparison | Classification | Mood state | n | TN | FN | TP | FP |
| Anyz et al 2021 | Anyz et al 2021 - https://mental.jmir.org/2021/8/e26348/ | TN, FN, TP, FP | ASERT / YMRS | A-AA vs EM | Mania | 191.00 | 155.00 | 7.00 | 12.00 | 17.00 |
| Anyz et al 2021 | Anyz et al 2021 | TN, FN, TP, FP | ASERT / MADRS | A-AA vs EM | Depression | 336.00 | 250.00 | 12.00 | 47.00 | 27.00 |
| Arribas et al 2018 | Palmius et al 2017 - https://pubmed.ncbi.nlm.nih.gov/28113247/ | TN, FN, TP, FP | Performance metrics of the leave-one-participant-out classifier trained with the five features | A-AA vs P-AA | Depression | 22.00 | 12008.00 | 556.00 | 3744.00 | 2292.00 |
| Arribas et al 2018 | Arribas et al 2018 - https://arxiv.org/pdf/1707.07124 | TN, FN, TP, FP | Diagnostic performance of model: EUPD | A-AA vs EM | EUPD | 220.00 | 150.00 | 18.00 | 37.00 | 15.00 |
| Arribas et al 2018 | Arribas et al 2018 | TN, FN, TP, FP | Diagnostic performance of model: Healthy controls | A-AA vs EM | HC | 220.00 | 124.00 | 15.00 | 68.00 | 13.00 |
| Arribas et al 2018 | Arribas et al 2018 | TN, FN, TP, FP | Diagnostic performance of model: Bipolar disorder | A-AA vs EM | Overall Mood | 220.00 | 110.00 | 23.00 | 59.00 | 28.00 |
| Arribas et al 2018 | Palmius et al 2017 - https://pubmed.ncbi.nlm.nih.gov/28113247/ | TN, FN, TP, FP | Performance metrics of the leave-one-participant-out classifier trained with the five features | A-AA vs P-AA | Depression | 22.00 | 12008.00 | 556.00 | 3744.00 | 2292.00 |
| Arribas et al 2018 | Wu et al 2022 - https://pubmed.ncbi.nlm.nih.gov/36395144/ | TN, FN, TP, FP | Diagnostic performance of MRLSM model: EUPD | A-AA vs EM | EUPD | 139 | 77 | 7 | 27 | 11 |
| Arribas et al 2018 | Wu et al 2022 | TN, FN, TP, FP | Diagnostic performance of MRLSM model: Healthy controls | A-AA vs EM | HC | 139 | 60 | 8 | 44 | 10 |
| Arribas et al 2018 | Wu et al 2022 | TN, FN, TP, FP | Diagnostic performance of MRLSM model: Bipolar disorder | A-AA vs EM | Overall Mood | 139 | 71 | 20 | 33 | 14 |
| Arribas et al 2018 | Wu et al 2022 | TN, FN, TP, FP | Diagnostic performance of naive model: EUPD | A-AA vs EM | EUPD | 139 | 68 | 13 | 21 | 10 |
| Arribas et al 2018 | Wu et al 2022 | TN, FN, TP, FP | Diagnostic performance of naive model: Healthy controls | A-AA vs EM | HC | 139 | 49 | 12 | 40 | 16 |
| Arribas et al 2018 | Wu et al 2022 | TN, FN, TP, FP | Diagnostic performance of naive model: Bipolar disorder | A-AA vs EM | Overall Mood | 139 | 61 | 25 | 28 | 24 |
| Arribas et al 2018 | Wu et al 2022 | TN, FN, TP, FP | Diagnostic performance of KNN model: EUPD | A-AA vs EM | EUPD | 139 | 75 | 11 | 23 | 10 |
| Arribas et al 2018 | Wu et al 2022 | TN, FN, TP, FP | Diagnostic performance of KNN model: Healthy controls | A-AA vs EM | HC | 139 | 55 | 9 | 43 | 11 |
| Arribas et al 2018 | Wu et al 2022 | TN, FN, TP, FP | Diagnostic performance of KNN model: Bipolar disorder | A-AA vs EM | Overall Mood | 139 | 66 | 21 | 32 | 20 |
| Stanislaus et al 2020 | Faurholt-Jepsen et al 2019 - https://pubmed.ncbi.nlm.nih.gov/30387368/ | TN, FN, TP, FP | BD overall / Healthy Controls | P-AA DC | Overall Mood | 66.00 | 14.00 | 2.00 | 27.00 | 23.00 |
| Stanislaus et al 2020 | Faurholt-Jepsen et al 2019 | TN, FN, TP, FP | Euthmyic state / Healthy Controls | P-AA DC | Euthymia | 66.00 | 21.00 | 3.00 | 26.00 | 16.00 |
| Stanislaus et al 2020 | Faurholt-Jepsen et al 2019 | TN, FN, TP, FP | Depressive state / Healthy Controls | P-AA DC | Depression | 66.00 | 19.00 | 6.00 | 23.00 | 18.00 |
| Stanislaus et al 2020 | Faurholt-Jepsen et al 2019 | TN, FN, TP, FP | Manic state / Healthy Controls | P-AA DC | Mania | 66.00 | 33.00 | 15.00 | 14.00 | 4.00 |
| Stanislaus et al 2020 | Faurholt-Jepsen et al 2019 | TN, FN, TP, FP | Depressive state / Euthymic state | P-AA DC | Depression | 66.00 | 25.00 | 19.00 | 10.00 | 12.00 |
| Stanislaus et al 2020 | Faurholt-Jepsen et al 2019 | TN, FN, TP, FP | Manic state / Euthymic state | P-AA DC | Mania | 66.00 | 34.00 | 18.00 | 11.00 | 3.00 |
| Stanislaus et al 2020 | Faurholt-Jepsen et al 2019 | TN, FN, TP, FP | UD / BD overall | P-AA DC | Depression vs BD | 140.00 | 49.00 | 19.00 | 46.00 | 26.00 |
| Stanislaus et al 2020 | Faurholt-Jepsen et al 2019 | TN, FN, TP, FP | UD euthymic state / BD euthymic state | P-AA DC | Depression vs BD - euthymia | 140.00 | 49.00 | 14.00 | 51.00 | 26.00 |
| Stanislaus et al 2020 | Faurholt-Jepsen et al 2019 | TN, FN, TP, FP | UD depressive state / BD depressive state | P-AA DC | Depression vs BD - depression | 140.00 | 58.00 | 19.00 | 46.00 | 17.00 |
| Bauer et al 2023 | Bauer et al 2008 - https://pubmed.ncbi.nlm.nih.gov/18423616/ | TN, FN, TP, FP | Agreement between YMRS and ChronoRecord for measuring hypomania or mania - outpatient and inpatient data | A-AA vs EM | Hypomania & Mania | 340.00 | 268.00 | 7.00 | 52.00 | 13.00 |
| Bauer et al 2023 | Bauer et al 2008 | TN, FN, TP, FP | Agreement between YMRS and ChronoRecord for measuring hypomania or mania - inpatient data | A-AA vs EM | Hypomania & Mania | 57.00 | 8.00 | 6.00 | 41.00 | 2.00 |
| Bauer et al 2023 | Bauer et al 2008 | TN, FN, TP, FP | Agreement between YMRS and ChronoRecord for measuring mania - outpatient and inpatient data | A-AA vs EM | Mania | 340.00 | 307.00 | 7.00 | 18.00 | 8.00 |
| Bauer et al 2023 | Bauer et al 2008 | TN, FN, TP, FP | Agreement between YMRS and ChronoRecord for measuring mania - inpatient data | A-AA vs EM | Mania | 57.00 | 27.00 | 7.00 | 17.00 | 6.00 |
| O'Rouke et al 2021 | Osher et al 2020 - https://pubmed.ncbi.nlm.nih.gov/32035589/ | TN, FN, TP, FP | Clinical assessment of depression / BDSX | A-AA vs EM | Depression | 60.00 | 39.00 | 1.00 | 7.00 | 12.00 |
| O'Rouke et al 2021 | Osher et al 2020 | TN, FN, TP, FP | Clinical assessment of hypo/mania / BDSX | A-AA vs EM | Mania | 60.00 | 47.00 | 3.00 | 4.00 | 5.00 |
| Schneider et al 2022 - https://pubmed.ncbi.nlm.nih.gov/32883376/ | Schneider et al 2022 | TN, FN, TP, FP | Random forest classifier model results - model based on actigraphy features | P-AA DC | Overall Mood | 50.00 | 23.00 | 4.00 | 21.00 | 2.00 |
| Schneider et al 2022 | Schneider et al 2022 | TN, FN, TP, FP | Random forest classifier model results - model based on variance features | P-AA DC | Overall Mood | 50.00 | 20.00 | 6.00 | 19.00 | 5.00 |
| Schneider et al 2022 | Schneider et al 2022 | TN, FN, TP, FP | Random forest classifier model results - model based on employment independent features | P-AA DC | Overall Mood | 50.00 | 20.00 | 6.00 | 19.00 | 5.00 |
| **A-AA: Active Ambulatory Assessment, P-AA: Passive Ambulatory Assessment, EM: Established Measures, TN: True Negative, FN: False Negative, TP: True Positive, FP: False Positive, DC: direct comparison** | | | | | | | | | | |

| **Supplementary table 4: risk of bias assessments for included studies** | | | | | | | | |
| --- | --- | --- | --- | --- | --- | --- | --- | --- |
| **Study** | **Risk of bias criteria for non-randomised studies** | | | | | | | |
|  | **Confounding bias** | **Selection of participants into the study** | **Classification of interventions** | **Deviation from intended intervention** | **Missing data** | **Measurement of outcomes** | **Selection of reported result** | **Total no of low risk domains** |
| Anyz et al 2021 | Low risk | Unclear | Low risk | Unclear | Unclear | Unclear | Low risk | 3 |
| Hidalgo-Mazzei et al 2016 | Low risk | Low risk | Low risk | Low risk | Low risk | High risk | Low risk | 6 |
| Hidalgo-Mazzei et al 2018 | Low risk | Low risk | Low risk | High risk | High risk | High risk | Low risk | 4 |
| Garcia-Estela et al 2022 | Low risk | Low risk | Low risk | High risk | High risk | High risk | Low risk | 4 |
| Bauer et al 2023 | Low risk | Unclear | Low risk | Low risk | Low risk | High risk | Low risk | 5 |
| Bos et al 2022 | Low risk | Low risk | Low risk | Low risk | Low risk | High risk | Low risk | 6 |
| Bowden et al 2021 | Low risk | Unclear | Low risk | Low risk | Low risk | High risk | Low risk | 5 |
| Dominiak et al 2022 | Low risk | Low risk | Low risk | Low risk | Low risk | High risk | Low risk | 6 |
| Emden et al 2021 | Low risk | Low risk | Low risk | High risk | High risk | High risk | Low risk | 4 |
| Stanislaus et al 2020 | Low risk | Unclear | Low risk | Low risk | Low risk | Low risk | Low risk | 6 |
| Lee et al 2022 | Low risk | Unclear | Low risk | High risk | High risk | High risk | Low risk | 5 |
| Born et al 2014 | Low risk | High risk | Low risk | Unclear | Unclear | High risk | Low risk | 5 |
| Lieberman et al 2011 | High risk | High risk | Low risk | Unclear | Unclear | High risk | Low risk | 2 |
| Kupka et al 2005 | Low risk | Unclear | Low risk | Unclear | Unclear | High risk | Low risk | 3 |
| O’Rourke et al 2021 | Low risk | High risk | Low risk | High risk | Unclear | High risk | Low risk | 3 |
| Tseng et al 2022 | Low risk | Unclear | Low risk | High risk | High risk | High risk | Low risk | 5 |
| Ebner-Priemer et al 2020 | Low risk | Low risk | Low risk | Low risk | Low risk | High risk | Low risk | 6 |
| Gideon et al 2016 | Low risk | Unclear | Low risk | Unclear | Unclear | High risk | Low risk | 3 |
| Schneider et al 2022 | Low risk | Unclear | Low risk | Low risk | Low risk | High risk | Low risk | 6 |
| Scharer et al 2015 | Low risk | Unclear | Low risk | Unclear | Unclear | High risk | Low risk | 3 |
| van den Heuvel et al 2018 | Low risk | Low risk | Low risk | High risk | High risk | High risk | Low risk | 4 |
| Arribas et al 2018 | Low risk | Unclear | Low risk | High risk | High risk | High risk | Low risk | 3 |
| Lewis et al 2023 | Low risk | Unclear | Low risk | Low risk | Low risk | Low risk | Low risk | 6 |
| McKnight et al 2017 | Low risk | Unclear | Low risk | Low risk | Low risk | High risk | Low risk | 5 |
| Ortiz et al 2023 | Low risk | Unclear | Low risk | Unclear | Unclear | High risk | Low risk | 3 |
| **Study** | **Risk of bias criteria for randomised studies** | | | | | | | |
|  | **Random sequence generation** | **Allocation concealment** | **Blinding of participants and personnel** | **Blinding of outcome assessment** | **Incomplete outcome data** | **Selective reporting** | **Other sources of bias** | **Total no of low risk domains** |
| Bilderbeck et al 2016 | Low risk | Low risk | High risk | High risk | Low risk | Low risk | Low risk | 5 |
| Castle et al. 2010 | Low risk | Unclear | High risk | Unclear | Low risk | Low risk | Low risk | 4 |
| Denicoff et al 2002 | Unclear | High risk | High risk | Low risk | Low risk | Low risk | Low risk | 4 |
| Depp et al 2012 | Unclear | Low risk | Low risk | Low risk | Low risk | Low risk | Low risk | 6 |
| Faurholt-Jepsen et al 2015 | Low risk | Low risk | High risk | Unclear | Low risk | Low risk | Low risk | 6 |
| Faurholt-Jepsen et al 2019 | Low risk | Low risk | High risk | Low risk | Low risk | Low risk | Low risk | 6 |
| Faurholt-Jepsen et al 2020 | Low risk | Low risk | High risk | Low risk | Low risk | Low risk | Low risk | 6 |
| Gliddon et al. 2018 | Low risk | Low risk | High risk | Low risk | Low risk | Low risk | Low risk | 6 |
| Goldberg et al. 2006 | Unclear | Unclear | Low risk | Low risk | Unclear | Low risk | Low risk | 4 |
| Goulding et al 2022 | Low risk | Low risk | High risk | Low risk | Low risk | Low risk | Unclear | 6 |
| Langosch et al 2008 | Unclear | High risk | High risk | High risk | Low risk | Low risk | Low risk | 3 |
| Lauder et al. 2015 | Low risk | Low risk | Unclear | Low risk | High risk | Low risk | Unclear | 6 |
| Leverich et al 2006 | Unclear | Low risk | Unclear | Low risk | High risk | High risk | Low risk | 3 |
| Lieberman et al 2010 | Unclear | Unclear | Unclear | High risk | Low risk | Low risk | Low risk | 3 |
| Pahwa et al 2023 | Low risk | High risk | High risk | High risk | Low risk | Low risk | Low risk | 4 |
| Petzold et al. 2019 | Low risk | Low risk | High risk | Low risk | Low risk | Low risk | Low risk | 6 |
| Van den Berg et al 2023 | Unclear | Unclear | High risk | Low risk | Low risk | Low risk | Low risk | 4 |

Supplementary Information 1: Search strategy with PRISMA flowchart and PRISMA checklist

Search performed 3/3/23. The search strategy was trialled on one database first and then refined subsequently. The search results were uploaded to Rayyan (105). Search terms were determined based on discussion between researchers, previous reviews and consultation with specialist librarians. The search was performed from inception to 3/3/23. The search was updated on 28/10/24.

**Number of abstracts original search 3/3/23:**

Medline: 2984

Embase: 4827

PsychINFO: 3346

SCOPUS: 2321

IEE Xplore: 615

Proquest dissertations and theses global: 2697

Proquest SciTech Collection: 3489

Total: 20,279

Full text review: 565

**Number of abstracts of updated search 3/3/24 – 28/10/24:**

Medline: 494

Embase: 920

PsychINFO: 364

SCOPUS: 1088

IEE Xplore: 99

Proquest dissertations and theses global: 0

Proquest SciTech Collection: 469

Total prior to deduplication: 3236

Auto-deduplicated: 1119

Total: 2117

Full text review: 193

**Published literature:**

| **OVID Medline** | |
| --- | --- |
| 1 | exp bipolar disorder/ OR exp depression OR exp mania/ |
| 2 | (((bipolar or bi polar) adj5 (disorder$ or depress$)) or ((cyclothymi$ or rapid or ultradian) adj5 cycl$) or hypomani$ or mania$ or manic$ or mixed episode$ or rcbd).mp |
| 3 | ('Depressive Disorder' OR 'Depression' OR 'dysthymi*' OR 'affective disorder' OR 'affective disorders' OR 'mood disorder' OR 'mood disorders' OR 'depression*' OR 'depressive*' OR 'dysthymic disorder').mp |
| 4 | 1 OR 2 OR 3 |
| 5 | ('self monitor*' or 'self assess*' or 'self manag*' or 'self record*' or 'self surveillance' or 'patient* monitor*' or 'measurement technolog*' or 'telemonitor*' or 'remote monitor*' or 'passive monitor*' or 'active monitor*' or 'mood track*' or 'mood monitor*' or 'experience sampl*' or 'ecological momentary assessment').mp |
| 6 | 4 adj10 5 |

<https://ovidsp.ovid.com/ovidweb.cgi?T=JS&NEWS=N&PAGE=main&SHAREDSEARCHID=10Q4IjupCc3HoHvVXInK959r2tcDy9vTlJlQsifUVfSVSyNrczwzGqVCqql3svtpo>

| **OVID EMBASE** | |
| --- | --- |
| 1 | bipolar disorder/ or bipolar depression/ or bipolar I disorder/ |
| 2 | depression assessment/ or treatment resistant depression/ or minor depression/ or chronic depression/ or postnatal depression/ or atypical depression/ or antenatal depression/ or adolescent depression/ or "mixed mania and depression"/ or post-stroke depression/ or endogenous depression/ or major depression/ or recurrent brief depression/ or depression/ or bipolar depression/ or perinatal depression/ or agitated depression/ or organic depression/ |
| 3 | "mixed mania and depression"/ or mania/ or bipolar mania/ |
| 4 | (((bipolar or bi polar) adj5 (disorder$ or depress$)) or ((cyclothymi$ or rapid or ultradian) adj5 cycl$) or hypomani$ or mania$ or manic$ or mixed episode$ or rcbd).mp. |
| 5 | ('Depressive Disorder' or 'Depression' or 'dysthymi*' or 'affective disorder' or 'affective disorders' or 'mood disorder' or 'mood disorders' or 'depression*' or 'depressive*' or 'dysthymic disorder').mp. |
| 6 | 1 OR 2 OR 3 OR 4 OR 5 |
| 7 | ('self monitor*' or 'self assess*' or 'self manag*' or 'self record*' or 'self surveillance' or 'patient* monitor*' or 'measurement technolog*' or 'telemonitor*' or 'remote monitor*' or 'passive monitor*' or 'active monitor*' or 'mood track*' or 'mood monitor*' or 'experience sampl*' or 'ecological momentary assessment').mp. |
| 8 | 6 adj10 7 |

https://ovidsp.ovid.com/ovidweb.cgi?T=JS&NEWS=N&PAGE=main&SHAREDSEARCHID=2Ofrc9VijRp6L40USOJFeEn3I1bHOmCW8O1Hzfz13xklneo3jW1767QyyDQMHnVDj

| **OVID PsychINFO** | |
| --- | --- |
| 1 | Bipolar Disorder/ or Bipolar II Disorder/ or Bipolar I Disorder/ or Mania/ |
| 2 | Major Depression/ or Endogenous Depression/ or Postpartum Depression/ or Recurrent Depression/ or "Depression (Emotion)"/ or Reactive Depression/ or Late Life Depression/ or Atypical Depression/ or Treatment Resistant Depression/ or "Long-term Depression (Neuronal)".mp. |
| 3 | (((bipolar or bi polar) adj5 (disorder$ or depress$)) or ((cyclothymi$ or rapid or ultradian) adj5 cycl$) or hypomani$ or mania$ or manic$ or mixed episode$ or rcbd).mp |
| 4 | 'Depressive Disorder' OR 'Depression' OR 'dysthymi*' OR 'affective disorder' OR 'affective disorders' OR 'mood disorder' OR 'mood disorders' OR 'depression*' OR 'depressive*' OR 'dysthymic disorder' |
| 5 | 1 OR 2 OR 3 OR 4 |
| 6 | ('self monitor*' or 'self assess*' or 'self manag*' or 'self record*' or 'self surveillance' or 'patient* monitor*' or 'measurement technolog*' or 'telemonitor*' or 'remote monitor*' or 'passive monitor*' or 'active monitor*' or 'mood track*' or 'mood monitor*' or 'experience sampl*' or 'ecological momentary assessment') |
| 7 | 5 adj10 6 |

https://ovidsp.ovid.com/ovidweb.cgi?T=JS&NEWS=N&PAGE=main&SHAREDSEARCHID=7WPhKe8RR9Athylx2jCCPdAkgbQlgcrdVpxl6NGPhskh73E8wr3X16vfACAP9Q54Y

**SCOPUS:**

TITLE-ABS-KEY({self monitor*} OR {self-monitor*} OR {self-assess*} OR {self manag*} OR {self-manag*} OR {self record*} OR {self-record*} OR {self surveillance} OR {self-surveillance} OR {patient* monitor*} OR {patient*-monitor*} OR {measurement technolog*} OR {measurement-technolog*} OR {telemonitor*} OR {remote monitor*} OR {remote-monitor*} OR {passive monitor*} OR {passive-monitor*} OR {active monitor*} OR {active-monitor*} OR {mood track*} OR {mood-track*} OR {mood monitor*} OR {mood-monitor*} OR {experience sampl*} OR {ecological momentary assessment}) W/10 ({Bipolar disorder*} OR {Manic depress*} OR {Manic-depress*} OR {Bipolar affective psychos*} OR {Bipolar depress*} OR {Manic disorder*} OR (106) OR {depressive disorder*} OR {major depressive disorder*} OR (106) OR {affective disorder*} OR {mood disorder*})

**IEE XPLORE:**

('self monitor' OR 'self monitoring' OR 'self assess' OR 'self assessment' OR 'self manage' OR 'self management' OR 'self record' OR 'self recording' OR 'self surveillance' OR 'patient monitor' OR 'patient monitoring' OR 'measurement technology' OR 'telemonitor' OR 'telemonitoring' OR 'remote monitor' OR 'remote monitoring' OR 'passive monitor' OR 'passive monitoring' OR 'active monitor*' OR 'mood track*' OR 'mood monitor*' OR 'experience sample' OR 'experience sampling' OR 'ecological momentary assessment') NEAR/10 ('Bipolar*' OR 'Manic disorder*' OR 'depressi*' OR 'affective disorder*' OR 'mood disorder*')

**Google scholar search:** An additional search of the first 15 pages of Google Scholar was conducted (search terms: ‘mood track’, ‘ecological momentary assessment’, ‘monitoring’, ‘remote monitoring’, ‘active monitor’, ‘passive monitor’, ‘experience sample’, ‘experience sampling’)

Finally, subject experts were approached to identify additional articles.

**Grey Literature:**

**ProQuest Dissertations & Theses Global:**

(("self monitor" OR "self monitoring" OR "self monitors") OR ("self assess" OR "self assessed" OR "self assessment") OR ("self manage" OR "self managed" OR "self managing") OR ("self record" OR "self recorded" OR "self recording") OR “self surveillance” OR “patient* monitor*” OR ("measurement technologies" OR "measurement technology") OR “telemonitor*” OR ("remote monitoring") OR ("passive monitoring") OR ("active monitoring") OR “mood track*” OR “mood monitor*” OR ("experience sampling") OR “ecological momentary assessment”) NEAR/10 (("bipolar disorder" OR "bipolar disorders") OR ("manic depression" OR "manic depressive") OR “Bipolar affective psychos*” OR ("bipolar depression") OR “Manic disorder*” OR “depressi*” OR ("depressive disorder") OR “major depressive disorder*” OR “depression” OR ("affective disorder" OR "affective disorders") OR ("mood disorder" OR "mood disorders"))

<http://abc.cardiff.ac.uk/login?url=https://www.proquest.com/search/2332884?accountid=9883>

<https://www.proquest.com/pqdtglobal>

**ProQuest SciTech Premium Collection:**

(("self monitor" OR "self monitoring" OR "self monitors") OR ("self assess" OR "self assessed" OR "self assessment") OR ("self manage" OR "self managed" OR "self managing") OR ("self record" OR "self recorded" OR "self recording") OR “self surveillance” OR “patient* monitor*” OR ("measurement technologies" OR "measurement technology") OR “telemonitor*” OR ("remote monitoring") OR ("passive monitoring") OR ("active monitoring") OR “mood track*” OR “mood monitor*” OR ("experience sampling") OR “ecological momentary assessment”) NEAR/10 (("bipolar disorder" OR "bipolar disorders") OR ("manic depression" OR "manic depressive") OR “Bipolar affective psychos*” OR ("bipolar depression") OR “Manic disorder*” OR “depressi*” OR ("depressive disorder") OR “major depressive disorder*” OR “depression” OR ("affective disorder" OR "affective disorders") OR ("mood disorder" OR "mood disorders"))

http://nottingham.idm.oclc.org/login?url=https://www.proquest.com/search/2332894?accountid=8018

<https://www.proquest.com/scitechpremium/>

**Google incognito mode – first 200 results:**

No new papers identified

**Systematic Reviews reference checked:**

These are cited in the main paper

PRISMA flow diagram of included studies

**Identification of studies via databases and registers**

Records removed *before screening*:

Duplicate records removed (n = 1119)

Records identified from*:

Databases (n = 23,515)

**Identification**

Records screened

(n = 22,396)

Records excluded**

(n = 21,638)

Reports sought for retrieval

(n = 758)

Reports not retrieved

(n = 0)

**Screening**

Reports excluded:

Wrong publication type: 106

Wrong outcome: 207

Wrong population: 220

Wrong study design: 168

Duplicate paper/data: 16

Reports assessed for eligibility

(n = 758)

Studies included in review

(n = 41)

**Included**

*Consider, if feasible to do so, reporting the number of records identified from each database or register searched (rather than the total number across all databases/registers).

**If automation tools were used, indicate how many records were excluded by a human and how many were excluded by automation tools.

PRISMA Checklist

| **Section/topic** | **#** | **Checklist item** | **Reported on page #** |
| --- | --- | --- | --- |
| **TITLE** | | |  |
| Title | 1 | Identify the report as a systematic review, meta-analysis, or both. | 1 |
| **ABSTRACT** | | |  |
| Structured summary | 2 | Provide a structured summary including, as applicable: background; objectives; data sources; study eligibility criteria, participants, and interventions; study appraisal and synthesis methods; results; limitations; conclusions and implications of key findings; systematic review registration number. | 1 |
| **INTRODUCTION** | | |  |
| Rationale | 3 | Describe the rationale for the review in the context of what is already known. | 2 |
| Objectives | 4 | Provide an explicit statement of questions being addressed with reference to participants, interventions, comparisons, outcomes, and study design (PICOS). | 3 |
| **METHODS** | | |  |
| Protocol and registration | 5 | Indicate if a review protocol exists, if and where it can be accessed (e.g., Web address), and, if available, provide registration information including registration number. | 3 |
| Eligibility criteria | 6 | Specify study characteristics (e.g., PICOS, length of follow-up) and report characteristics (e.g., years considered, language, publication status) used as criteria for eligibility, giving rationale. | 3 |
| Information sources | 7 | Describe all information sources (e.g., databases with dates of coverage, contact with study authors to identify additional studies) in the search and date last searched. | 3, 145 |
| Search | 8 | Present full electronic search strategy for at least one database, including any limits used, such that it could be repeated. | 145 |
| Study selection | 9 | State the process for selecting studies (i.e., screening, eligibility, included in systematic review, and, if applicable, included in the meta-analysis). | 145 |
| Data collection process | 10 | Describe method of data extraction from reports (e.g., piloted forms, independently, in duplicate) and any processes for obtaining and confirming data from investigators. | 3 |
| Data items | 11 | List and define all variables for which data were sought (e.g., PICOS, funding sources) and any assumptions and simplifications made. | 3 |
| Risk of bias in individual studies | 12 | Describe methods used for assessing risk of bias of individual studies (including specification of whether this was done at the study or outcome level), and how this information is to be used in any data synthesis. | 3 |
| Summary measures | 13 | State the principal summary measures (e.g., risk ratio, difference in means). | 3 |
| Synthesis of results | 14 | Describe the methods of handling data and combining results of studies, if done, including measures of consistency (e.g., I^2^) for each meta-analysis. | 3 |
